# 2D conjugated microporous polyacetylenes synthesized via halogen-bond-assisted radical solid-phase polymerization for high-performance metal-ion absorbents

**DOI:** 10.1038/s41467-023-35809-5

**Published:** 2023-01-12

**Authors:** Hong Tho Le, Chen-Gang Wang, Atsushi Goto

**Affiliations:** 1 School of Chemistry, Chemical Engineering and Biotechnology, Nanyang Technological University, 62 Nanyang Drive, 637459 Singapore, Singapore; 2 Institute of Sustainability for Chemicals, Energy and Environment (ISCE2), Agency for Science Technology and Research (A*STAR), 2 Fusionopolis Way, 138634 Singapore, Singapore

**Keywords:** Polymer synthesis, Polymers, Polymers

## Abstract

The paper reports the first free-radical solid-phase polymerization (SPP) of acetylenes. Acetylene monomers were co-crystalized using halogen bonding, and the obtained cocrystals were polymerized. Notably, because of the alignment of acetylene monomers in the cocrystals, the adjacent C≡C groups were close enough to undergo radical polymerization effectively, enabling the radically low-reactive acetylene monomers to generate high-molecular-weight polyacetylenes that are unattainable in solution-phase radical polymerizations. Furthermore, the SPP of a crosslinkable diacetylene monomer yielded networked two-dimensional conjugated microporous polymers (2D CMPs), where 2D porous polyacetylene nanosheets were cumulated in layer-by-layer manners. Because of the porous structures, the obtained 2D CMPs worked as highly efficient and selective adsorbents of lithium (Li^+^) and boronium (B^3+^) ions, adsorbing up to 312 mg of Li^+^ (31.2 wt%) and 196 mg of B^3+^ (19.6 wt%) per 1 g of CMP. This Li^+^ adsorption capacity is the highest ever record in the area of Li^+^ adsorption.

## Abstract (web summary)

Polyacetylenes are an important class of conjugated polymers but are generally synthesized via metal-catalyzed coordination polymerization leaving trace metals as impurities. Here the authors demonstrate a free radical polymerization of co-crystalized acetylene monomers without the need of a metal catalyst.

## Introduction

Conjugated polymers have gained interest in various applications such as electronic and optical applications^1–10^. Polyacetylenes (–(CR=CR)_*n*_–) are representative conjugated polymers. While polyacetylenes are generally synthesized from acetylene monomers (CR≡CR) via metal-catalyzed coordination polymerization in solutions^11,12^, a metal-free synthetic method is desired for applications in which metal contaminants are undesirable.

Apart from linear conjugated polymers, two-dimensional conjugated microporous polymers (2D CMPs) are another type of conjugated polymers, in which conjugated polymer sheets are non-covalently or covalently cumulated in layer-by-layer manners to form regulated and rigid porous structures^13–17^. 2D CMPs have found various applications in gas separation, energy storage, and selective sieving of metal ions from a mixture of ions^18–21^. There are numerous synthetic studies of 2D CMPs using metal-catalyzed reactions^11^. Metal-free reactions such as Schiff-base condensation and oxidative coupling have also been utilized^11,22^, but applicable reactions are still limited. The exploration of efficient methods to synthesize polyacetylenes and 2D CMPs without using metallic catalysts is highly desirable but is also a challenging research theme.

Radical polymerization is catalyst-free. However, radical polymerization is effective for vinyl monomers (CH_2_=CR_2_) but not for acetylene monomers in solutions, because the reactivity of the propagating radical to acetylene monomers is very low (2–5 orders of magnitude lower than that to vinyl monomers) to yield only oligomers in solutions^23^. In our previous work, we developed a halogen-bond (XB)-assisted free-radical solid-phase polymerization (SPP). Several vinyl monomers were converted to solid cocrystals via XB, and the resultant alignment of the monomers facilitated radical propagation to yield high-molecular-weight vinyl polymers^24,25^.

**Fig. 1:**
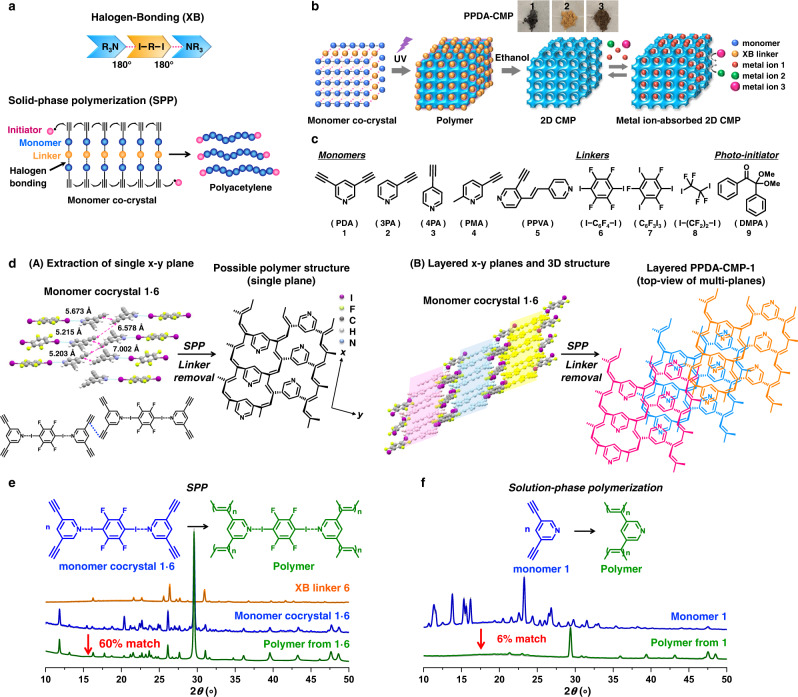
Schematic illustration of XB-assisted SPP, compounds used in this work, monomer cocrystal and polymer structures, and PXRD patterns before and after polymerization. **a** Schematic illustration of XB-assisted SPP of acetylene. **b** Schematic illustration of polyacetylene synthesis, 2D CMP formation after linker removal, and metal ion adsorption of 2D CMP. The inserted photos show PPDA-CMP-1, 2, and 3 generated from monomer cocrystals **1·6**, **1·7**, and **1·8**, respectively (after linker removal). **c** Monomers, linkers, and photo-initiator used in this work. **d** (**A**) Monomer cocrystal structure of **1·6** determined by single-crystal X-ray diffraction and a possible polymer structure expected from the monomer cocrystal structure. The figure extracts a single *x*-*y* plane of the monomer cocrystal and its possible polymer structure, which is a ladder-shaped polymer growing on the *x*-axis. The ladder-shaped polymer is further connected to the neighboring ladder-shaped polymers on the *y*-axis, forming a nanosheet in the *x*-*y* plane. The discussion of the polymer structure is given in Supplementary Information (Supplementary section 2.2 and Supplementary Fig. 1). **B** A possible multilayer structure of PPDA-CMP-1, showing that the nanosheets form a layer-by-layer structure on the *z*-axis. **e** PXRD patterns of pure XB linker **6** (orange), monomer cocrystal **1·6** (blue), and the polymer obtained from **1·6** via SPP (green), and their calculated PXRD patterns (in gray color and overlapped with the experimental spectra). 60% of the PXRD pattern of the polymer matched that of the monomer cocrystal. **f** PXRD patterns of pure solid monomer **1** (blue) and the polymer obtained from **1** via solution-phase polymerization (green), and their calculated PXRD patterns (gray). 6% of the PXRD pattern of the polymer matched that of the solid monomer.

In the present work, we used acetylene monomers in XB-assisted free-radical SPP and attempted to obtain polyacetylene polymers (not oligomers) (Fig. 1a). Because of the alignment of acetylene monomers in the cocrystals, the adjacent C≡C groups can be close enough to undergo effective polymerization, which is not attainable in solutions. This work opens up an effective synthesis of polyacetylenes via radical polymerization, giving significant scientific and practical impacts. Experimentally, we used mono-acetylenes (HC≡CR) to obtain linear polymers and a di-acetylene (HC≡C–R–C≡CH with an R spacer) to obtain network polymers (2D CMPs) (Fig. 1b and c). Goroff et al. reported a solid-phase topochemical polymerization of a halogen-bond-based diiododiacetylene (I–C≡C–C≡C–I without an R spacer) monomer cocrystal^26^, where the monomers were aligned in topochemical distances to enable topochemical 1,4-addition polymerization and yield poly(diiododiacetylene). The synthesis of 2D CMPs via radical polymerization is unprecedented. Unlike hydrogen bonds, XB is highly directional with a bonding angle of nearly 180° (Fig. 1a) and can assemble acetylene monomers into supramolecular structures in directional manners^27–32^. After the polymerization and XB linker removal (Fig. 1b), 2D CMPs with regulated pores were created. The pore sizes can be modulated by changing XB linkers.

**Fig. 2:**
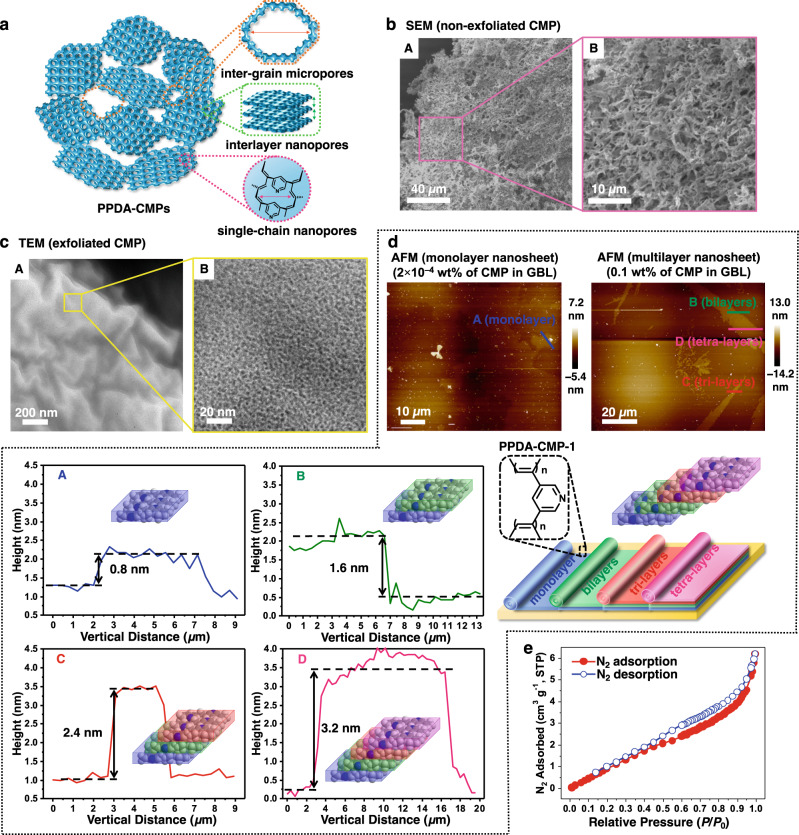
Porous structures, 2D exfoliated structures, and BET analysis of PPDA-CMP. **a** Schematic illustration of three types of pores in CMPs. **b** SEM images of non-exfoliated PPDA-CMP-1 showing inter-grain micropores. **c** TEM images of exfoliated PPDA-CMP-1 (at 2 × 10^–4^ wt% of CMP in GBL) showing surface (image **A**) and single-chain nanopores (zoom-in image **B**) at the outermost layer of CMP. **d** AFM images and schematic illustration of exfoliated PPDA-CMP-1 (at 2 × 10^–4^ wt% and 0.1 wt% of CMP in GBL) with (**A**) monolayer (blue), (**B**) bilayer (green), (**C**) tri-layer (red), and (**D**) tetra-layer (pink) nanosheets. **e** BET analysis with a nitrogen (N_2_) adsorption-desorption isotherm of non-exfoliated PPDA-CMP-1.

In the present work, we also studied metal ion adsorption of the obtained 2D CMPs as an interesting application. Adsorption is a low-cost, effective, and sustainable (low energy-consuming) technique that can be used for metal ion separation. We studied the adsorption of lithium ion (Li^+^) and boronium ion (B^3+^). Because of its high value, the collection of Li^+^ from seawater reverse osmosis (SWRO) brines is an urgent need in desalination and can be a promising application^33^. Boron is a pollutant in the environment, potentially affecting human health and causing photosynthesis inhibition in plants^34–36^. Therefore, the collection of Li^+^ and B^3+^ from SWRO brines and wastewaters can be an important application^37–40^. Furthermore, our 2D CMPs are highly durable for recycled use, and the pore sizes are tuneable by XB linkers for selective absorption of particular metal ions. Our 2D CMPs are purely organic (metal-free) and can be environmentally friendly absorbents.

## Results

### Preparations of monomer cocrystals via XB

The studied monomers (Fig. 1c) are pyridyl-3,5-diacetylene (PDA) (**1**), 3-pyridylacetylene (3PA) (**2**), 4-pyridylacetylene (4PA) (**3**), pyridyl-2-methyl-5-acetylene (PMA) (**4**), and (E)-pyridyl-4-[2-(4-pyridinyl)vinyl]-3-acetylene (PPVA) (**5**). The studied XB linkers (Fig. 1c) are 1,4-diiodotetrafluorobenzene (I−C_6_F_4_−I) (**6**), 1,3,5-trifluoro-2,4,6-triiodotrifluorobenzene (C_6_F_3_I_3_) (**7**), and 1,2-diiodotetrafluoroethane (I−(CF_2_)_2_−I) (**8**). XB can be formed between a nitrogen (N) atom in the monomer and an iodine (I) atom in the linker. All of the compounds **1**−**8** are commercially available. We mixed a monomer (1−3 eq.), a linker (1 eq.), and a photo-initiator (2,2-dimethoxy-2-phenyl-acetophenone (DMPA) (**9**) (Fig. 1c)) (0.67 eq.) in dichloromethane (solvent), setting equimolar atoms of N and I for stoichiometric XB formation. We evaporated the solvent from the mixture and obtained solids in gram scales. We studied the combinations of **1** with **6** (**1·6**), **7** (**1·7**), and **8** (**1·8**), **2** with **6** (**2·6**) and **7** (**2·7**), **3** with **6** (**3·6**), **4** with **6** (**4·6**), and **5** with **6** (**5·6**). The combinations **1·7** and **1·8** did not form single cocrystals, generating polycrystalline solids. The other six combinations generated single cocrystals, and their single-crystal X-ray diffraction analyses showed that the cocrystals contained the monomer and linker. DMPA was not observed in the single-crystal X-ray structures, meaning that the monomer and linker formed cocrystals (78.0–82.7 wt%) that were dispersed in amorphous DMPA (17.3–22.0 wt%). The aromatic linkers (**6** and **7**) were regularly aligned via the π-π stacking, and the monomers were thereby aligned along with the linkers via XB (Fig. 1d and Supplementary Figs. 2–7). The carbon-carbon distances between two adjacent C≡C groups (3.4–7.0 Å in Supplementary Table 1) were sufficiently short for the propagation to occur in all cases. The distances (3.4–7.0 Å) are not interatomic distances for reactions (van der Waals radii typically <4.0 Å for an effective orbital overlap) but interatomic distances between the two reactive carbons in the cocrystal lattices. In topochemical polymerizations, the interatomic distances in the cocrystal lattices are close to van der Waals radii. The present polymerization is not topochemical polymerization but free-radical polymerization. The propagating polymer chain end would move to approach the neighboring monomer, although the exact motion is not clear. Upon the polymerization, the interatomic distances would become shorter, resulting in slight deformation in the cocrystal structure.

### Radical SPP of monomer cocrystals

The obtained monomer/linker/photo-initiator three-component solids (called monomer cocrystals below) were exposed to UV light (*λ* = 365 nm) under an argon atmosphere for 40 h at room temperature (22 °C). As mentioned, DMPA (photo-initiator) was not incorporated in the monomer/linker cocrystal lattices but located in gaps among the cocrystal grains (outside the cocrystals). Upon irradiation, DMPA decomposed, and the radicals were generated outside the cocrystals. The radicals subsequently entered into the cocrystals through the cocrystal surfaces. The propagation occurred from one face to the center and to the counter face in the cocrystals.

We studied the SPP of **1·6** with varying amounts of DMPA (0.01–0.67 equiv to linker and 0.005–0.335 equiv to monomer). No polymer was generated with the amounts of DMPA at 0.005 and 0.05 equivs to monomer, whereas the polymer was obtained with a 100% monomer conversion at 0.335 equiv to monomer after 40 h, suggesting that this is an optimal SPP condition (Supplementary Table 2). The amount of DMPA (0.335 equiv to monomer) was large, because the lifetime of radicals is short (is not sufficient for radicals to diffuse) and only the DMPA located close to the cocrystal surfaces could generate effective radicals that can enter into the cocrystals. A majority of the DMPA apart from the cocrystal surfaces would not enter the cocrystals, and therefore an excess of DMPA was required in the present study. The exact diffusivity of radicals is not clear at this moment.

The SPP of **1·6** (actually containing **1**, **6**, and DMPA) led to a 100% monomer conversion. After the SPP, the solid was stirred in ethanol and divided into ethanol-soluble (3 wt%) and ethanol-insoluble (97 wt%) polymers (Table 1, entry 1). The monomer (**1** (PDA)) is a diacetylene bearing two polymerizable C≡C groups, and hence the polymer can be branched or further crosslinked, giving an insoluble polymer. Thus, not only a soluble polymer with a peak-top molecular weight (*M*_p_) of 46000 but also an even higher-molecular-weight insoluble polymer (e.g., crosslinked polymer) was yielded. For comparison (Table 1, entry C1), we conducted a solution-phase radical polymerization of **1** using DMPA as a photo-initiator and dichloromethane as a solvent but without using **6** (linker), giving a significantly lower monomer conversion (=10%). The insoluble (high-molecular-weight) polymer was only 18 wt%, which was significantly lower than that obtained from the SPP (97 wt%). The result means the low reactivity of **1** in solution.

**Table 1:** Polymerizations of crystallized and non-crystallized monomers under UV light (*λ* = 365 nm) at room temperature for 40 h

Entry	Mode	M	L^a^	[M]_0_/[L]_0_/[DMPA]_0_	Monomer conversion (%)	*M*_p_^b^	Soluble polymer (wt%)	Insoluble polymer (wt%)
1	SPP	PDA (**1**)	**6**	2/1/0.67	100	46000	3	97
2	SPP	PDA (**1**)	**7**	3/1/0.67	99	17000	7	93
3	SPP	PDA (**1**)	**8**	2/1/0.67	97	12000	8	92
C1	Solution	PDA (**1**)	NA	2/0/0.67	10	15000	82	18
4	SPP	3PA (**2**)	**6**	2/1/0.67	100	13000	45	55
5	SPP	3PA (**2**)	**7**	3/1/0.67	87	5400	88	12
C2	Solution	3PA (**2**)	NA	2/0/0.67	0	NA	NA	NA
6	SPP	4PA (**3**)	**6**	2/1/0.67	100	6300	85	15
C3	Solution	4PA (**3**)	NA	2/0/0.67	100	5000	89	11
7	SPP	PMA (**4**)	**6**	2/1/0.67	100	5300	91	9
C4	Solution	PMA (**4**)	NA	2/0/0.67	3	5200	100	0
8	SPP	PPVA (**5**)	**6**	1/1/0.67	100	5400	88	12
C5	Solution	PPVA (**5**)	NA	2/0/0.67	50	5000	90	10

Bold values indicate the numbering of the monomers and linkers used in the solid-phase polymerization.

DMF was used as the GPC eluent. *M*_p_ is the peak-top molecular weight. Because of the presence of oligomers and possible clusters of LiBr contained in the DMF eluent, the GPC baseline was not horizontal in all cases. Hence, the number-average molecular weight (*M*_n_) and dispersity (*Đ*) values were not accurately determined, and we studied the *M*_p_ value instead.

^a^L = XB linker.

^b^Polystyrene (PSt)-calibrated DMF-GPC values for soluble polymers in ethanol, where GPC is gel permeation chromatography.

We further studied SPPs for other monomers and linkers. All studied SPPs (**1·7**, **1·8**, **2·6**, **2·7**, **3·6**, **4·6**, and **5·6**) led to high monomer conversions (87–100%) (Table 1, entries 2–8). Polymers synthesized from **1·7** to **1·8** comprised large fractions (93 and 92 wt%) of insoluble polymers, indicating the dominant formation of high-molecular-weight (crosslinked) polymers (Table 1, entries 2 and 3). Monomers **2**–**5** are mono-acetylenes and hence yielded only linear (non-crosslinked) polymers. Nevertheless, monomer cocrystals **2·6**, **2·7**, **3·6**, **4·6**, and **5·6** still generated 9–55 wt% of insoluble polymers (Table 1, entries 4–8), which would be high-molecular-weight linear polyacetylenes. The solution-phase polymerizations of monomers **2**–**5** (Table 1, entries C2–C5) resulted in no or slow polymerizations or less insoluble fractions than those in the SPP. These results demonstrate more efficient polymerizations in the solid states. During the SPP, the color changed from white (monomer cocrystals) to brown because of the generation of polyacetylenes (Fig. 1b). The slightly different colors in the polymers generated from **1·6**, **1·7**, and **1·8** (Fig. 1b) would be ascribed to different polymer structures (propagation patterns) brought by different linkers **6**, **7**, and **8**. The reaction mode of acetylenes may be continuous radical chain propagation to form polymers (polymerization) or discontinuous radical addition to form dimers (dimerization). If the discontinuous radical addition occurs, the reactions of mono-acetylenes (monomers **2**–**5**) will give only dimers. In the present systems, we actually obtained polymers (as described above), demonstrating that the reaction mode was continuous radical chain propagation (polymerization). For all studied SPPs (**1·6**, **1·7**, **1·8**, **2·6**, **2·7**, **3·6**, **4·6**, and **5·6**), without using DMPA (photo-initiator), no polymerization took place under the UV irradiation (Supplementary Figs. 8–15), meaning that no topochemical polymerizations took place in the studied cocrystals. The result confirms that the observed SPP (Table 1, entries 1–8) is ascribed to radical polymerization initiated by DMPA.

The obtained polymers did not maintain single-crystal structures but became polycrystalline solids. This is because the neighboring monomer-monomer distances in the cocrystals were non-topochemical distances and became shorter by the polymerization, which would bring some deformation of cocrystal structures. Thus, instead of single-crystal X-ray diffraction, we used powder X-ray diffraction (PXRD) to study the change in the crystallinity from the monomer cocrystals to the polymer solids. From PXRD, we determined the diffraction pattern matching before and after the SPP. For **1·6** (Fig. 1e), 60% of the diffraction peaks remained even after the SPP (Supplementary Table 3, entries 1 and 2), demonstrating that the monomer cocrystal structure was relatively largely retained even after the SPP. For comparison, we analyzed a polymer solid of **1** synthesized in the solution phase and the original monomer solid of **1** (Fig. 1f). The PXRD pattern showed only a 6% matching (Supplementary Table 3, entries 3 and 4), suggesting that the polymer solid was mostly amorphous. The polymer solids obtained from **1·7**, **1·8**, **2·6**, and **2·7** via the SPP also had relatively high matchings (43–79%) (Supplementary Table 3, entries 5–12, and Supplementary Fig. 16). The results qualitatively show that the cocrystal structures were relatively largely retained during the SPP and that the polymerizations were crystal-to-crystal polymerizations with some deformation of the cocrystal structures, giving polycrystalline solids.

In the solid phase, propagation can occur only within a cocrystal grain. Thus, the grain size would decide the maximum chain length (maximum molecular weight), which would correspond to a distance from one face to the counter face in the grain. The grain sizes of cocrystals **1·6**, **1·7**, and **1·8** were ~500–1000 nm, according to the transmission electron microscopy (TEM) analysis (Supplementary Figs. 23–25). The grains would be single cocrystals or assemblies of cocrystals, which were embedded in the DMPA (photo-initiator) matrix. The grain sizes were virtually the same before and after the SPP (Supplementary Figs. 23–25), suggesting that the origin of the grains of the formed polymers (below) is the grains of the monomer cocrystals.

After the SPP, the polymerized **1·6**, **1·7**, **1·8**, **2·6**, and **2·7** cocrystals were purified (washed) with ethanol to remove the linkers. After the linker removal, porous polymers and hence CMPs were obtained from **1·6**, **1·7**, and **1·8** (Fig. 2a), which are termed PPDA-CMP-1, PPDA-CMP-2, and PPDA-CMP-3, respectively. Here, PPDA is poly(pyridyl-3,5-diacetylene). The Fourier transform infrared (FTIR) spectra of the CMPs (Supplementary Figs. 18–20) showed peaks at 1698–1721 cm^–1^ for non-aromatic C=C bonds (polyacetylene backbone), as separated from the peaks at 1560–1639 cm^–1^ for aromatic (pyridine) C=C bonds. The alkene C–H stretch (=C–H stretch) in the polyacetylene backbone also appeared at 2968–3047 cm^–1^. The results confirm the formation of the polyacetylene backbone. Besides nanometer-sized pores as described below, there were micrometer-sized pores (inter-grain micropores) (Fig. 2a), as observed with scanning electron microscopy (SEM) (Fig. 2b). The obtained polymers formed multiple grains (Fig. 2a), and the grain sizes of the polymers corresponded to the grain sizes of the monomer cocrystals (as mentioned above) or were slightly larger due to possible fusion of the grains. The gaps between the grains were enlarged in the washing process (via the swelling in ethanol), which would generate the observed micrometer-sized pores. The thermogravimetric analysis of the three CMPs showed that their 50% weight loss decomposition temperatures (*T*_d(50%)_) were 363–667 °C (Supplementary Figs. 31–33), demonstrating their high thermal stability. In addition, the FTIR study also showed that the linear polymers obtained from **2·6** to **2·7** contained polyacetylene backbones (Supplementary Figs. 21 and 22).

The electronic conductivities (*σ*) of PPDA-CMP-1 before and after iodine (I_2_) doping were measured at room temperature. The *σ* value increased from 2.4 × 10^–9^ S cm^–1^ (before the doping) to 2.7 × 10^–4^ S cm^–1^ after the doping (Supplementary Table 4, entry 1). For comparison, the *σ* value of the PPDA synthesized in the solution phase (Table 1, entry C1) increased from ≤10^–9^ S cm^–1^ (below detection limit) to 8.3 × 10^–5^ S cm^–1^ after the doping (Supplementary Table 4, entry 2). Both before and after the doping, the *σ* values of PPDA-CMP-1 were larger than those of the PPDA synthesized in the solution phase, because PPDA-CMP-1 had a longer π-conjugation (a higher-molecular weight) than the PPDA synthesized in the solution phase. The porous structure of PPDA-CMP-1 could also enhance the adsorption of I_2_ vapor during the doping process, increasing electron carrier mobility. The observed *σ* value (2.7 × 10^–4^ S cm^–1^) of the doped PPDA-CMP-1 is not remarkably high but still comparable with those of doped substituted polyacetylenes (*σ* = 10^–4^–10^2^ S cm^–1^)^41^, and the doped PPDA-CMP-1 would be categorized to a semiconducting polymer.

### 2D exfoliated structures of PPDA-CMPs

The three CMPs were dispersed in *γ*-butyrolactone (GBL) at two different concentrations (0.1 and 2 × 10^–4^ wt% of CMP in GBL). After gentle stirring at 50 °C for 5 days, exfoliation of 2D polymer nanosheets was observed (as described below), demonstrating that the obtained CMPs were 2D CMPs. We observed exfoliation in GBL but not in ethanol. We used ethanol to remove the linkers (as mentioned above) and used GBL to exfoliate polymer sheets because PPDA is more miscible in GBL.

For PPDA-CMP-1 (Fig. 1d and Supplementary Fig. 26), based on the monomer cocrystal (**1·6**) structure, ladder-shaped polymer chains would grow on the *x*-axis and be further connected to the neighboring ladder-shaped polymer chains on the *y*-axis, forming nanosheets in the *x*-*y* plane (Fig. 1d (A)). The nanosheets would be bridged by the XB linkers to form layer-by-layer nanosheet structures on the *z*-axis. When the linkers were removed in ethanol, gaps would be generated between the nanosheets but the nanosheets would still be associated. In GBL, the nanosheets could be exfoliated because of the increased miscibility of the nanosheets in GBL. Because of the rigid polyacetylene backbones and ladder-shaped structures, the resultant nanosheets were rigid and retained the 2D structures even after the linker removal. For PPDA-CMP-2 and PPDA-CMP-3, exact polymer structures are not deducible because single cocrystals of **1·7** and **1·8** were not obtained, but ladder-shaped (nano-porous) polymers were likely generated as observed with TEM (below) (Supplementary Figs. 27b and 28b).

For the three CMPs, the dispersions of CMPs were drop-casted on Cu grids and Si wafers and characterized using TEM and atomic force microscopy (AFM), respectively. The TEM images of CMPs (dispersed at 2 × 10^–4^ wt%) showed exfoliated multi-layered nanosheets (Fig. 2c (image A) and Supplementary Figs. 26 (image A), 27b (images A and B), and 28b (images A and B)) and nano-porous structures on the outermost surface of the nanosheets (Fig. 2c (image B) and Supplementary Figs. 26 (image B), 27b (images C and D), and 28b (images C and D)) for all three CMPs. The pore sizes were 1.1, 0.9, and 1.0 nm for PPDA-CMP-1, 2, and 3, respectively. There were three types of pores with different sizes (Fig. 2a). The first pores are nanometer-sized pores corresponding to voids in the ladder-shaped polymer chains (single-chain nanopores). The pores observed in the TEM images most likely correspond to the single-chain nanopores. The second pores were nanometer-sized pores generated between the nanosheets during the linker removal (interlayer nanopores). The interlayer distances would depend on the nanosheet structures. The linkers can influence both single-chain and nanosheet structures and hence modulate the sizes of the single-chain and interlayer nanopores. The third micrometer-sized pores result from micrometer-sized gaps between different crystal grains in the polycrystalline polymer solids (inter-grain pores), as mentioned above. Thus, we observed the single-chain nanopores using TEM and the inter-grain micropores using SEM, as described above, and also all three pores using Brunauer-Emmett-Teller (BET) analysis, as described below.

We analyzed the PPDA-CMP-1 nanosheet exfoliated at 2 × 10^–4^ wt% using AFM and observed a mono-layer structure with a thickness of ~0.8 nm (Fig. 2d). At the higher exfoliation concentration (0.1 wt%) of PPDA-CMP-1, multilayer structures, i.e., bi-, tri-, and tetra-layered structures were observed with thicknesses of 1.6, 2.4, and 3.2 nm, respectively (Fig. 2d and Supplementary Fig. 34). Similarly, exfoliated PPDA-CMP-2 and PPDA-CMP-3 showed mono-layer structures with thicknesses of 0.5–0.6 nm and also bi- to undeca-layered multilayer structures (Supplementary Figs. 35 and 36). It should be noted that all three CMPs underwent partial exfoliations (not full exfoliations) in GBL, probably because PPDA nanosheets are not fully miscible in GBL or some nanosheets are covalently bonded during the polymerization (SPP) because of possible defects in the cocrystals.

### BET analysis

We determined the surface areas of non-exfoliated PPDA-CMP-1, 2, and 3 (after removal of linkers but not exfoliation) using the BET method. Figure 2e shows the nitrogen (N_2_) adsorption isotherm of PPDA-CMP-1 studied at the relative pressure (*P*/*P*_0_) from 0 to 1 at –196 °C, where *P*_0_ is the saturated pressure of adsorbent (N_2_). The estimated specific surface area (*S*_BET_) was 13 m^2^ g^–1^ (Supplementary Fig. 37a and Supplementary Table 5, entry 1). The estimated pore size (*d*_BET_) was 2.9 nm (Supplementary Table 5, entry 1), which is slightly larger than that estimated from the TEM analysis (1.1 nm). This is because the non-exfoliated CMP contained all three (single-chain, interlayer, and inter-grain) pores and their average pore size was determined by the BET analysis, while the exfoliated CMP contained only the single-chain pore and its size was determined by the TEM analysis.

The BET analysis showed that PPDA-CMP-2 and PPDA-CMP-3 had *S*_BET_ values of 26 and 11 m^2^ g^–1^, respectively, and *d*_BET_ values of 7.1 and 17 nm, respectively (Supplementary Figs. 37b and 37c and Supplementary Table 5, entries 2 and 3). These *d*_BET_ values (7.1 and 17 nm) are much larger than those (0.9 and 1.0 nm) estimated from the TEM analysis, suggesting significant contributions of interlayer and inter-grain pores for PPDA-CMP-2 and PPDA-CMP-3. Thus, using the same monomer (PDA (**1**)) but using different XB linkers (**6**–**8**), we were able to vary CMP structures with respect to surface areas and pore sizes, demonstrating tuneable CMP structures driven by XB.

### Applications in high-performance metal-ion adsorption

**Fig. 3:**
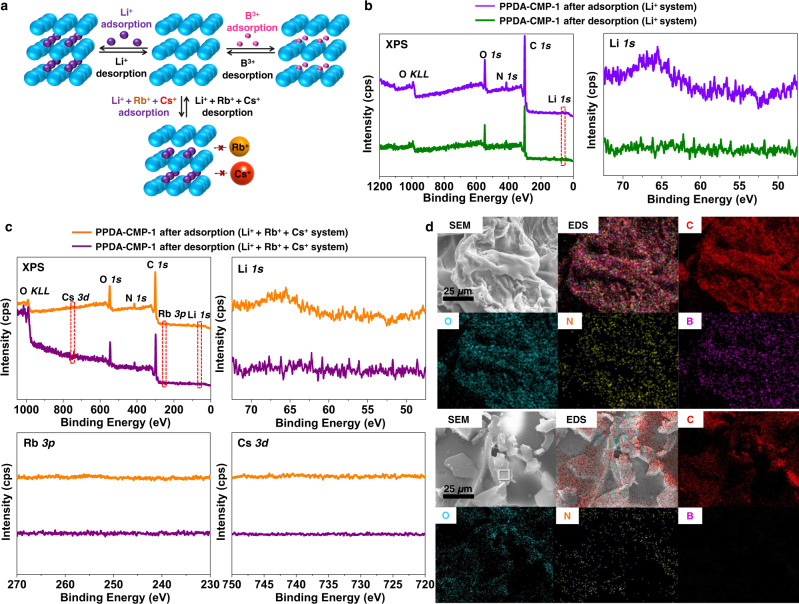
Metal ion adsorption-desorption in PPDA-CMP. **a** Schematic illustration of adsorption-desorption of Li^+^ (left) and B^3+^ (right) in PPDA-CMP. Li^+^ is selectively adsorbed from a mixture of Li^+^, Rb^+^ and Cs^+^ in PPDA-CMP-1 (bottom). **b** Full and Li *1* *s* XPS spectra of PPDA-CMP-1 after Li^+^ adsorption (purple lines) and desorption (green lines) for the pure Li^+^ system (Table 2, entry 1). **c** Full, Li *1* *s*, Rb *3p*, and Cs *3d* XPS spectra of PPDA-CMP-1 after metal ion adsorption (orange lines) and desorption (purple lines) for the Li^+^ + Rb^+^ + Cs^+^ system (Table 2, entry 4). **d** SEM-EDS mapping images of PPDA-CMP-1 after B^3+^ adsorption (top) and desorption (bottom) for the pure B^3+^ system (Table 2, entry 7); electron images (SEM, gray), summarized EDS images (EDS, multi-colors), and individual element images (carbon (C, red), oxygen (O, blue), nitrogen (N, yellow), and boron (B, purple)).

We studied ion adsorption of non-exfoliated PPDA-CMP-1, 2, and 3 in water (Fig. 3a). The studied ions were alkaline metal ions with different sizes, i.e., Li^+^, rubidium (Rb^+^), and cesium (Cs^+^) ions, and a group 13 ion, i.e., B^3+^. PPDA contains electron-donating nitrogen atoms, which can coordinate those cations. PPDA-CMP-1 (12.5 mg) was immersed in an aqueous solution (50 mL) containing Li^+^ (0.1 wt%), sonicated for 1 h, and left overnight at room temperature. We measured Li^+^ concentrations in the aqueous solution before and after the adsorption using inductively coupled plasma optical emission spectrometry (ICP-OES), showing that PPDA-CMP-1 adsorbed 312 mg of Li^+^ per 1 g of CMP (31.2 wt% Li^+^ adsorption) (Table 2, entry 1 and Supplementary Table 6, entry 1). This value (31.2 wt% Li^+^ adsorption) is the highest record of adsorption capacity in the area of Li^+^ adsorption. To the best of our knowledge, the maximum Li adsorption capacity previously reported is 7.67 wt% using H_2_TiO_3_^42,43^. Also, markedly, PPDA-CMP-1 showed selective Li^+^ adsorption from a mixed solution of Li^+^, Rb^+^, and Cs^+^ (0.1 wt% for each). We observed only Li^+^ adsorption (30.6 wt% Li^+^ adsorption) but no Rb^+^ or Cs^+^ adsorption (Table 2, entry 4, and Supplementary Table 6, entry 4). (The adsorption of Cs^+^ was studied using X-ray photoelectron spectroscopy (XPS), as described below, because of insufficient sensitivity of ICP-OES to Cs^+^.) This selectivity is probably because the Li^+^···N coordination was stronger than the Rb^+^···N and Cs^+^···N coordination due to the higher charge density of Li^+^ and because aromatic rings (or conjugated carbons) might have Li^+^ capacities as observed in electrochemical systems^44–46^. The atomic diameter of Li^+^ (0.31 nm) is small enough for Li^+^ to be incorporated in single-chain pores (with 1.1 nm pore sizes according to the TEM analysis), rationalizing the Li^+^ adsorption. The atomic diameters of Rb^+^ (0.50 nm) and Cs^+^ (0.53 nm) are also small enough, but no incorporation was observed. In an aqueous solution of B^3+^ (0.1 wt%), PPDA-CMP-1 achieved 19.6 wt% B^3+^ adsorption (Table 2, entry 7, and Supplementary Table 6, entry 7). This value (19.6 wt% B^3+^ adsorption) is also among the highest adsorption capacities previously reported for B^3+^ (12.8–30.3 wt%)^35,36^. The atomic diameter of B^3+^ (0.17 nm) is also smaller than the single-chain pore size. Thus, PPDA-CMP-1 had the highest ever adsorption capacity of Li^+^, the notably high adsorption capacity of B^3+^, and perfect adsorption selectivity to Li^+^. The observed 31.2 wt% Li adsorption means an empirical formula Li_5.73_/PDA (monomer unit).

**Table 2:** Adsorption of metal ions by PPDA-CMP-1, 2, and 3 and PPDA synthesized in solution phase

Entry	CMP	Metal Solution (0.1 wt% (1000 ppm) of metal ion^+^)	Amount of metal ion adsorbed per CMP (mg/g CMP)^a^
1	PPDA-CMP-1	LiOH	312
2	PPDA-CMP-2	LiOH	84
3	PPDA-CMP-3	LiOH	228
C1	PPDA synthesized in solution	LiOH	54
4	PPDA-CMP-1	LiOH + RbOH + CsOH	306 (Li^+^) and 0 (Rb^+^)^b^
5	PPDA-CMP-2	LiOH + RbOH + CsOH	72 (Li^+^) and 4 (Rb^+^)
6	PPDA-CMP-3	LiOH + RbOH + CsOH	168 (Li^+^) and 84 (Rb^+^)
7	PPDA-CMP-1	LiOH + RbOH + CsOH	196
8	PPDA-CMP-2	NH_4_BF_4_	17
9	PPDA-CMP-3	NH_4_BF_4_	0
C2	PPDA synthesized in solution	NH_4_BF_4_	7

^a^The metal ion adsorption was determined with ICP-OES (Supplementary Table 6).

^b^The amount of Cs+ adsorbed per CMP was nearly zero according to the XPS analysis (Fig. 3c).

For comparison, we studied ion adsorption using PPDA synthesized in the solution phase. Because of no monomer alignment, nanosheets and hence single-chain nanopores would not be effectively generated, showing only 5.4 wt% Li^+^ adsorption and 0.7 wt% B^3+^ adsorption (Table 2, entries C1 and C2). These values are much smaller than those of PPDA-CMP-1 (30.6–31.2 wt% Li^+^ adsorption and 19.6 wt% B^3+^ adsorption (Table 2, entries 1, 4, and 7)), confirming that the enhanced adsorption capacity of PPDA-CMP-1 resulted from the extended nanosheets with single-chain nanopores generated by the alignment of monomers.

PPDA-CMP-2 had much lower ion adsorption capacities (7.2–8.4 wt% Li^+^ adsorption and 1.7 wt% B^3+^ adsorption (Table 2, entries 2, 5, and 8)) than PPDA-CMP-1 (30.6–31.2 wt% Li^+^ adsorption and 19.6 wt% B^3+^ adsorption (Table 2, entries 1, 4, and 7)). The lower adsorption capacities of PPDA-CMP-2 would be partly ascribed to its smaller single-chain nanopores (0.9 nm according to the TEM analysis) compared with that of PPDA-CMP-1 (1.1 nm according to the TEM analysis), leading to limited Li^+^ capacities of aromatic rings. The empirical formula was Li_1.32–1.54_/PDA (monomer unit), indicating Li^+^ was mostly absorbed via the Li^+^···N coordination.

PPDA-CMP-3 had 22.8 wt% of Li^+^ adsorption (Table 2, entry 3), which is between 31.2 wt% Li^+^ adsorption for PPDA-CMP-1 (Table 2, entry 1) and 8.4 wt% Li^+^ adsorption for PPDA-CMP-2 (Table 2, entry 2). Unlike PPDA-CMP-1, PPDA-CMP-3 did not show good selectivity in Li^+^ and Rb^+^ adsorption (16.8 wt% Li^+^ adsorption and 8.4 wt% Rb^+^ adsorption (Table 2, entry 6)) or did not absorb B^3+^ (0 wt% B^3+^ adsorption (Table 2, entry 9)). Although PPDA-CMP-1 and PPDA-CMP-3 have similar single-chain nanopores (1.1 nm and 1.0 nm diameters according to the TEM analysis, respectively), PPDA-CMP-3 showed poorer adsorption selectivity. As the BET analysis showed, the average pore sizes of single-chain pores, interlayer pores, and inter-grain pores were 2.9 and 17 nm for PPDA-CMP-1 and PPDA-CMP-3, respectively, and the large average pore size of PPDA-CMP-3 would allow adsorption of Rb^+^, which would result in poorer adsorption selectivity.

Because of the highest capacity and perfect selectivity for Li^+^ adsorption, we used PPDA-CMP-1 to study the desorption of Li^+^. The Li^+^-adsorbed PPDA-CMP-1 (Table 2, entry 1) was sonicated in an aqueous acidic solution (0.5 M HCl) for 30 min three times to desorb Li^+^, subsequently neutralized with water, and dried in an oven at 120 °C under vacuum overnight. The XPS analysis (Fig. 3b) showed that the Li *1* *s* peak at 65.9 eV (binding energy) that appeared before desorption (purple lines) nearly perfectly disappeared after desorption (green lines), meaning a complete removal of Li after desorption. The Li *1* *s* peak before desorption (purple lines) appeared small, but this is not because the Li content was small but because the XPS relative sensitivity factor of Li (0.025) is much smaller than those of other atoms (0.296 for C, 0.477 for N, and 0.711 for O)^47^. Figure 3c shows the XPS spectrum (orange lines) of the Li^+^-adsorbed PPDA-CMP-1 obtained from a Li^+^, Rb^+^, and Cs^+^ mixed solution (Table 2, entry 4). The Li *1* *s* peak appeared at 65.9 eV but neither Rb *3p* peak (230–271 eV, binding energy) nor Cs *3d* peak (716–757 eV, binding energy) appeared, confirming the selective adsorption of Li^+^. The XPS relative sensitivity factors of Rb (1.542) and Cs (7.041) are much larger than that of Li (0.025)^46^, and hence no appearance of Rb or Cs peaks means virtually no adsorption of Rb^+^ or Cs^+^. After the desorption, the Li *1* *s* peak disappeared (Fig. 3c (purple lines)), meaning a complete removal of Li^+^. We also studied the desorption of B^3+^ from the B^3+^-adsorbed PPDA-CMP-1 (Table 2, entry 7). The SEM-energy dispersive spectroscopy (SEM-EDS) mapping analysis (Fig. 3d) showed no B signal in the desorbed PPDA-CMP-1, while C, O, and N signals were clearly detected, meaning a complete removal of B^3+^.

We further studied the recycled use of PPDA-CMP-1 for ion adsorption (Table 3 and Supplementary Table 7). We adsorbed and desorbed Li^+^ using a solution solely containing Li^+^ (Table 3, entry 1), showing 32.0 and 30.8% wt% Li^+^ adsorption in the second and third cycles, respectively, which were similar values to 31.2 wt% Li^+^ adsorption in the first cycle (Table 2, entry 1 and Table 3, entry 1). For the mixed ion system (Table 2, entry 4 and Table 3, entry 2), PPDA-CMP-1 did not adsorb Rb^+^ but maintained perfect selectivity in the Li^+^ adsorption in all the three cycles, although a decreasing trend was observed in the Li^+^ adsorption capacity (from 30.6 wt% to 24.0 wt% and 18.8 wt% Li^+^ adsorption from the first to second and third cycles). PPDA-CMP-1 also attained cycled B^3+^ adsorption-desorption despite a decreasing trend in the B^3+^ adsorption capacity (from 19.6 wt% to 17.2 wt% and 9.6 wt% B^+^ adsorption from the first to second and third cycles) (Table 2, entry 7 and Table 3, entry 3). Thus, PPDA-CMP-1 is recyclable for at least three cycles of Li^+^ and B^3+^ adsorption.

**Table 3:** Adsorption of metal ions by PPDA-CMP-1 in three cycles

Entry	Cycle	Metal Solution (0.1 %wt (1000 ppm) of metal ion^+^)	Amount of metal ion adsorbed per CMP (mg/g CMP)^a^
	First	LiOH	312
1	Second	LiOH	320
	Third	LiOH	308
	First	LiOH + RbOH + CsOH	306 (Li) and 0 (Rb)^b^
2	Second	LiOH + RbOH + CsOH	240 (Li) and 0 (Rb)
	Third	LiOH + RbOH + CsOH	188 (Li) and 0 (Rb)
	First	NH_4_BF_4_	196
3	Second	NH_4_BF_4_	172
	Third	NH_4_BF_4_	96

^a^The metal ion adsorption was determined with ICP-OES (Supplementary Table 7).

^b^The amount of Cs+ adsorbed per CMP was nearly zero according to the XPS analysis (Fig. 3c).

## Discussion

In summary, we constructed acetylene monomer cocrystals using XB and successfully used them to achieve free-radical SPPs of acetylenes. Despite the low reactivities of acetylene monomers in radical polymerization, the proximity of the adjacent C≡C groups in the cocrystals enabled effective propagation. The SPPs of PDA (diacetylene) and subsequent removal of linkers yielded 2D CMPs consisting of polymer nanosheets cumulating in layer-by-layer manners. The presence of nanosheets was demonstrated by the exfoliation of the CMPs. The pore structures were modulated by the linkers. PPDA-CMP-1 had the highest ever adsorption capacity of Li^+^ (31.2 wt% Li^+^ adsorption), high adsorption capacity of B^3+^ (19.6 wt% B^3+^ adsorption), perfect adsorption selectivity to Li^+^, and recyclability for Li^+^ and B^3+^ adsorption. The resultant 2D CMPs are purely organic (metal-free) and can be environmentally friendly absorbents. This synthetic method would be applicable to a range of nitrogen-containing and other electron-donating acetylenes and diacetylenes to yield high-molecular and crosslinked polymers that are inaccessible in solution-phase polymerizations and may open up new materials.

## Methods

All methods can be found in Supplementary Information.

## Supplementary information

Supplementary Information

## Data Availability

All data needed to evaluate the conclusions in the paper are present in the paper and/or the Supplementary Information. Additional data related to this paper may be requested from the corresponding authors.

## Code availability

All single-crystal data generated in this study have been deposited in the Cambridge Crystallographic Data Centre (CCDC) database. The access can be obtained free of charge from https://www.ccdc.cam.ac.uk/structures/. The single-crystal data generated in this study are provided in the crystallographic information files (CIF). The single-crystal data of the monomer co-crystals **1·6**, **2·6**, **2·7**, **3·6**, **4·6**, and **5·6** used in this study are available in the CCDC database under accession codes 2132382, 2132383, 2132387, 2132384, 2132385, and 2132386, respectively, here.
