# Supplementary material for: 2D conjugated microporous polyacetylenes synthesized via halogen-bond-assisted radical solid-phase polymerization for high-performance metal-ion absorbents
Source: Nat Commun. 2023 Jan 12;14:171. doi: 10.1038/s41467-023-35809-5 (PMC9837052; doi:10.1038/s41467-023-35809-5)
Supplement: Supplementary file 1 — Supplementary Information [file 41467_2023_35809_MOESM1_ESM.pdf]

Supplementary Information

2D conjugated microporous polyacetylenes synthesized *via* halogen-bond-assisted radical solid-phase polymerization for high-performance metal-ion absorbents

Hong Tho Le[1], Chen-Gang Wang[2], Atsushi Goto[1]

[1]School of Chemistry, Chemical Engineering and Biotechnology, Nanyang Technological University, 62 Nanyang Drive, 637459 Singapore (Singapore)

Hong Tho Le & Atsushi Goto

[2]Institute of Sustainability for Chemicals, Energy and Environment (ISCE2), Agency for Science Technology and Research (A*STAR), 2 Fusionopolis Way, 138634 Singapore (Singapore)

Chen-Gang Wang

Table of Contents

1.1. Materials.......... 2
1.2. Measurement.......... 3
Supplementary Note 2: Experimental Procedure.......... 8
2.1. General Procedure for Preparation of Cocrystals Using Monomers (**1−5**), XB Linkers (**6−8**), and Initiator (DMPA, **9**) via Evaporation Method.[2].......... 8
2.2. Monomer Alignments and Four Possible Monomer Addition (Propagation) Patterns.......... 8
2.3. UV Irradiation to Two-Component Cocrystal Monomers.......... 12
2.4. Photo-SPP of Three-Component Cocrystal Monomers.[2].......... 12
2.5. Removal of Linker After Polymerization.......... 13
2.6. Solution-Phase Polymerization (Comparison Experiment).......... 13
2.7. Doping PPDA With Iodine (I_2) Vapor.[3].......... 14
2.8. Exfoliation of PPDA-CMPs.......... 14
2.9. Adsorption and Desorption of Metal Ions.......... 15
2.10. Single-Crystal X-Ray Crystallography.......... 17
2.11. ^{1}H NMR Spectra after UV Irradiation to Two-Component Cocrystal Monomers.......... 23
2.12. Powder X-Ray Diffraction.......... 31
2.13. FT-IR Spectra.......... 33
2.14. TEM & SEM Images.......... 38
2.15. TGA Curves.......... 45
2.16. AFM Images.......... 46
2.17. BET Isotherms and Linear Plots.......... 49
Supplementary references.......... 61
Author Information.......... 61

Supplementary Note 1. Materials and Measurement.

1.1. Materials.

Pyridyl-3,5-diacetylene (PDA) (**1**) (or 3,5-diethynylpyridine) (>96.0%, Tokyo Chemical Industry (TCI), Japan), 3-pyridylacetylene (3PA) (**2**) (or 3-ethynyl pyridine) (98%, Sigma Aldrich, USA), 4-pyridylacetylene (4PA) (**3**) (or 4-ethynyl pyridine) (Aldrich^{CPR}, Sigma Aldrich), pyridyl-2-methyl-5-acetylene (PMA) (**4**) (or 5-ethynyl-2-methylpyridine) (Aldrich^{CPR}, Sigma Aldrich), (E)-pyridyl-4-[2-(4-pyridinyl)vinyl]-3-acetylene (PPVA) (**5**) (or (E)-3-ethynyl-4-[2-(4-pyridinyl)vinyl]pyridine) (97%, Sigma Aldrich), 1,4-diiodotetrafluorobenzene ($I-C_6F_4-I$) (**6**) (98%, Sigma-Aldrich), 1,3,5-trifluoro-2,4,6-triiodotrifluorobenzene ($C_6F_3I_3$) (**7**) (97%, Alfa Aesar, USA), 1,2-diiodotetrafluoroethane ($I-(CF_2)_2-I$) (**8**) (96%, Alfa Aesar), 2,2-dimethoxy-2-phenylacetophenone (DMPA) (**9**) (99%, Sigma-Aldrich), γ-butyrolactone (GBL) (≥99%, Sigma-Aldrich), lithium hydroxide monohydrate ($LiOH \cdot H_2O$) (99.995% trace metals basis, Sigma-Aldrich), rubidium hydroxide solution (RbOH) (50 wt% in H_2O) (99.9% trace metals basis, Sigma-Aldrich), cesium hydroxide monohydrate ($CsOH \cdot H_2O$) (99.95% trace metals basis, Sigma-Aldrich), nitric acid (HNO_3) (70%, purified by redistillation) (≥99.999% trace metals basis, Sigma-Aldrich), ammonium tetrafluoroborate (NH_4BF_4) (99.999% trace metals basis, Sigma-Aldrich), dichloromethane (99.8%, Fisher Scientific, USA), ethanol (≥99.5%, absolute, Fisher Scientific), *N*,*N*-dimethylformamide (DMF) (>99.5%, Kanto Chemical, Japan), hexane (>99%, International Scientific, Singapore), deuterated dimethyl sulfoxide (DMSO-d_6) (99.9%D, Cambridge Isotope Laboratories), lithium bromide (LiBr) (>99%, TCI), and potassium bromide (KBr) (≥99%, trace metals basis, Sigma-Aldrich) were used as received. A silicon (Si) wafer (produced by Czochralski process, thickness: 525 ± 25 μm) was purchased from Matsuzaki Seisakusho (Japan).

1.2. Measurement.

The proton nuclear magnetic resonance (^{1}H NMR) spectra were recorded at room temperature on a Bruker (Germany) BBFO400 spectrometer (400 MHz) and AV400 spectrometer (400 MHz). DMSO-d_6 was used as the NMR solvent. The residual non-deuterated solvents and tetramethylsilane (TMS) were used as the internal standards for ^{1}H NMR analysis (calibration of chemical shift).

The gel permeation chromatography (GPC) analysis was performed on a Shimadzu (Kyoto, Japan) LC-2030C Plus liquid chromatograph equipped with two Shodex LF-804 columns (300 × 8.0 mm; bead size = 6 μm; pore size = 1500 Å) and one Shodex KD-802 column (300 × 8.0 mm; bead size = 6 μm; pore size = 150 Å). The eluent was DMF (containing 10 mM of LiBr) at a flow rate of 0.34 mL/min (40 °C). Sample detection was conducted using a Shimadzu differential refractometer detector RID-20A. The column system was calibrated with standard polystyrenes.

The Fourier-transform infrared spectroscopy (FTIR) analysis was carried out on a Bruker ALPHA FTIR (Bruker, US) spectrometer. KBr was used as a matrix for FTIR.

The scanning electron microscopy (SEM) images were obtained with a JSM-7600F Schottky field emission scanning electron microscope (JOEL, Japan) operated at 5 kV.

The cocrystal monomers were moulded using 2T Mini-Pellet Press (Specac, UK) to form sheets with a diameter of 7 mm. The polymers for the resistivity measurements were pressed into thin films with a diameter of 13 mm using 15T Manual Hydraulic Press (Specac).

The UV light source was a UV-LED light (365 (± 10) nm wavelength and 900 mW/cm^2 intensity) (C11924-101 and C14052-0-A5 models (Hamamatsu Photonics, Japan)).

The atomic force microscopy (AFM) images were obtained with a MultiMode Scanning Probe Microscope (Bruker) in the ScanAsystTM mode using a cantilever (ScanAsyst-Air, Bruker).

The transmission electron microscopy (TEM) images were obtained with a JEOL (Tokyo, Japan) TEM-1400 transmission electron microscope operated at 100 kV. The TEM grid was carbon-coated on 200 mesh (copper (Cu)) (Ted Pella, Redding, US).

The thermal analyses of the polymers were performed on a thermal gravimetric analysis (TGA) Q500 model device (TA instrument, New Castle, US). The TGA analysis was carried out in platinum pans under flowing air at a flow rate of 60 mL/min with a heating rate of 10 °C/min for PPDA-CMP-1 (Supplementary Fig. 31) or 20 °C/min for PPDA-CMP-2 and PPDA-CMP-3 (Supplementary Figs. 32 and 33) and heated up to 780–790 °C.

Single crystal X-ray diffraction frames were analysed with a Bruker D8 QUEST (Bruker) and integrated with the Bruker SAINT software package using a narrow-frame algorithm. The data were corrected for absorption effects using the Multi-Scan method (SADABS). The structures were solved by XT VERSION 2014/5 and refined by SHELXL-2017/1 (Sheldrick, 2017) programs, respectively. The refinement was carried out by full-matrix least-squares on F^2. Hydrogen atoms were placed using standard geometric models and with their thermal parameters riding on those of their parent atoms.

The powder X-ray diffraction (PXRD) analysis was carried out on a Bruker D8 ADVANCE (Bruker) from 10.000° to 79.994° (step size 0.020) using CuK_α radiation (wavelength (λ) = 1.541874 Å). The parameters (2 theta (2θ) and full width of half maximum (FWHM) shown in Supplementary Table 3) were calculated from PXRD spectra and data calculated from Origin software. The crystallite size was calculated using the modified Scherrer equation (least square).[1]

The layers distance/plane spacing (d_{hkl}), microstrain (ε), crystallite size (D), and dislocation density (δ) were calculated from the following formula:

Scherrer equation: $\ln\beta = \ln\frac{1}{\cos\theta} + \ln\frac{K\lambda}{D} = \ln\frac{1}{\cos\theta} + \text{intercept}\ b$ (1)

Crystallite size $(D) = \frac{K\lambda}{e^{\text{intercept}\ b}}$ [nm] (2)

Layer spacing $(d_{\text{hkl}}) = \frac{n\lambda}{2\sin\theta}$ [nm] (3)

Microstrain $(\varepsilon) = \frac{\text{radians(FWHM)}}{4\tan\theta}$ (4)

Dislocation density $(\delta) = \frac{1}{D^2}$ [nm^{-2}] (5)

where:

β is the radians of FWHM;

θ is the incident angle (the angle between the incident ray and the scatter plane) [°];

K is the Scherrer constant, which is a dimensionless shape factor ($K = 0.9$);

λ is the radiation wavelength ($\lambda = 0.1541874$ nm);

D is the crystallite size [nm] in the powder sample and was obtained from the intercept of the plot of $\ln\beta$ vs $\ln(1/\cos\theta)$ according to equation (1) (or (2));

n is an integer ($n = 1$).

The surface resistivity (ρ_s (Ω/sq)) values of the PPDA-CMP-1 and PPDA synthesized in solution polymerization were determined by a four-point technique with a Loresta-GP resistivity meter (Mitsubishi Chemical Analytech (Japan), MCP-T610) at room temperature. The polymers are pressed to form thin films using a manual hydraulic press (15T) prior to the analyses. The Loresta-

GP MCP-T610 meter included a standard accessories PSP probe (MCP-TP06P, 4-pins, inter-pin distance 1.5 mm, pin points 0.26R, spring pressure 70 g/pin) and a probe checker (MPC-TRPS). The thicknesses (L (cm)) of the polymer films were measured by a high-precision digital caliper (Fowler ProMax-Cal, Japan). The electrical conductivity (σ (S cm^{-1})) values were calculated according to equation (6):

$$\sigma = \frac{1}{\rho_s L}\ [\mathrm{S\ cm^{-1}}] \tag{6}$$

The surface areas of PPDA-CMP-1, PPDA-CMP-2, and PPDA-CMP-3 were analyzed with a Micromeritics 3FLEX (Micromeritics, USA) analyzer at –196 °C. Before measurement, the samples were degassed totally in the nitrogen (N_2) atmosphere at 120 °C for 24 h and then backfilled with N_2. We studied the relative pressure (P/P_0) from 0 to 1 at –196 °C, where P_0 is the saturated pressure of adsorbent (N_2). The specific surface areas (m^2 g^{-1}) were determined via Brunauer-Emmett-Teller (BET) model at the linearized P/P_0 range from 0.06 to 0.14 according to equation (7):

$$\frac{1}{Q((P_0/P)-1)} = \frac{1}{Q_\mathrm{m} c_\mathrm{BET}} + \frac{(c_\mathrm{BET}-1)}{Q_\mathrm{m} c_\mathrm{BET}}\left(\frac{P}{P_0}\right) \tag{7}$$

where Q is the volume of nitrogen gas adsorbed per weight of adsorbent (cm^3 g^{-1} STP) at a given relative pressure (P/P_0), Q_m is the volume of nitrogen gas adsorbed to form the monolayer per weight of adsorbent (cm^3 g^{-1} STP), and c_BET is the BET constant (STP is standard condition at temperature 273 K and pressure 1 atm). The plot of $1/(Q((P_0/P)–1))$ vs P/P_0 was linear for all studied cases (Supplementary Fig. 37), which indicates the formation of the monolayer in this range (P/P_0 = 0.06–0.14). From the slope and intercept of the plot, the c_BET and Q_m values were determined. The BET specific surface area (S_BET, cm^2 g^{-1}) was calculated from Q_m according to equation (8):

$$S_{\mathrm{BET}} = \frac{Q_{\mathrm{m}} N_{\mathrm{A}} A_{\mathrm{m}}}{M_{\mathrm{v}}} \quad (8)$$

where the N_A is Avogadro's number (6.022×10^{23} mol^{-1}), A_m is the molecular cross-sectional area for liquid N_2 (0.162 nm^2), and M_v is the molar volume for the ideal gas at STP (22414 cm^3 mol^{-1}).

The average pore diameter (d_{BET} (nm)) was calculated at $P/P_o = 0.99$, assuming a cylindrical pore. At $P/P_o = 0.99$, the pores were assumed to be completely filled with N_2 and the total volume (V_{BET}) of the adsorbed N_2 can be considered as the total pore volume. The d_{BET} (nm) was calculated according to equation (9):

$$d_{\mathrm{BET}} = \frac{4V_{\mathrm{BET}}}{S_{\mathrm{BET}}} \quad (9)$$

where V_{BET} is the Q at $P/P_o = 0.99$.

An inductively coupled plasma optical emission spectrometer (ICP-OES) (ICAP 6500, Thermo Scientific, US) was used to determine the metal ion concentrations (ppm or mg L^{-1}) of the samples. The simultaneous axial and radial view of the plasma was enabled by a synchronous vertical dual view (SVDV). The analytical conditions are: radio frequency (RF) power 1150 W, nebulizer gas flow 0.08 L min^{-1}, auxiliary gas flow 1.0 L min^{-1}, plasma gas flow 12.0 L min^{-1}, and signal accusation time 3 s/replicate for 3 replicates. All standards and samples were dissolved (diluted) in nitric acid (2 wt% in ultrapure water) before analysis. Metal ions Li, Rb, and B were analysed individually at wavelength λ = 670.784, 780.023, and 249.773 nm, respectively, with the correlation factor of the calibration curve $R^2 > 0.999$. For all the elements determined, the uncertainty of the analytical concentration (RSD) was <5%.

Supplementary Note 2: Experimental Procedure.

2.1. General Procedure for Preparation of Cocrystals Using Monomers (1−5), XB Linkers (6−8), and Initiator (DMPA, 9) via Evaporation Method.[2]

Monomers **1−5,** XB linkers **6−8 (**N/I molar ratio = 1/1), and initiator (DMPA, **9**) were dissolved in dichloromethane in a flask. For example, monomer **1** (0.10 g, 0.79 mmol), XB linker **6** (0.16 g, 0.39 mmol) (N/I molar ratio = 1/1), and DMPA (67.0 mg, 0.26 mmol) were dissolved in dichloromethane (2 mL) in a flask. Evaporation of dichloromethane was performed using a rotary evaporator (Hei-VAP Precision, Heidolph, Germany) under constant pressure (200 mbar) and rotation speed (10 rpm), yielding cocrystals after complete evaporation of dichloromethane.

Dichloromethane would initially evaporate relatively quickly. Once the solution has been saturated, cocrystals began to form. Because the solution was not pure dichloromethane but a mixture of monomer, linker, photo-initiator, and solvent, the evaporation gradually slowed down and needed a reduced pressure to completely remove the solvent. The low rotation speed (10 rpm) would prevent vibrations to the solution and was applied not to disturb the growth of the cocrystals.

2.2. Monomer Alignments and Four Possible Monomer Addition (Propagation) Patterns.

Supplementary Table 1 shows four possible monomer addition (propagation) patterns, i.e., path A (parallel alignment + head-to-head and tail-to-tail propagation), path B (parallel alignment + head-to-tail propagation), path C (zigzag alignment + head-to-head and tail-to-tail propagation), and path D (zigzag alignment + head-to-tail propagation). Supplementary Table 1 shows single-crystal X-ray crystallography data. The π-π distance between two linkers, hence the distance of two pyridyl (R) groups of monomers, was 3.571–7.391 Å. The propagation in the parallel alignment (paths A and B) will give R-R distances of 1.54–2.49 Å in the generated polymers,

which are much shorter than the original R-R distances (3.571–7.391 Å) (hence π-π distances) in the monomer cocrystals and will cause significant deformation of the crystal structures. Therefore, paths A and B might occur but would not be major paths in the present polymerizations. The propagation in the zigzag alignment (paths C and D) will give parallel (every other) R-R distances of 4.42–4.98 Å in the generated polymers, which are close to the original parallel R-R distances (3.571–7.391 Å) (hence π-π distances) in the monomer cocrystals and will suppress the deformation of the crystal structures. Therefore, paths C and D would be more likely to occur than paths A and B. Electronically and sterically, path D (head-to-tail propagation) would be more favorable than path C (head-to-head and tail-to-tail propagation). This is because, in path D, the electron-rich and sterically hindered propagating radical carbon (with an electro-donating R group) can react with an acetylene monomer at the electron-deficient and sterically less hindered tail carbon (C–H) rather than the electron-rich and sterically more hindered head carbon (C–R). Thus, path D would be favorable. However, other paths (A–C) might also occur to some extents.

For the di-acetylene monomer (**1**), path D can occur in two ways because two acetylenes are present in one monomer (Supplementary Fig. 1). In one way, monomers are linked in a face-to-face manner, where two monomers are bridged via two bonds, forming an intra-ladder (single ladder) polymer structure (structure D1 in Supplementary Fig. 1). In another way, monomers are linked in a staggered manner, where one monomer is linked with one monomer via one bond and another monomer via another bond, forming an inter-ladder polymer nanosheet structure (structure D2 in Supplementary Fig. 1). These two ways might also operate in mixed manners, forming mixed intra-inter-ladder polymer nanosheet structures; an example is structure D3 in Supplementary Fig. 1. Experimentally, we observed polymer nanosheets (as described in the manuscript), and hence the structures would not be a pure form of structure D1 but be structure D2 and mixed structures

exemplified by structure D3. We put structure D2 in Fig. 1d as a guide. It should be noted that other structures might also be formed. Different propagation patterns (paths A–D) and formation of mixed structures exemplified by structure D3 might occur simultaneously. Also, there might be defects in the monomer cocrystal structures. Thus, structure D2 is viewed as one of the probable structures (Supplementary Fig. 1).

In our previous solid phase polymerization (SPP) of vinyl monomers,[2] tacticity (stereo structure) was not regulated because the terminal C–C$^\bullet$ bond can rotate even in the limited freedom in the cocrystal. Meanwhile, monomer addition patterns (head/tail configuration) would mostly be determined by the alignment of monomers, because monomer re-alignment (entire molecular rotation) in the cocrystals would hardly occur due to the limited freedom. Polyacetylenes consist of sp^2 carbons in the backbones and hence are rigid. Therefore, mobility of the chain end radical is restricted, which would also assist the retention of monomer alignment structures in the polymer structures.

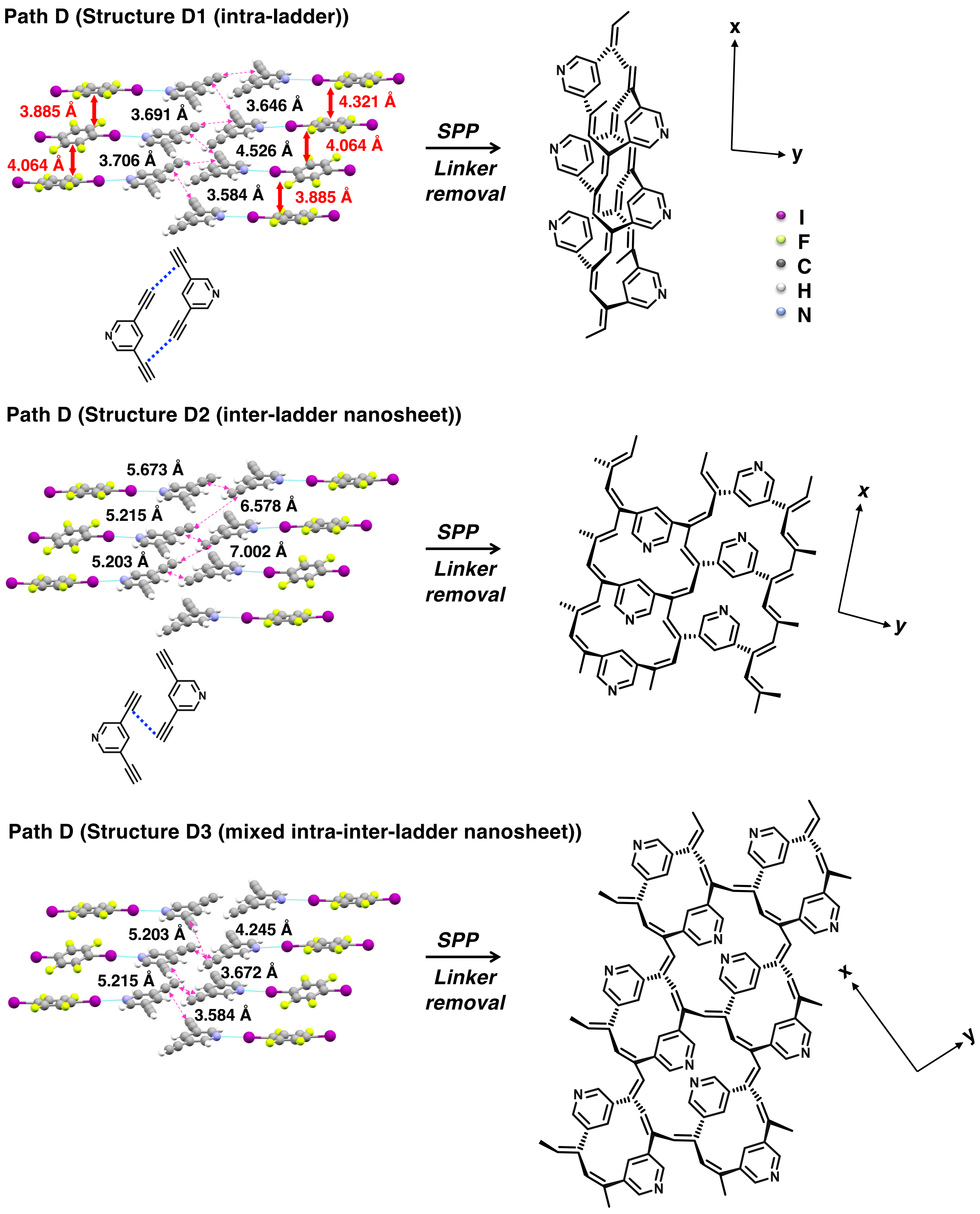

Supplementary Fig. 1: Three possible structures of PPDA-CMP-1 expected from the monomer cocrystal structure **1**·**6**.

2.3. UV Irradiation to Two-Component Cocrystal Monomers.

The two-component cocrystal monomers of monomers **1−5** and XB linkers **6−8** were prepared with vaporization method as described (without photo-initiator DMPA). The obtained cocrystal monomers were put in a vial. The vial was capped with a rubber septum, and oxygen was removed with an argon flow for 10 min. The cocrystal monomers were then irradiated with UV light (λ = 365 nm) at room temperature for 40 h. The irradiated samples were then analysed with ^{1}H NMR (Supplementary Figs. 8−15) and GPC, showing no formation of polymers for all studied cases.

2.4. Photo-SPP of Three-Component Cocrystal Monomers.[2]

The cocrystal solid powder obtained above was moulded using a 2T mini-hand hydraulic press to form a round-shape sheet with a diameter of 7 mm. The sheet was put in a vial. The vial was capped with a rubber septum, and oxygen was removed with an argon flow for 10 min. The sheet was irradiated with UV light (λ = 365 nm) at room temperature for 40 h. DMPA gradually decomposed to continuously supply radicals under 365 nm UV LED during the polymerization rather than to generate radicals in a bursting manner. In the present study, DMPA was sufficient to attain nearly quantitative monomer conversions. (We also studied the SPP of **1·6** at varied polymerization time (0.25–40 h) and with varied amounts of photo-initiator (0.01–0.67 equiv to linker and 0.005–0.335 equiv to the monomer) (Supplementary Table 2). The polymers formed at shorter times (0.25, 3, 7, and 24 h) and smaller amounts of photo-initiator (0.01 and 0.10 equiv to linker (0.005 and 0.05 equiv to monomer, respectively)) did not reach high monomer conversions or retain the grain (cocrystal) structures, suggesting that the SPP for 40 h with 0.67 equiv of DMPA to linker (0.335 equiv to monomer) is an optimal SPP condition.) The sheet was washed with ethanol (20 mL) three times to remove soluble polymer, residual monomer (if present), XB linker,

and residual DMPA, yielding purified insoluble polymer. The soluble part of the polymer in ethanol (10 mL) was reprecipitated into hexane (100 mL) to remove the residual monomer, XB linker, and DMPA, and analysed with GPC to determine the molecular weight and dispersity of the soluble part of polymer. The monomer conversion was determined by analyzing the ethanol solution (containing soluble polymer and monomer) using ^{1}H NMR and the weight of insoluble polymer (monomer conversion = (total amount of soluble polymer and insoluble polymer)/(total amount of monomer, soluble polymer, and insoluble polymer)). The monomer cocrystals **1·6**, **1·7**, and **1·8** and their polymers obtained via SPP were analysed with TEM. The dried solid samples were grinded into fine powders and directly attached onto the Cu grids (Supplementary Figs. 23–25).

2.5. Removal of Linker After Polymerization.

After the SPP, the polymerized **1·6**, **1·7**, **1·8**, **2·6**, and **2·7** cocrystals were purified (washed) using ethanol to remove the linkers. The FTIR analysis of the washed polymers showed no C–F and C–I peaks of the linkers, demonstrating complete removal of the linkers from the polymers (Supplementary Figs. 18–22).

2.6. Solution-Phase Polymerization (Comparison Experiment).

In a typical run, monomer **1** (0.10 g, 0.79 mmol) and DMPA (67.0 mg, 0.26 mmol) were dissolved in dichloromethane in a vial, in which XB linker was not added. The solution formed a thin liquid layer at the bottom of the vial. The vial was capped with a rubber septum, and oxygen was removed with an argon flow for 10 min. The solution was irradiated with UV light (λ = 365 nm) at room temperature for 40 h, yielding polymer. The solution was dropped in ethanol (10 mL) to precipitate polymer (insoluble polymer in ethanol) for separating out from soluble polymer, residual monomer, and residual DMPA. The insoluble polymer was separated by centrifugation.

The soluble part of the polymer in ethanol (10 mL) was reprecipitated into hexane (100 mL) to remove the residual monomer and DMPA, and analysed with GPC to determine the molecular weight and dispersity of the soluble part of the polymer. The monomer conversion was determined by analyzing the ethanol solution (containing soluble polymer and monomer) using ^{1}H NMR and the weight of insoluble polymer (monomer conversion = (total amount of soluble polymer and insoluble polymer)/(total amount of monomer, soluble polymer, and insoluble polymer)).

2.7. Doping PPDA With Iodine (I_2) Vapor.[3]

In a typical run, 18 g iodine powder was loaded into a 20 mL capped glass vial and allowed for reaching solid-vapor equilibration of the iodine inside the vial at 100 °C for 10 min. In parallel, the PPDA-CMP-1 polymer film was heated in a separate vial at 100 °C for 10 min. The polymer film was subsequently placed into the iodine vial, which was capped tightly and heated at 100 °C for 30 mins. The film of the PPDA obtained in the solution-phase polymerization was studied similarly.

2.8. Exfoliation of PPDA-CMPs.

PPDA-CMP-1 (4 mg) was dispersed in 4 mL of γ-butyrolactone (GBL) and sonicated for 30 mins to obtain a 0.1 wt% dispersed solution. A part of the solution was further diluted 500 times in GBL to obtain a 2×10^{-4} wt% dispersed solution. The two solutions (0.1 and 2×10^{-4} wt% solutions) were heated at 50 °C for 5 days with gentle stirring to induce exfoliation.[4] Subsequently, the dispersed solution (1 μL) was dropped on Cu grids and cleaned Si wafers and dried under vacuum for TEM and AFM analysis, respectively. PPDA-CMP-2 and PPDA-CMP-3 were exfoliated similarly. The 0.1 wt% solutions were used for the AFM analysis of exfoliated PPDA-CMP-1 for Fig. 2d (right) and Supplementary Fig. 34. The 2×10^{-4} wt% solutions were used for all

other TEM and AFM analyses of exfoliated PPDA-CMP-1, PPDA-CMP-2, and PPDA-CMP-3 for Figs. 2c and 2d (left) and Supplementary Figs. 26, 27b, 28b, 35 and 36.

2.9. Adsorption and Desorption of Metal Ions.

PPDA-CMP-1, PPDA-CMP-2, and PPDA-CMP-3 were placed in an oven at 120 °C overnight prior to adsorption. For the Li^+ adsorption (0.1 wt% of Li^+), $LiOH·H_2O$ (0.302 g, 7.20 mmol) was dissolved in 50 mL of H_2O. Similar preparations were carried out for the mixed ion solution (Li^+ + Rb^+ + Cs^+ at 0.1 wt% for each) ($LiOH·H_2O$ (0.302 g, 7.20 mmol), RbOH (50 wt% in water) (0.1199 g, 0.585 mmol), and $CsOH·H_2O$ (0.063 g, 0.376 mmol)) and the B^{3+} solution (0.1 wt% of B^{3+}) (NH_4BF_4 (0.485 g, 4.625 mmol)). PPDA-CMP (12.5 mg) was subsequently added to each solution. The mixture was sonicated four times for 1 h in total (15 mins each time) at a 600W ultrasonication power and then left overnight for 24 h at room temperature.

The ion-adsorbed PPDA-CMPs were rinsed with water three times to fully remove ions possibly covering the surface of the CMP powder and dried under vacuum for 24 h to obtain the ion-absorbed PPDA-CMP-1, PPDA-CMP-2, and PPDA-CMP-3. The solution parts before and after adsorption were filtered, diluted 500 times (with HNO_3 2 wt% in ultrapure water), and analysed with ICP-OES to determine the ion concentrations in the solutions before and after adsorption (Tables 2 and 3 and Supplementary Tables 6 and 7). The content of metal ion (wt%) was calculated according to equation (10):

$$\mathrm{wt\%} = (C_0 - C) \times \frac{V_{\mathrm{sol}}}{m_{(\mathrm{PPDA-CMP})}} \times 100\% \quad (10)$$

where:

C_0 is the concentration of metal ion before adsorption [ppm or mg L^{-1}];

C is the concentration of metal ion after adsorption [ppm or mg L^{-1}];

V_{sol} is the volume of the metal ion solution [L];

$m_{(PPDA\text{-}CMP)}$ is the mass of PPDA-CMP [mg].

The rinsed water was also analysed using ICP-OES, showing the amount of the ions covering the CMP surface was negligible (below the analytical detection limit) compared with the amount of ions adsorbed inside the CMP in all cases.

2.10. Single-Crystal X-Ray Crystallography.

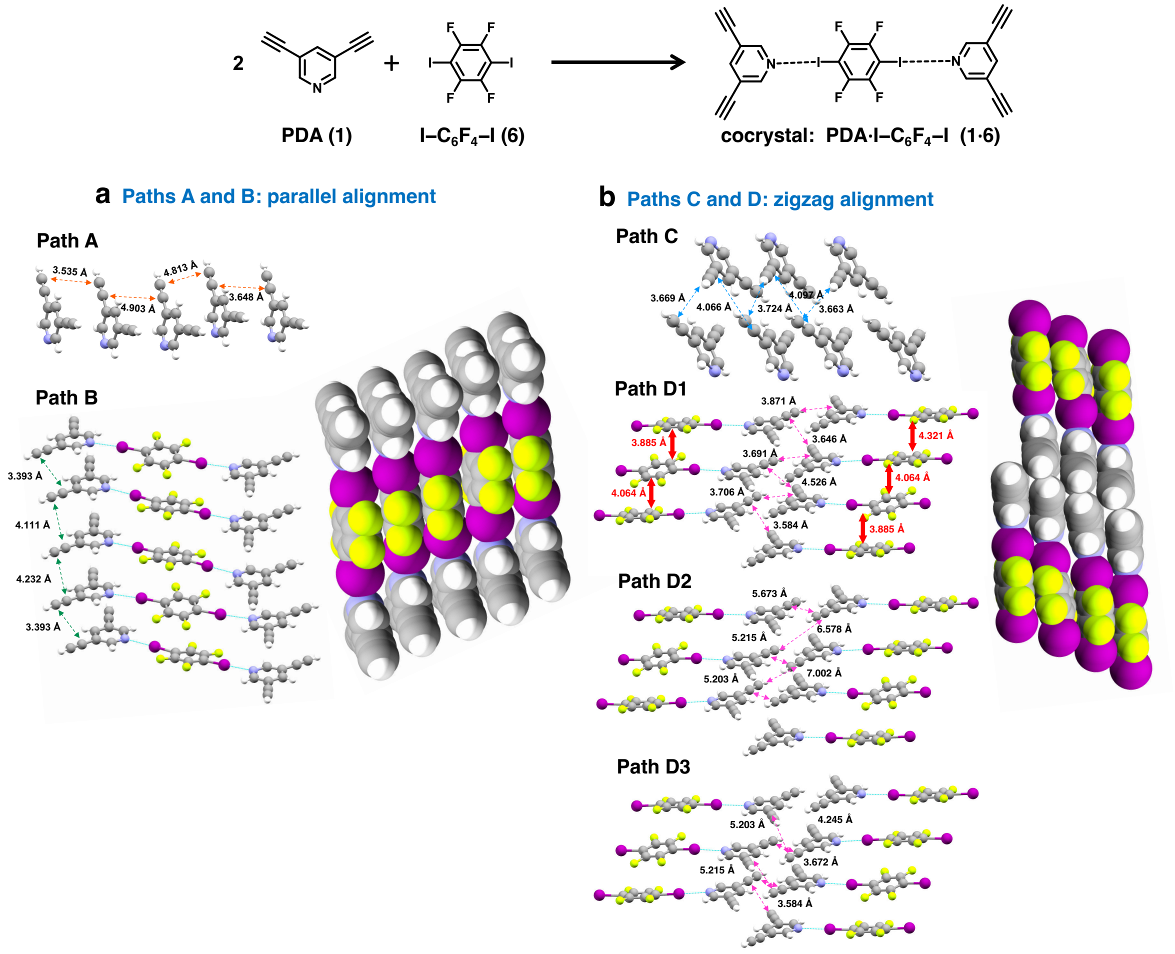

Supplementary Fig. 2: Single-crystal X-ray crystallography structure of monomer cocrystal PDA·I−C_6F_4−I (1·6) and four possible monomer addition (propagation) patterns (Supplementary Table 1, entry 1). (**a**) Paths A (orange arrows) and B (green arrows) in parallel alignments with monomer distances of 3.535−4.813 Å (tail-to-tail) and 3.648−4.903 Å (head-to-head) (path A) and 3.393−4.232 Å (head-to-tail) (path B). (**b**) Paths C (blue arrows) and D (pink arrows) in zigzag alignments with monomer distances of 3.663−3.724 Å (tail-to-tail) and 4.066−4.097 Å (head-to-head) (path C), and 3.584–7.002 Å (head-to-tail) (path D). The π–π distance between two linkers was 3.885–4.321 Å. The molecular packing views down crystallographic b axis in ball-and-stick and space-filling representation are constructed using the crystallographic information file and software package Mercury 3.10.3. Carbon: gray; hydrogen: white; fluorine: yellow; nitrogen: blue; iodine: magenta. Halogen bonds are presented as light blue dotted lines.

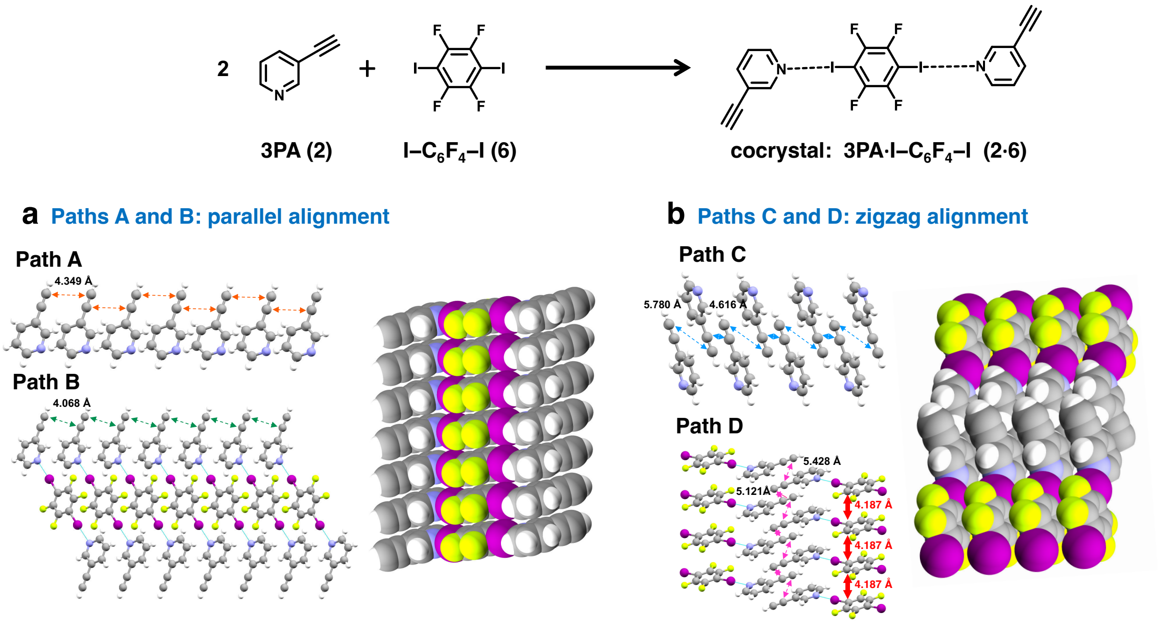

Supplementary Fig. 3: Single-crystal X-ray crystallography structure of monomer cocrystal 3PA·I–C_6F_4–I (2·6) and four possible monomer addition (propagation) patterns (Supplementary Table 1, entry 2). (**a**) Paths A (orange arrows) and B (green arrows) in parallel alignments with monomer distances of 4.349 Å (head-to-head and tail-to-tail) (path A), and 4.068 Å (head-to-tail) (path B). (**b**) Paths C (blue arrows) and D (pink arrows) in zigzag alignments with monomer distances of 4.616 Å (head-to-head) and 5.780 Å (tail-to-tail) (path C), and 5.121–5.428 Å (head-to-tail) (path D). The π–π distance between two linkers was 4.187 Å.

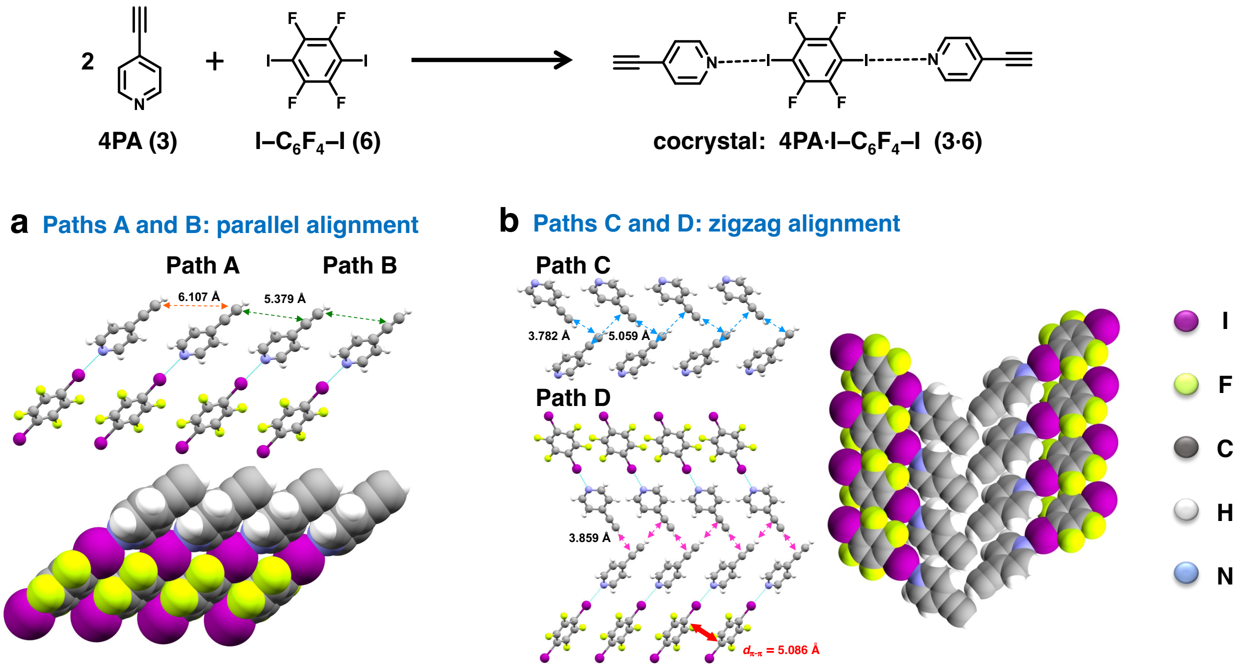

Supplementary Fig. 4: Single-crystal X-ray crystallography structure of monomer cocrystal 4PA·I−C_6F_4−I (3·6) and four possible monomer addition (propagation) patterns (Supplementary Table 1, entry 3). (**a**) Paths A (orange arrows) and B (green arrows) in parallel alignments with monomer distances of 6.107 Å (head-to-head and tail-to-tail) (path A), and 5.379 Å (head-to-tail) (path B). (**b**) Paths C (blue arrows) and D (pink arrows) in zigzag alignments with monomer distances of 3.782 Å (tail-to-tail) and 5.059 Å (head-to-head) (path C), and 3.859 Å (head-to-tail) (path D). The π–π distance between two linkers was 5.086 Å.

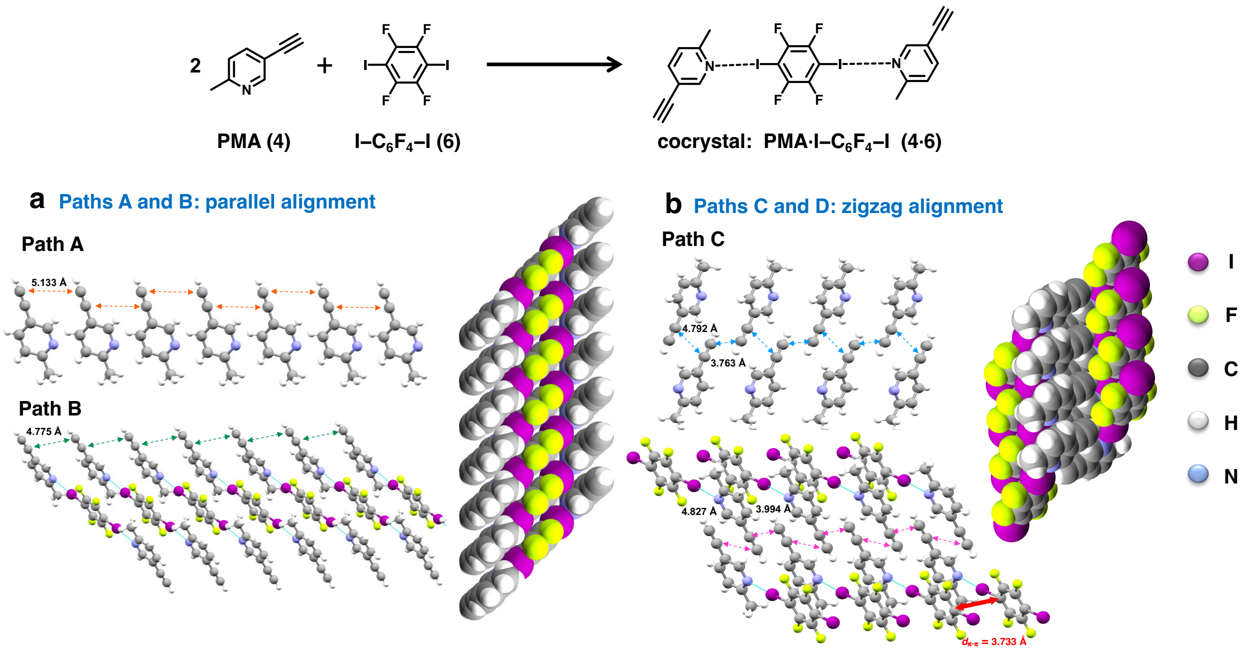

Supplementary Fig. 5: Single-crystal X-ray crystallography structure of monomer cocrystal PMA·I–C_6F_4–I (4·6) and four possible monomer addition (propagation) patterns (Supplementary Table 1, entry 4). (**a**) Paths A (orange arrows) and B (green arrows) in parallel alignments with monomer distances of 5.133 Å (head-to-head and tail-to-tail) (path A), and 4.775 Å (head-to-tail) (path B). (**b**) Paths C (blue arrows) and D (pink arrows) in zigzag alignments with monomer distances of 3.763 Å (tail-to-tail) and 4.792 Å (head-to-head) (path C), and 3.994–4.827 Å (head-to-tail) (path D). The π–π distance between two linkers was 3.733 Å.

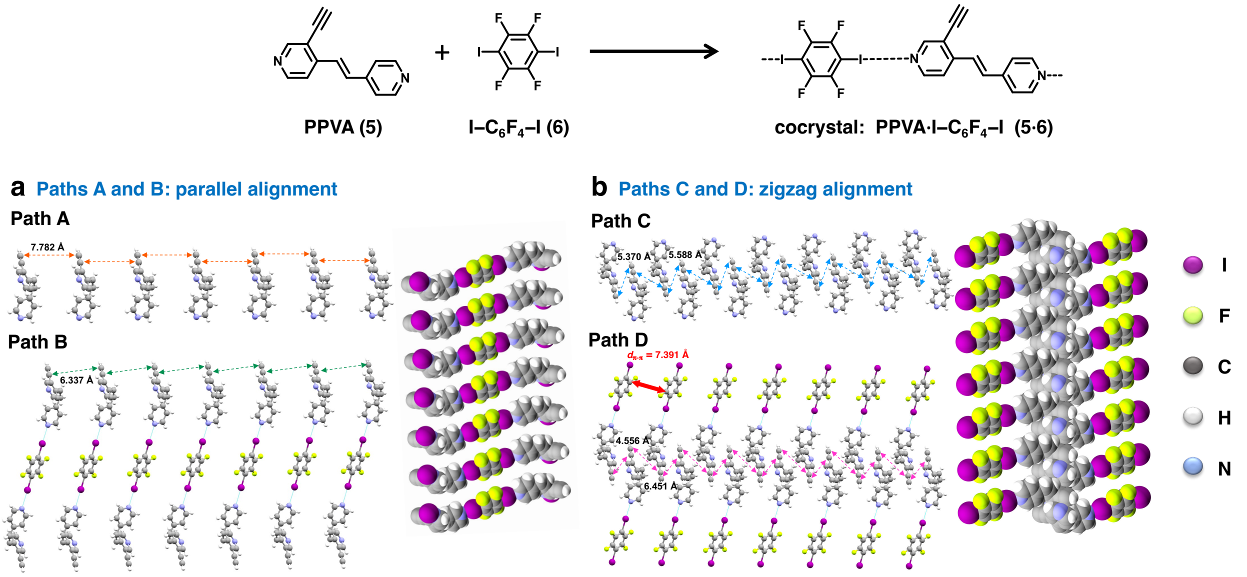

Supplementary Fig. 6: Single-crystal X-ray crystallography structure of monomer cocrystal PPVA·I–C_6F_4–I (5·6) and four possible monomer addition (propagation) patterns (Supplementary Table 1, entry 5). (**a**) Paths A (orange arrows) and B (green arrows) in parallel alignments with monomer distances of 7.782 Å (head-to-head and tail-to-tail) (path A), and 6.337 Å (head-to-tail) (path B). (**b**) Paths C (blue arrows) and D (pink arrows) in zigzag alignments with monomer distances of 5.370 Å (tail-to-tail) and 5.588 Å (head-to-head) (path C), and 4.556–6.451 Å (head-to-tail) (path D). The π–π distance between two linkers was 7.391 Å.

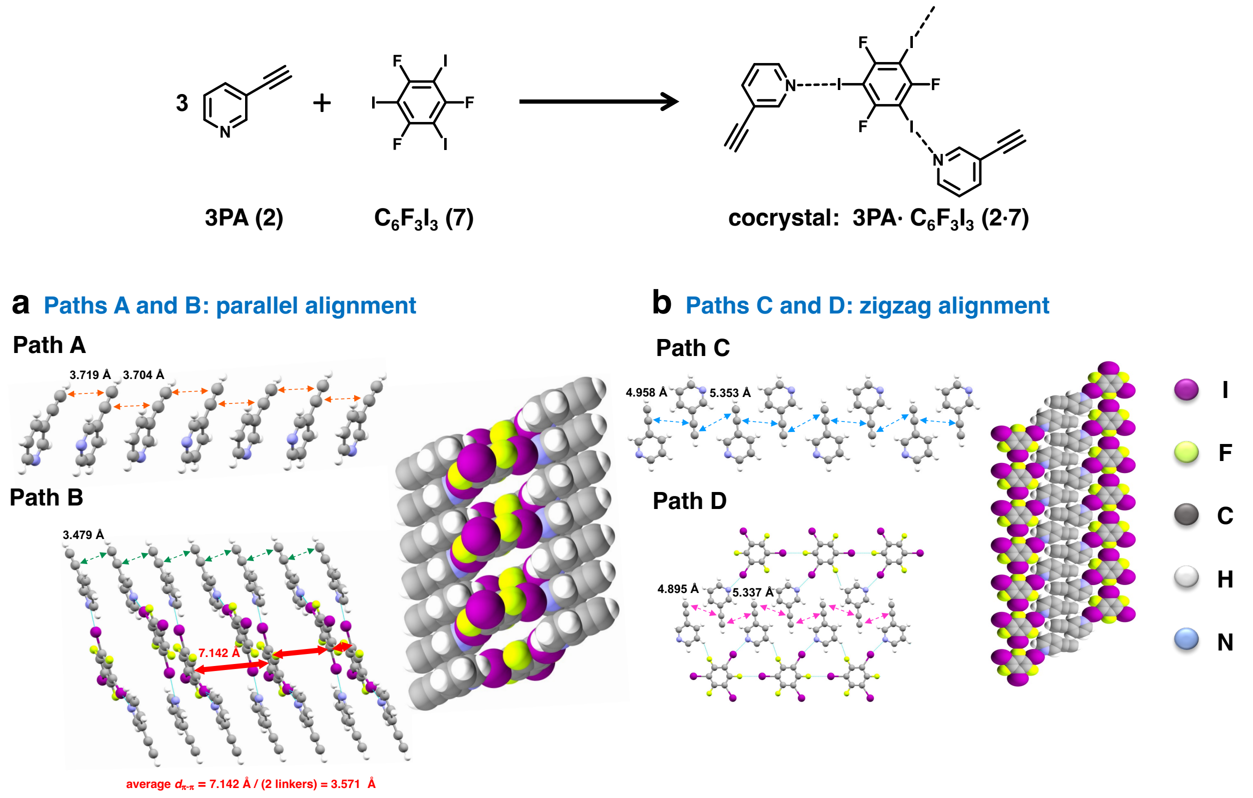

Supplementary Fig. 7: Single-crystal X-ray crystallography structure of monomer cocrystal $3PA\cdot C_6F_3I_3$ (2·7) and four possible monomer addition (propagation) patterns (Supplementary Table 1, entry 6). (**a**) Paths A (orange arrows) and B (green arrows) in parallel alignments with monomer distances of 3.704 Å (head-to-head) and 3.719 Å (tail-to-tail) (path A) and 3.479 Å (head-to-tail) (path B). (**b**) Paths C (blue arrows) and D (pink arrows) in zigzag alignments with monomer distances of 4.958 Å (head-to-head) and 5.353 Å (tail-to-tail) (path C), and 4.895−5.337 Å (head-to-tail) (path D). The average π–π distance between two linkers was 3.571 Å.

2.11. ¹H NMR Spectra after UV Irradiation to Two-Component Cocrystal Monomers.

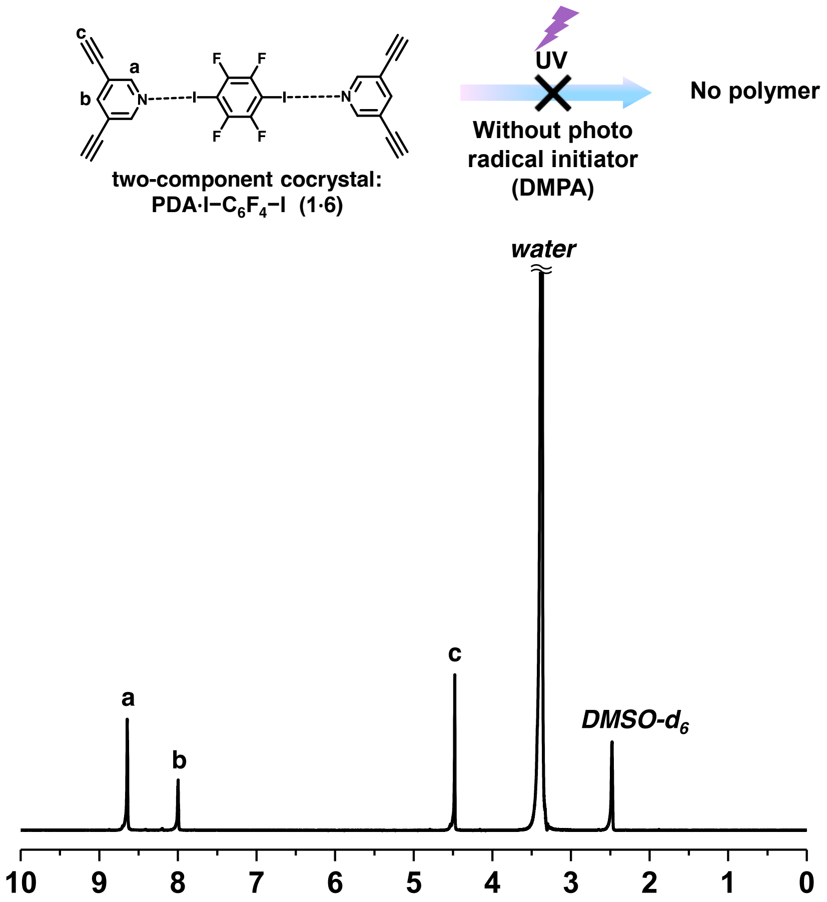

Supplementary Fig. 8: ^{1}H NMR spectrum of two-component monomer cocrystal **1·6** (without photo-initiator) after UV irradiation at $\lambda = 365$ nm for 40 h (400 MHz, 298 K, DMSO-d_6).

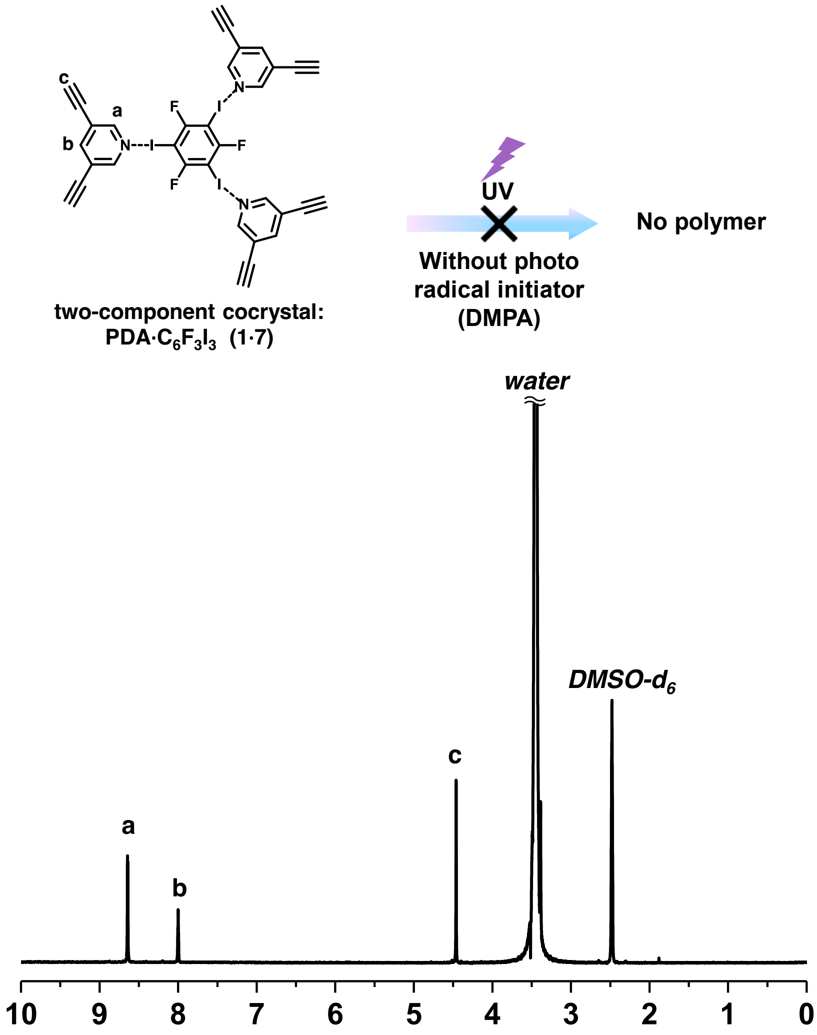

Supplementary Fig. 9: ^{1}H NMR spectrum of two-component monomer cocrystal **1·7** (without photo-initiator) after UV irradiation at λ = 365 nm for 40 h (400 MHz, 298 K, DMSO-d_6).

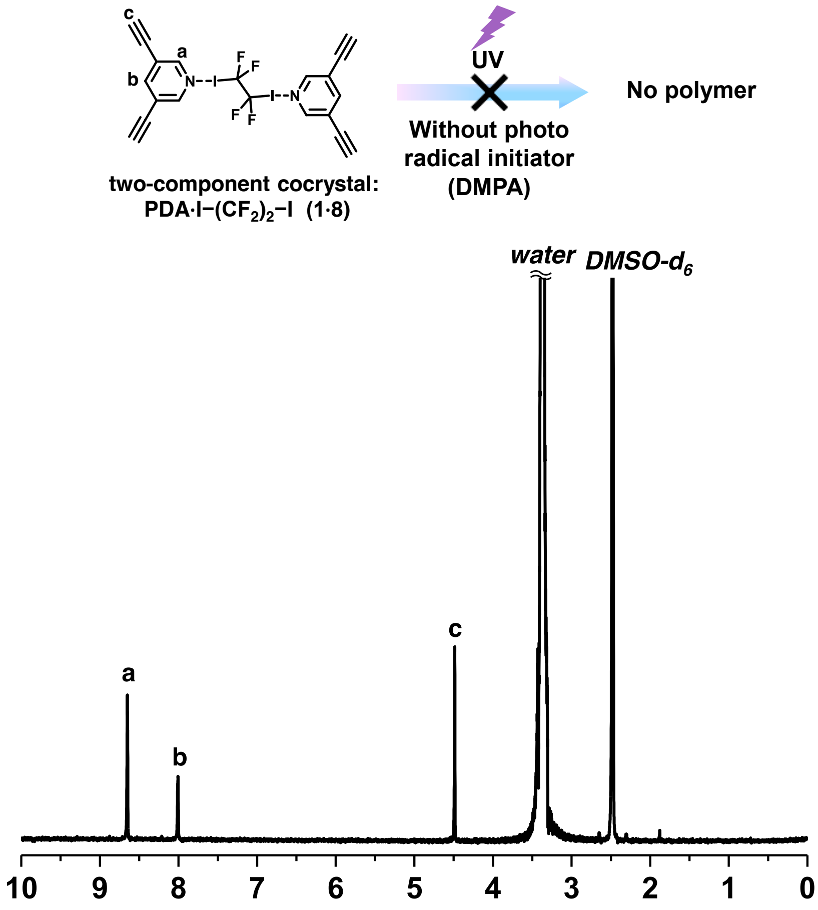

Supplementary Fig. 10: ^{1}H NMR spectrum of two-component monomer cocrystal **1·8** (without photo-initiator) after UV irradiation at λ = 365 nm for 40 h (400 MHz, 298 K, DMSO-d_6).

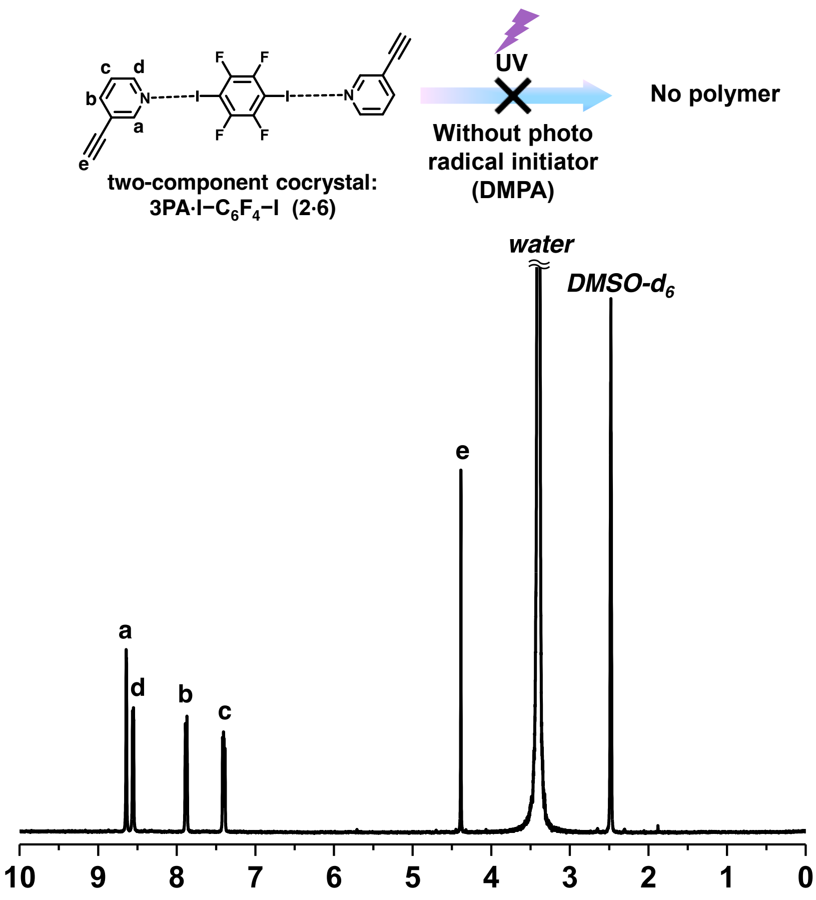

Supplementary Fig. 11: ^{1}H NMR spectrum of two-component monomer cocrystal **2·6** (without photo-initiator) after UV irradiation at λ = 365 nm for 40 h (400 MHz, 298 K, DMSO-d_6).

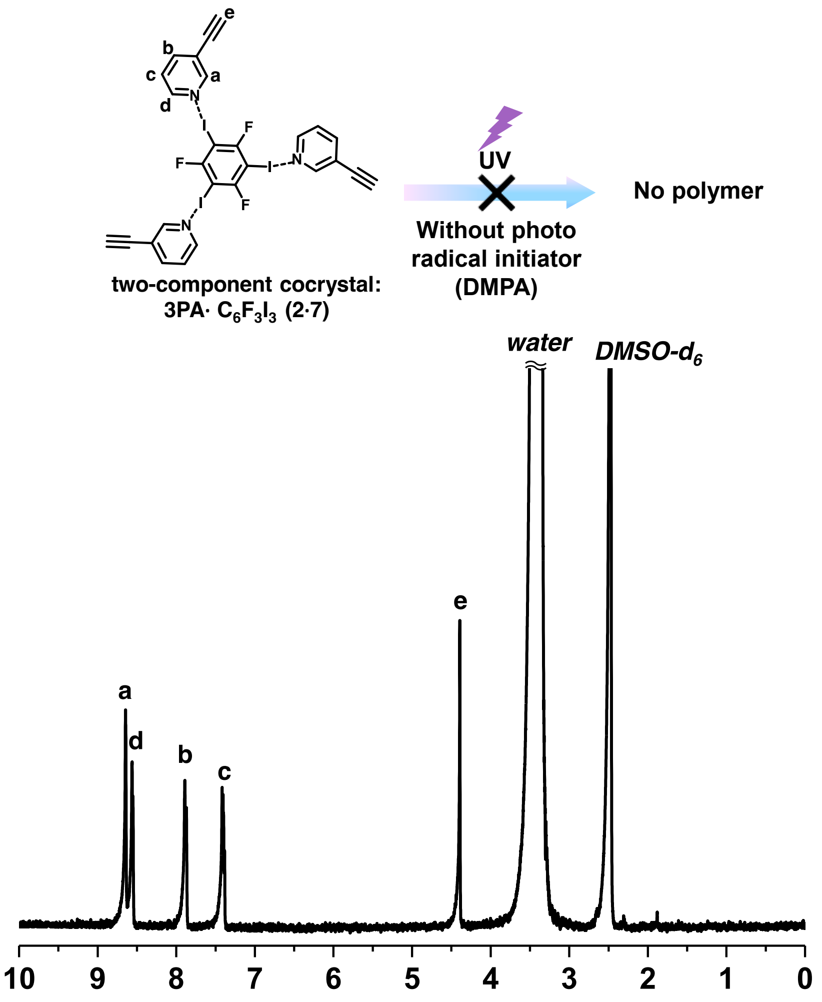

Supplementary Fig. 12: ^{1}H NMR spectrum of two-component monomer cocrystal **2·7** (without photo-initiator) after UV irradiation at $\lambda = 365$ nm for 40 h (400 MHz, 298 K, DMSO-d_6).

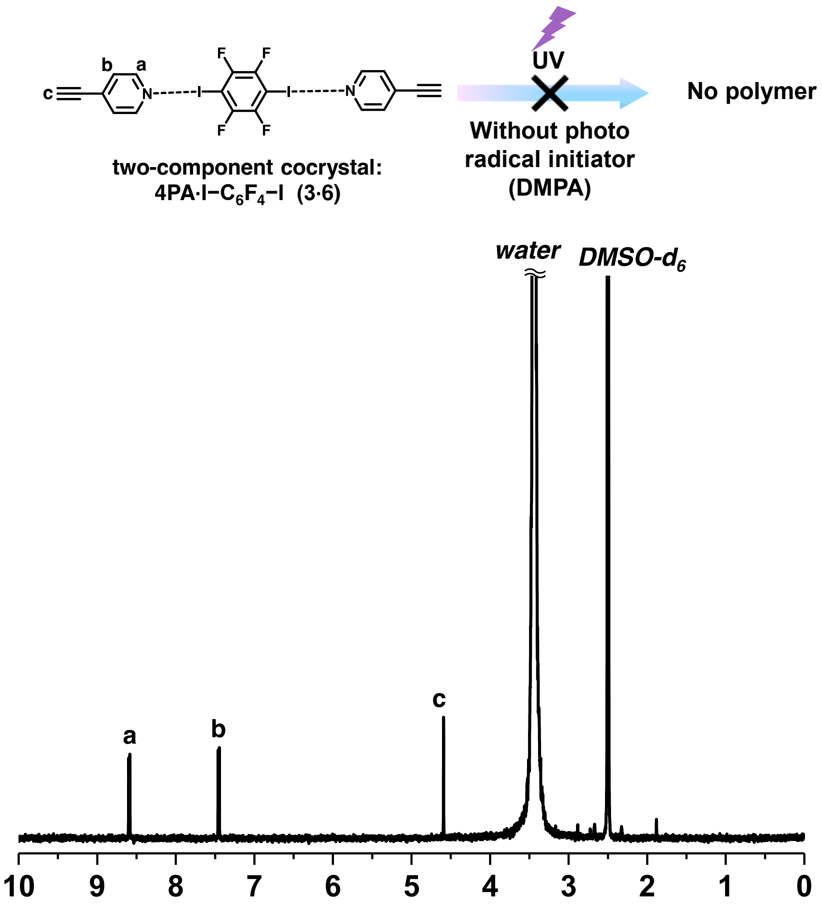

Supplementary Fig. 13: ^{1}H NMR spectrum of two-component monomer cocrystal **3·6** (without photo-initiator) after UV irradiation at $\lambda = 365$ nm for 40 h (400 MHz, 298 K, DMSO-d_6).

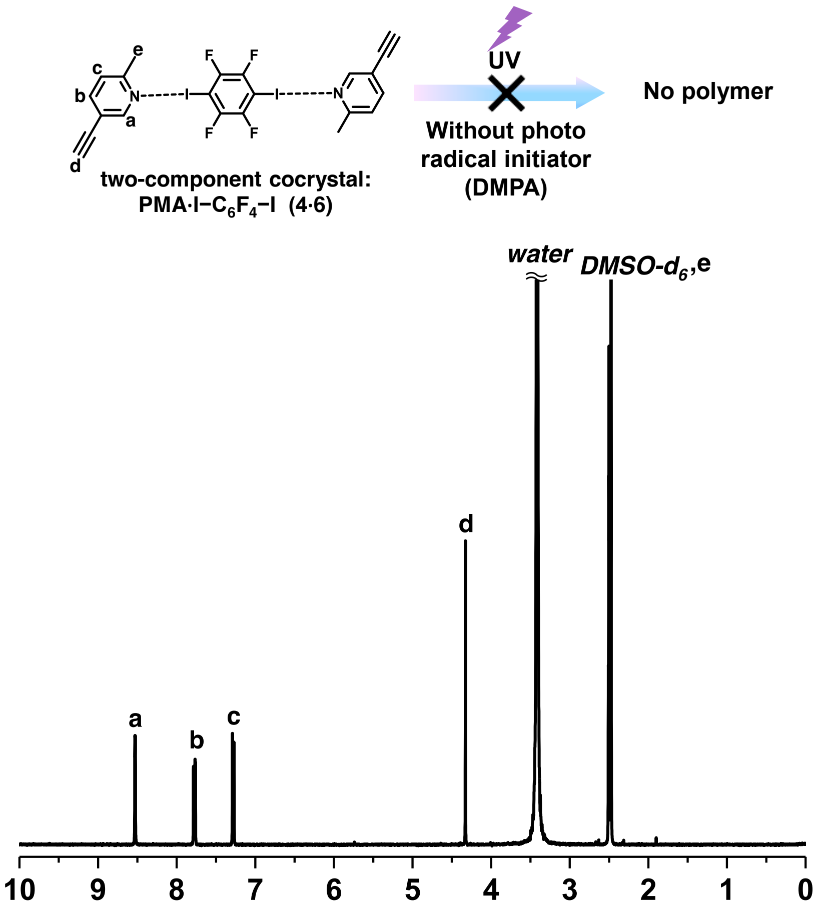

Supplementary Fig. 14: ^{1}H NMR spectrum of two-component monomer cocrystal **4·6** (without photo-initiator) after UV irradiation at $\lambda = 365$ nm for 40 h (400 MHz, 298 K, DMSO-d_6).

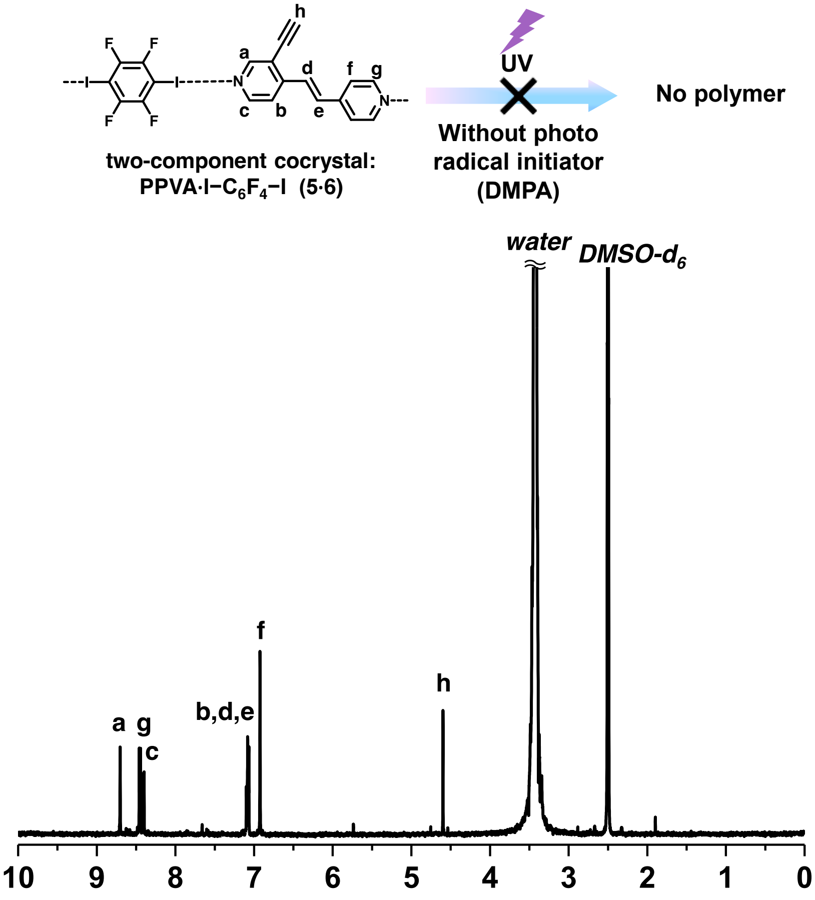

Supplementary Fig. 15: ^{1}H NMR spectrum of two-component monomer cocrystal **5·6** (without photo-initiator) after UV irradiation at $\lambda = 365$ nm for 40 h (400 MHz, 298 K, DMSO-d_6).

2.12. Powder X-Ray Diffraction.

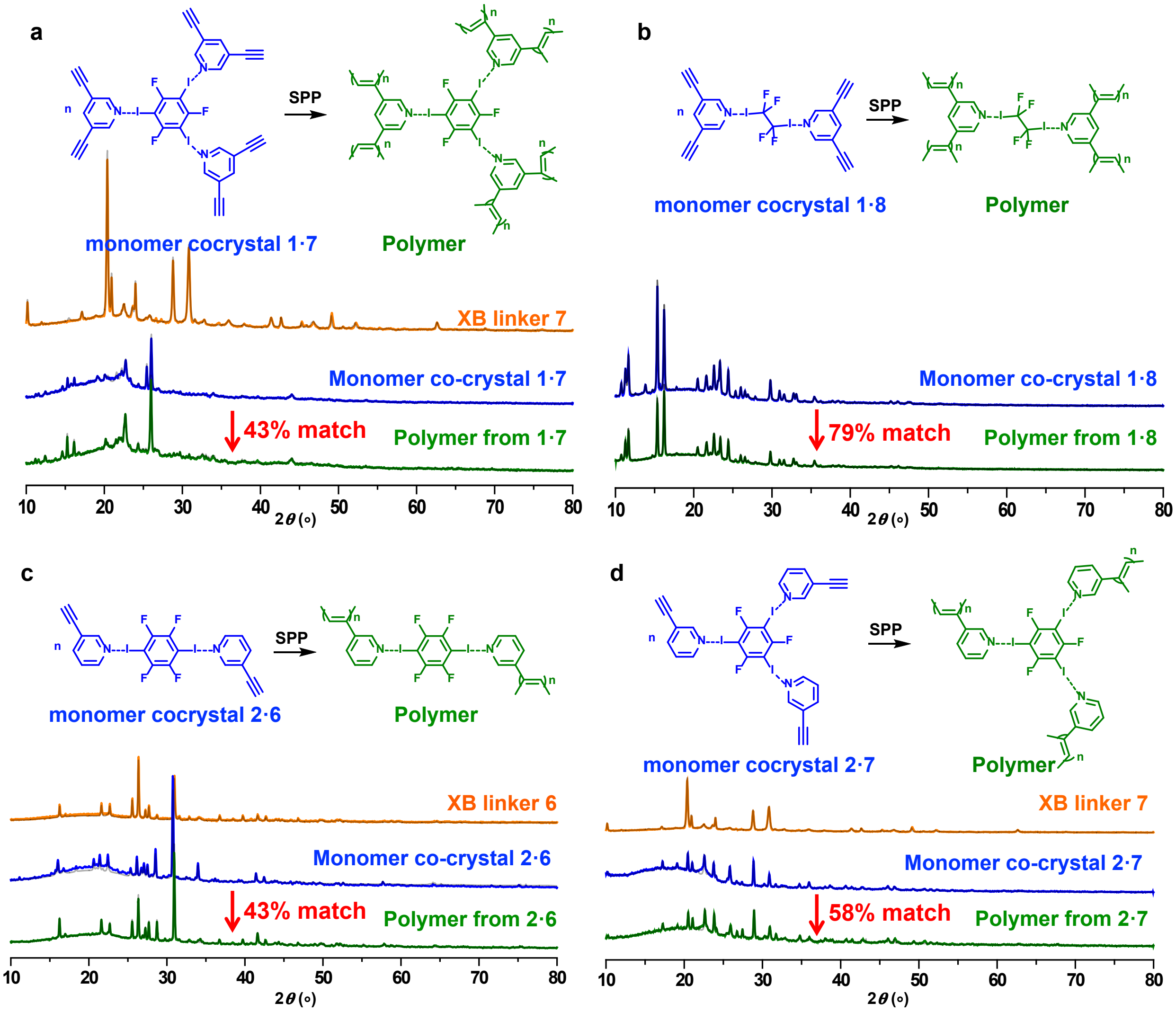

Supplementary Fig. 16: PXRD patterns of pure XB linkers (orange), monomer cocrystals (blue), and polymer solids obtained via SPP (green), and their calculated PXRD patterns (in grey color and overlapped with the experimental spectra). (**a**) PXRD patterns of pure XB linker **7** ($C_6F_3I_3$), monomer cocrystal **1·7**, and polymer solid from **1·7** via SPP. (**b**) PXRD patterns of monomer cocrystal **1·8** and polymer solid from **1·8** via SPP. (**c**) PXRD patterns of pure XB linker **6** (I–C_6F_4–I), monomer cocrystal **2·6,** and polymer solid from **2·6** via SPP. (**d**) PXRD patterns of pure XB linker **7** ($C_6F_3I_3$), monomer cocrystal **2·7,** and polymer solid from **2·7** via SPP. 43%, 79%, 43%, and 58% of PXRD patterns of polymers matched those of the monomer cocrystals in (**a**)–(**d**), respectively, showing the SPP is crystal-to-crystal polymerization.

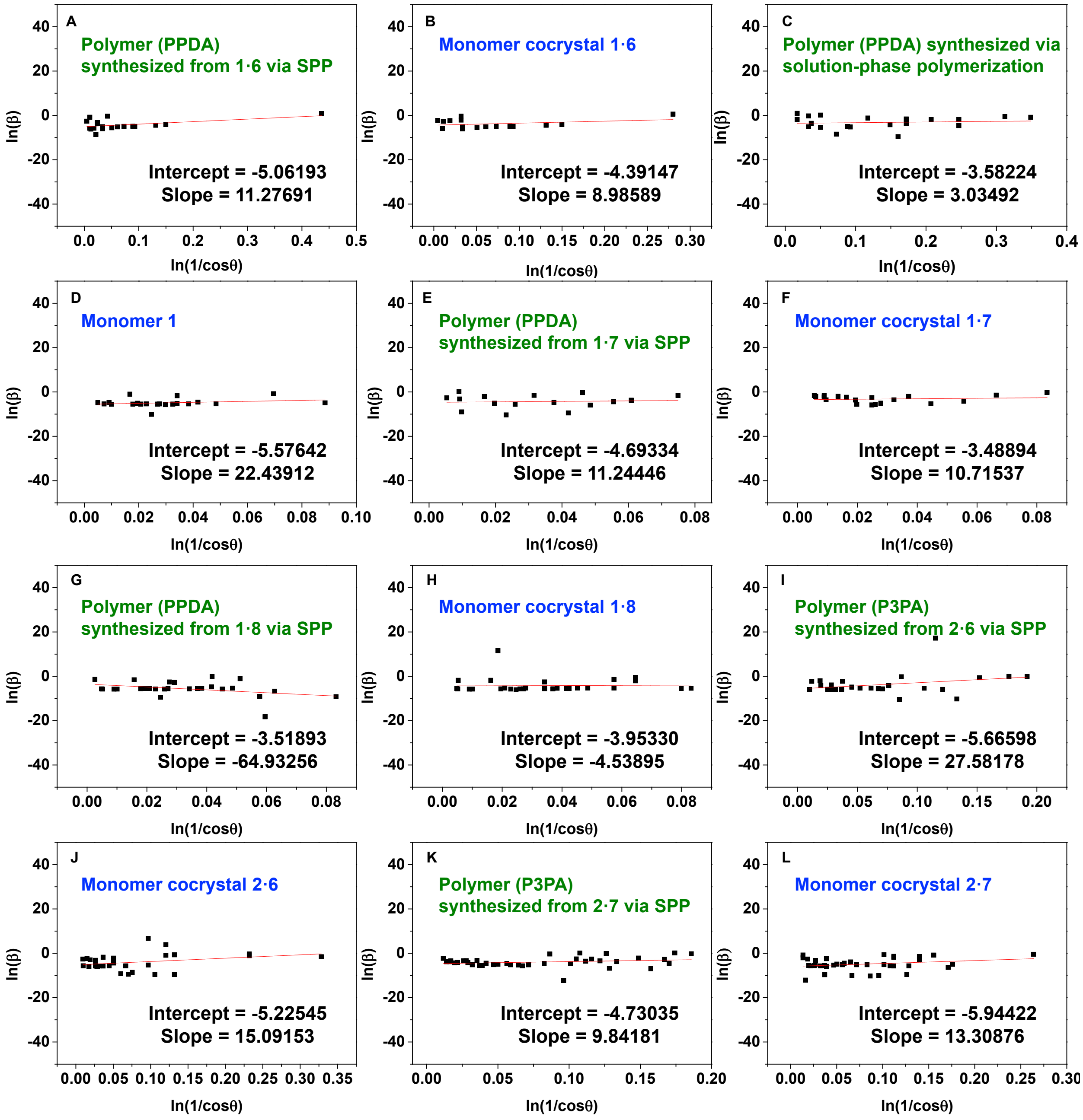

Supplementary Fig. 17: Modified Scherrer equation fittings (least square method) for plots of ln(β) vs ln(1/cosθ) from PXRD patterns (Figs. 1e and 1f, Supplementary Fig. 16, Supplementary equation (1), and Supplementary Table 3). (**A**) Polymer (PPDA) synthesized from **1·6** via SPP (Fig. 1e, green). (**B**) Monomer cocrystal **1·6** (Fig. 1e, blue). (**C**) Polymer (PPDA) synthesized via solution-phase polymerization (Fig. 1f, green). (**D**) Monomer **1** (Fig. 1f, blue). (**E**) Polymer (PPDA) synthesized from **1·7** via SPP (Supplementary Fig. 16a, green). (**F**) Monomer cocrystal **1·7** (Supplementary Fig. 16a, blue). (**G**) Polymer (PPDA) synthesized from **1·8** via SPP (Supplementary Fig. 16b, green). (**H**) Monomer cocrystal **1·8** (Supplementary Fig. 16b, blue). (**I**) Polymer (P3PA) synthesized from **2·6** via SPP (Supplementary Fig. 16c, green). (**J**) Monomer cocrystal **2·6** (Supplementary Fig. 16c, blue). (**K**) Polymer (P3PA) synthesized from **2·7** via SPP (Supplementary Fig. 16d, green). (**L**) Monomer cocrystal **2·7** (Supplementary Fig. 16d, blue).

2.13. FT-IR Spectra.

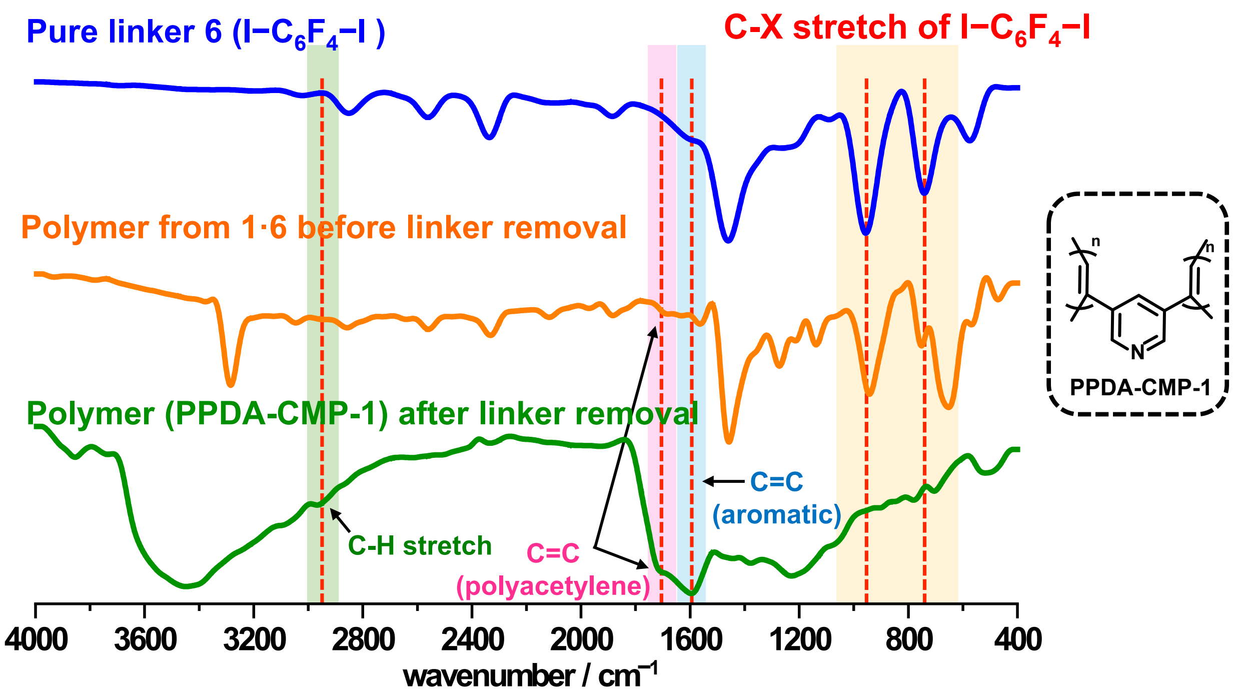

Supplementary Fig. 18: IR spectra of XB linker 6 (I−C_6F_4−I, blue), polymer from 1·6 before linker removal (orange), and polymer (PPDA-CMP-1) after linker removal (washing with ethanol) (green) (KBr). The =C–H stretch, non-aromatic C=C bonds (polyacetylene backbone), and aromatic C=C bonds (pyridine) appeared at 2968, 1698, and 1596 cm^{-1}, respectively. The C−F stretch at 956 cm^{-1} and C−I stretch at 741 cm^{-1} for XB linker **6** disappeared after washing with ethanol, indicating complete removal of XB linker **6**.

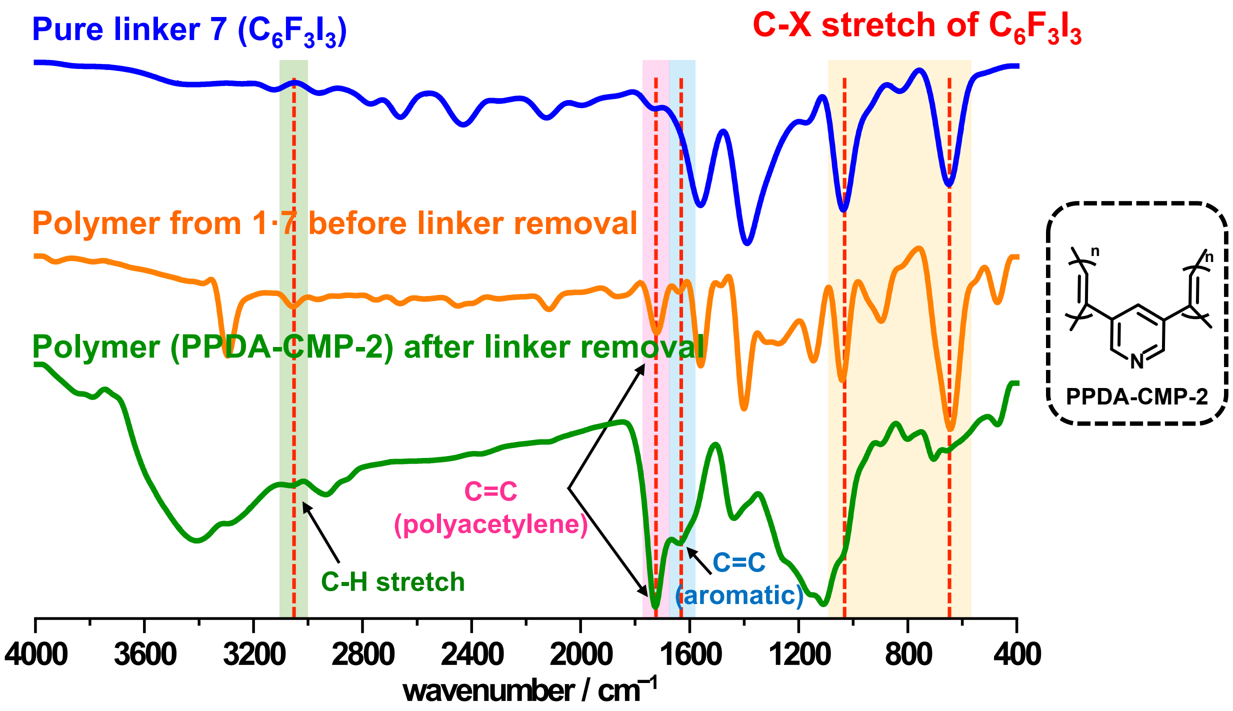

Supplementary Fig. 19: IR spectra of XB linker 7 ($C_6F_3I_3$, blue), polymer from 1·7 before linker removal (orange), and polymer (PPDA-CMP-2) after linker removal (washing with ethanol) (green) (KBr). The =C–H stretch, non-aromatic C=C bonds (polyacetylene backbone), and aromatic C=C bonds (pyridine) appeared at 3047, 1721, and 1639 cm^{-1}, respectively. The C−F stretch at 1036 cm^{-1} and C−I stretch at 649 cm^{-1} for XB linker **7** disappeared after washing with ethanol, indicating complete removal of XB linker **7**.

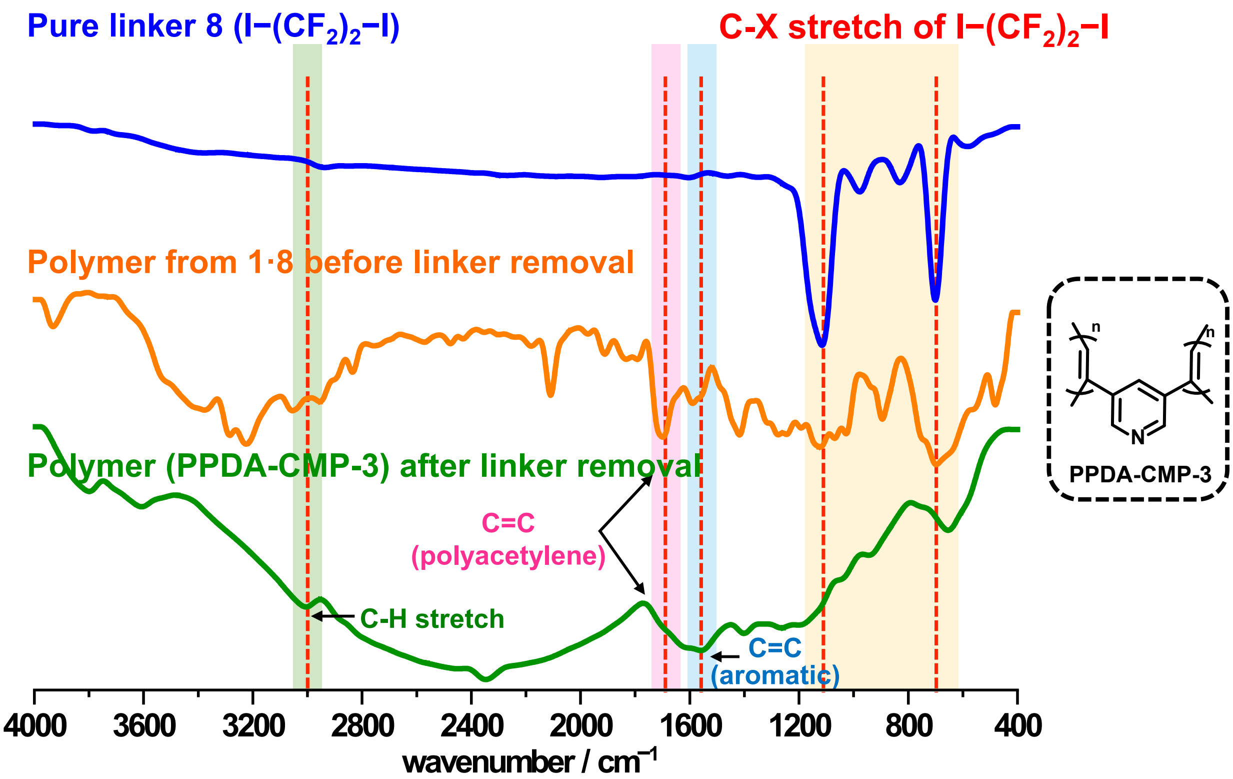

Supplementary Fig. 20: IR spectra of XB linker 8 (I−(CF_2)$_2$−I, blue), polymer from 1·8 before linker removal (orange), and polymer (PPDA-CMP-3) after linker removal (washing with ethanol) (green) (KBr). The =C–H stretch, non-aromatic C=C bonds (polyacetylene backbone), and aromatic C=C bonds (pyridine) appeared at 3005, 1698, and 1560 cm^{-1}, respectively. The C−F stretch of XB linker **8** at 1116 cm^{-1} and C−I stretch at 701 cm^{-1} disappeared after washing with ethanol, indicating complete removal of XB linker **8**.

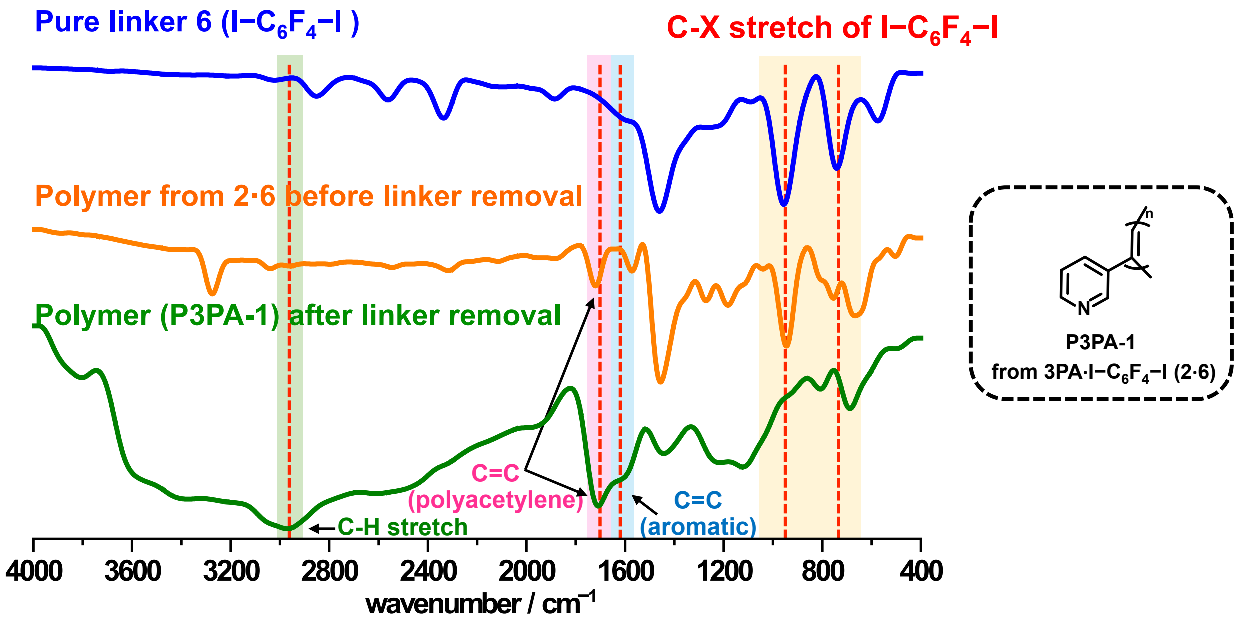

Supplementary Fig. 21: IR spectra of XB linker 6 (I−C_6F_4−I, blue), polymer from 2·6 before linker removal (orange), and polymer (P3PA-1) after linker removal (washing with ethanol) (green) (KBr). The =C–H stretch, non-aromatic C=C bonds (polyacetylene backbone), and aromatic C=C bonds (pyridine) appeared at 2970, 1709, and 1615 cm^{-1}, respectively. The C−F stretch of XB linker **6** at 956 cm^{-1} and C−I stretch at 741 cm^{-1} disappeared after washing with ethanol, indicating complete removal of XB linker **6**.

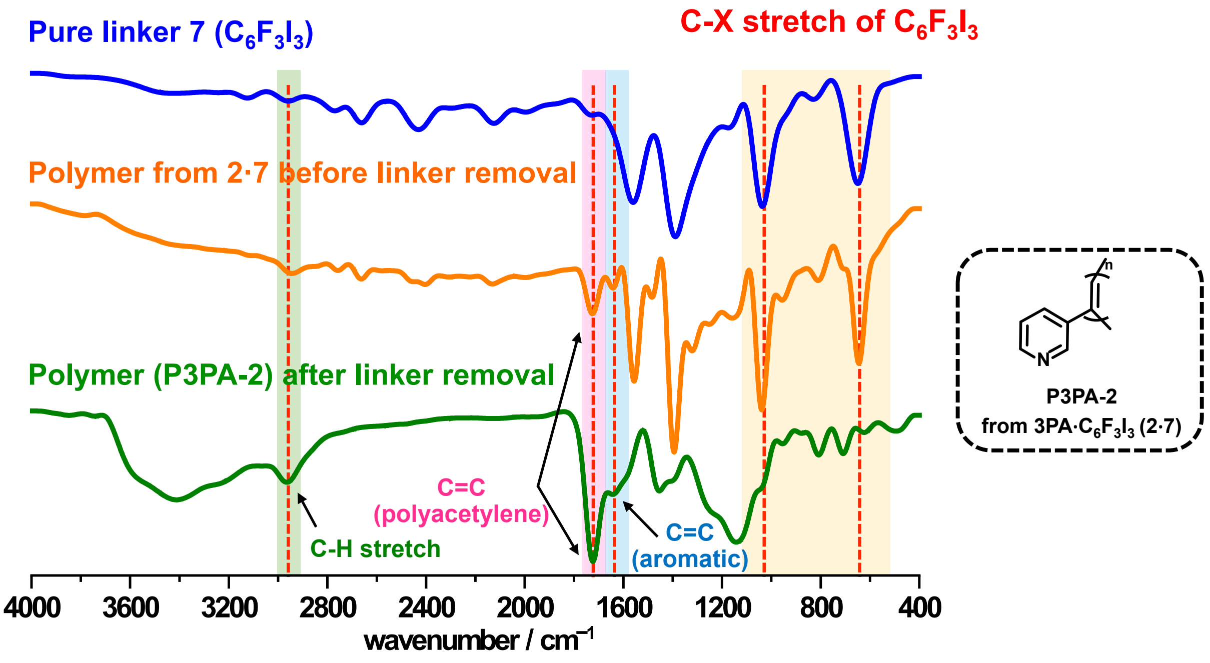

Supplementary Fig. 22: IR spectra of XB linker 7 ($C_6F_3I_3$, blue), polymer from 2·7 before linker removal (orange), and polymer (P3PA-2) after linker removal (washing with ethanol) (green) (KBr). The =C–H stretch, non-aromatic C=C bonds (polyacetylene backbone), and aromatic C=C bonds (pyridine) appeared at 2965, 1723, and 1644 cm^{-1}, respectively. The C−F stretch at 1036 cm^{-1} and C−I stretch at 649 cm^{-1} for XB linker **7** disappeared after washing with ethanol, indicating complete removal of XB linker **7**.

2.14. TEM & SEM Images.

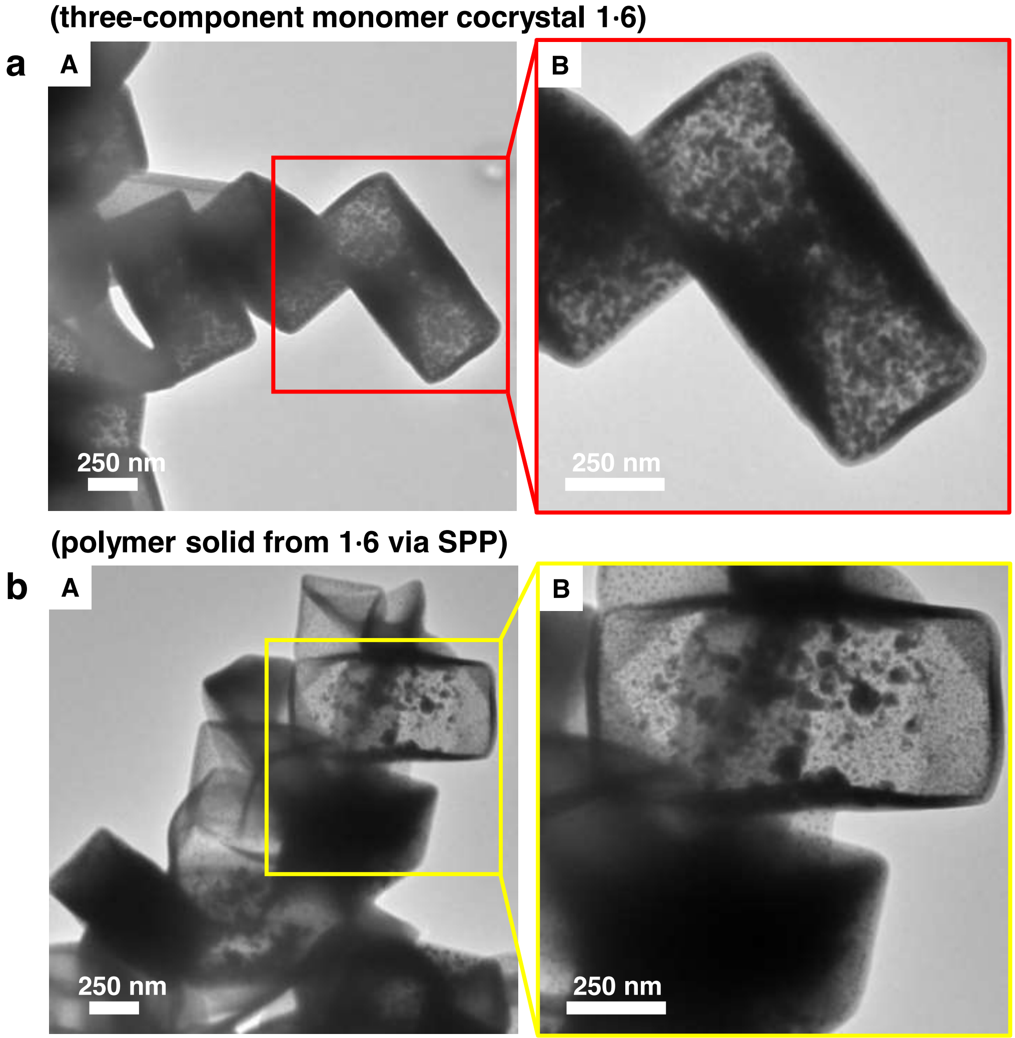

Supplementary Fig. 23: TEM images in the solid phase of (**a**) three-component monomer cocrystal **1·6** and (**b**) polymer solid from **1·6** via SPP.

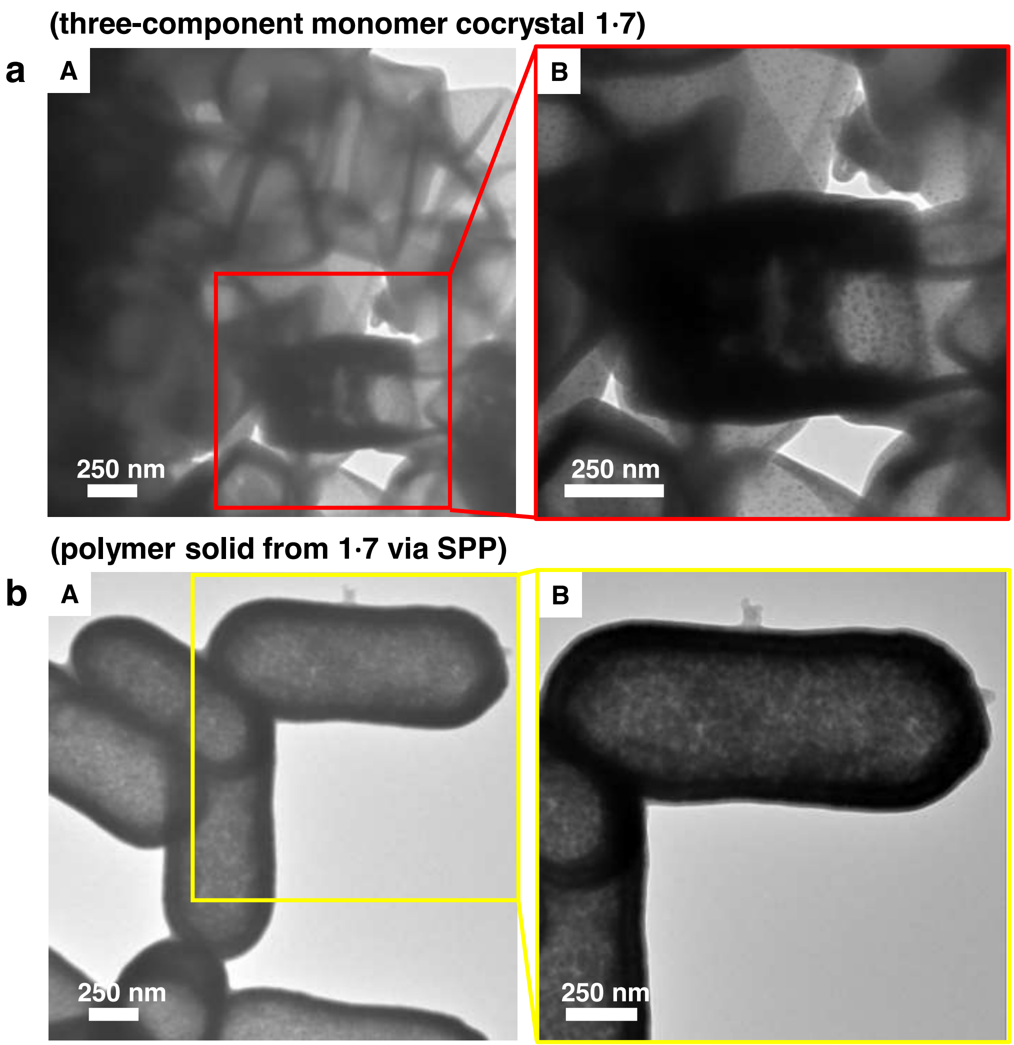

Supplementary Fig. 24: TEM images in the solid phase of (**a**) three-component monomer cocrystal **1·7** and (**b**) polymer solid from **1·7** via SPP.

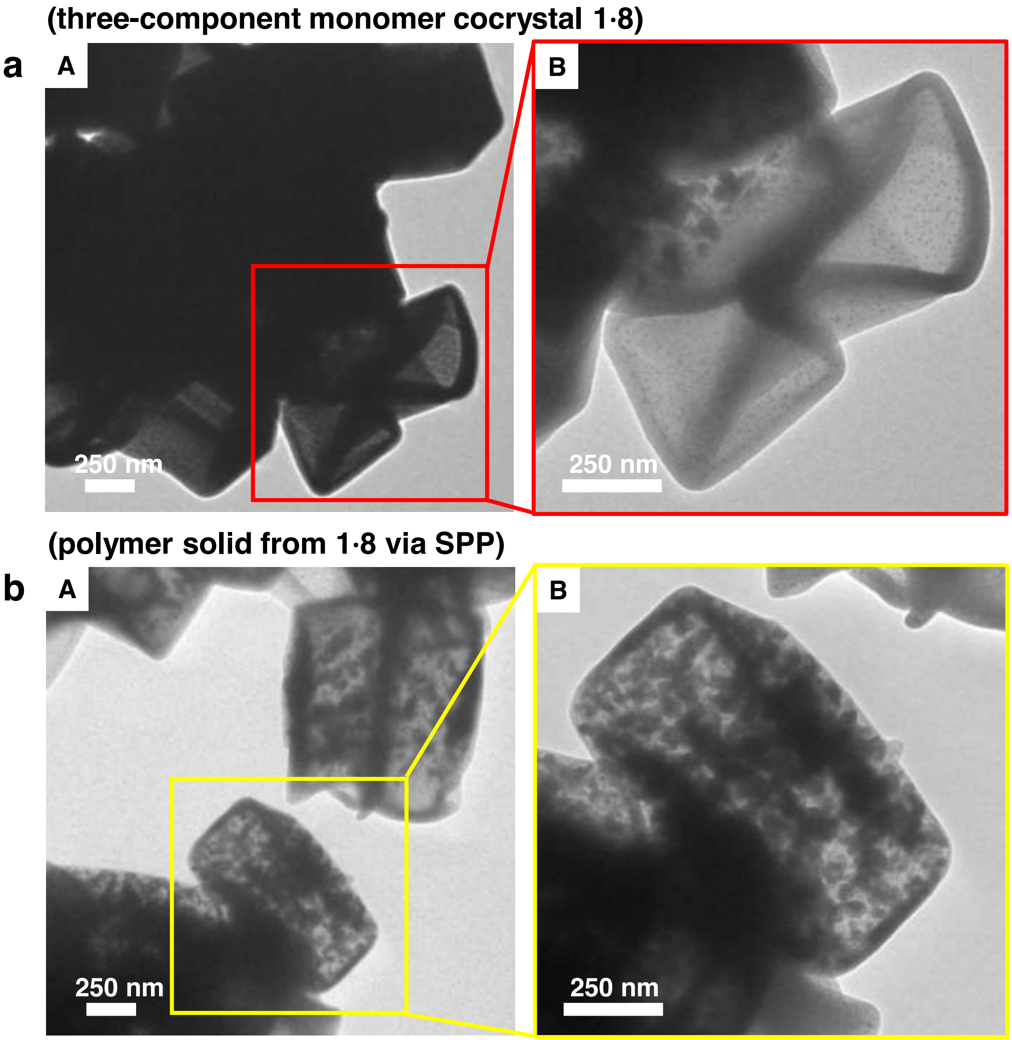

Supplementary Fig. 25: TEM images in the solid phase of (**a**) three-component monomer cocrystal **1·8** and (**b**) polymer solid from **1·8** via SPP.

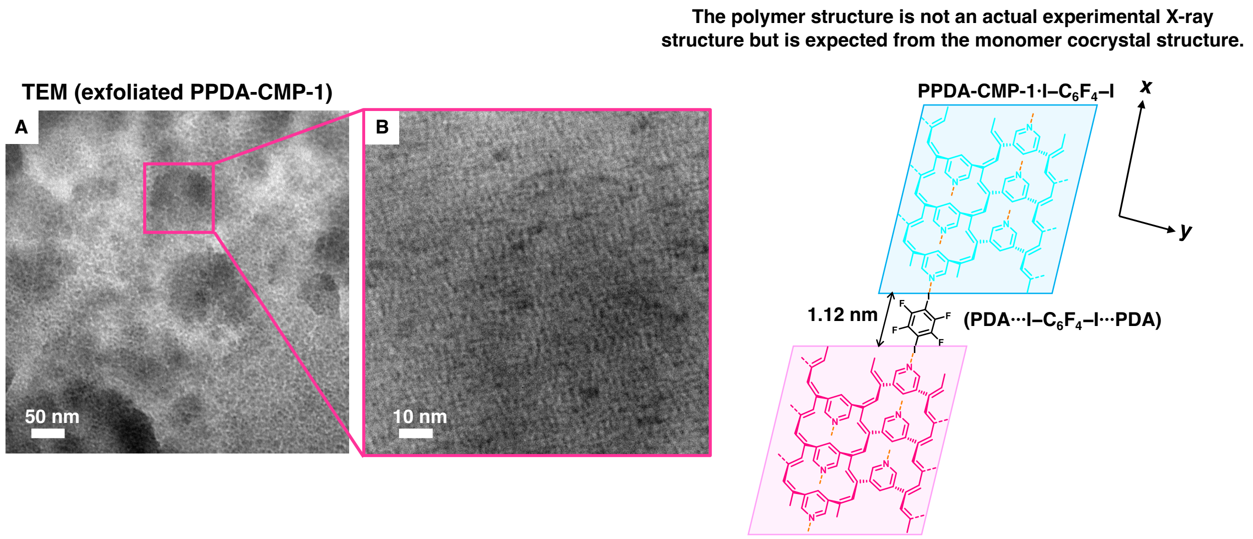

Supplementary Fig. 26: Additional TEM images of exfoliated PPDA-CMP-1 (exfoliated at 2×10^{-4} wt% of CMP in GBL) and a possible polymer structure expected from the monomer cocrystal (1·6) structure before linker removal. The TEM images show surface (image **A**) and single-chain nanopores (zoom-in image **B**) at the outermost layer of CMP. The polymer structure given in the figure is not an actual experimental X-ray structure but is expected from the monomer cocrystal structure.

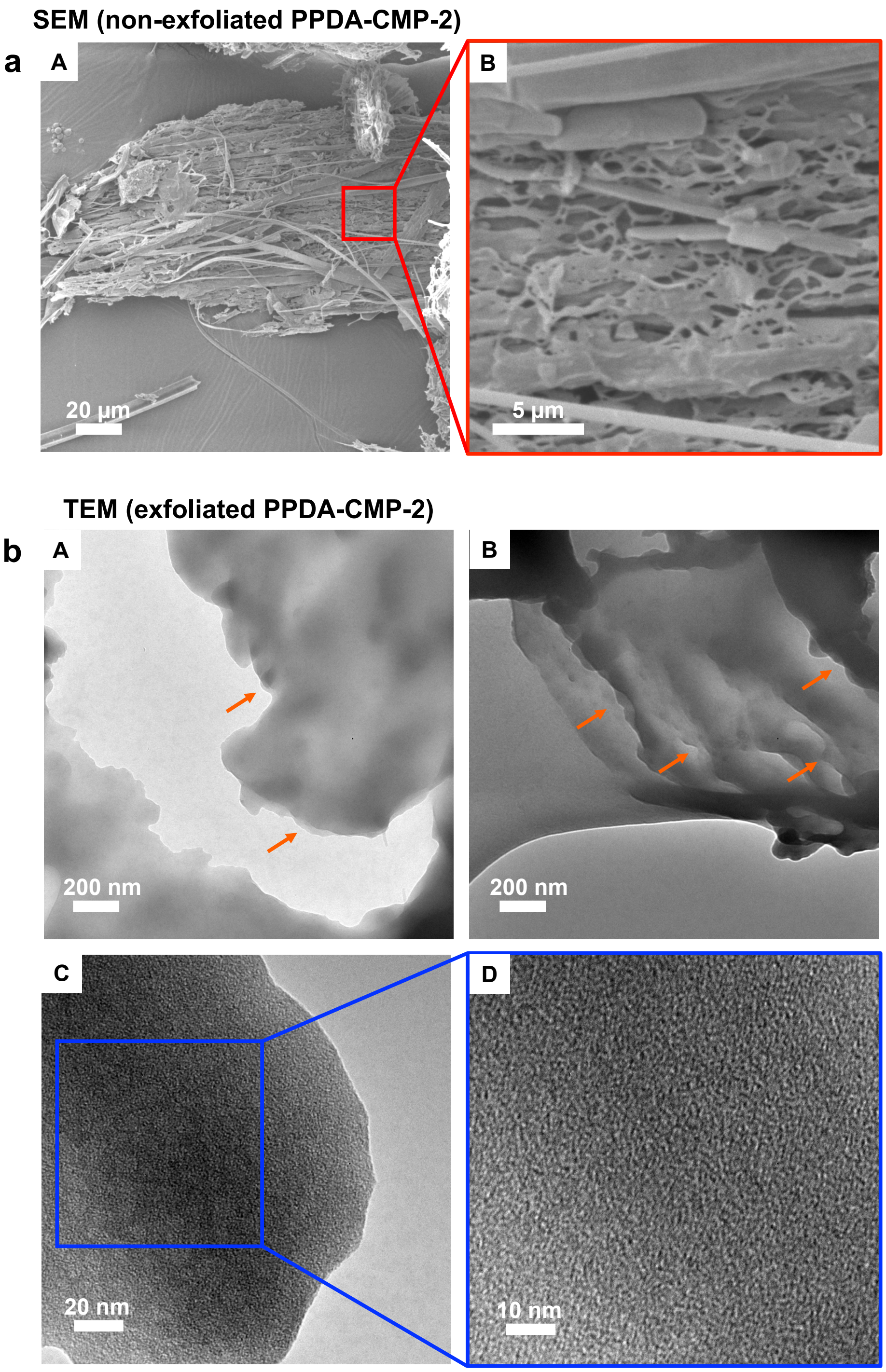

Supplementary Fig. 27: SEM images of non-exfoliated PPDA-CMP-2 and TEM images of exfoliated PPDA-CMP-2 (exfoliated at 2×10^{-4} wt% of CMP in GBL). (**a**) The SEM images show inter-grain micropores (after washing with ethanol). (**b**) The TEM images show surfaces (images **A** and **B**) and single-chain nanopores (zoom-in images **C** and **D**) at the outermost layer of CMP. The orange arrows in images **A** and **B** show edges where thin layered structures were particularly clearly observed.

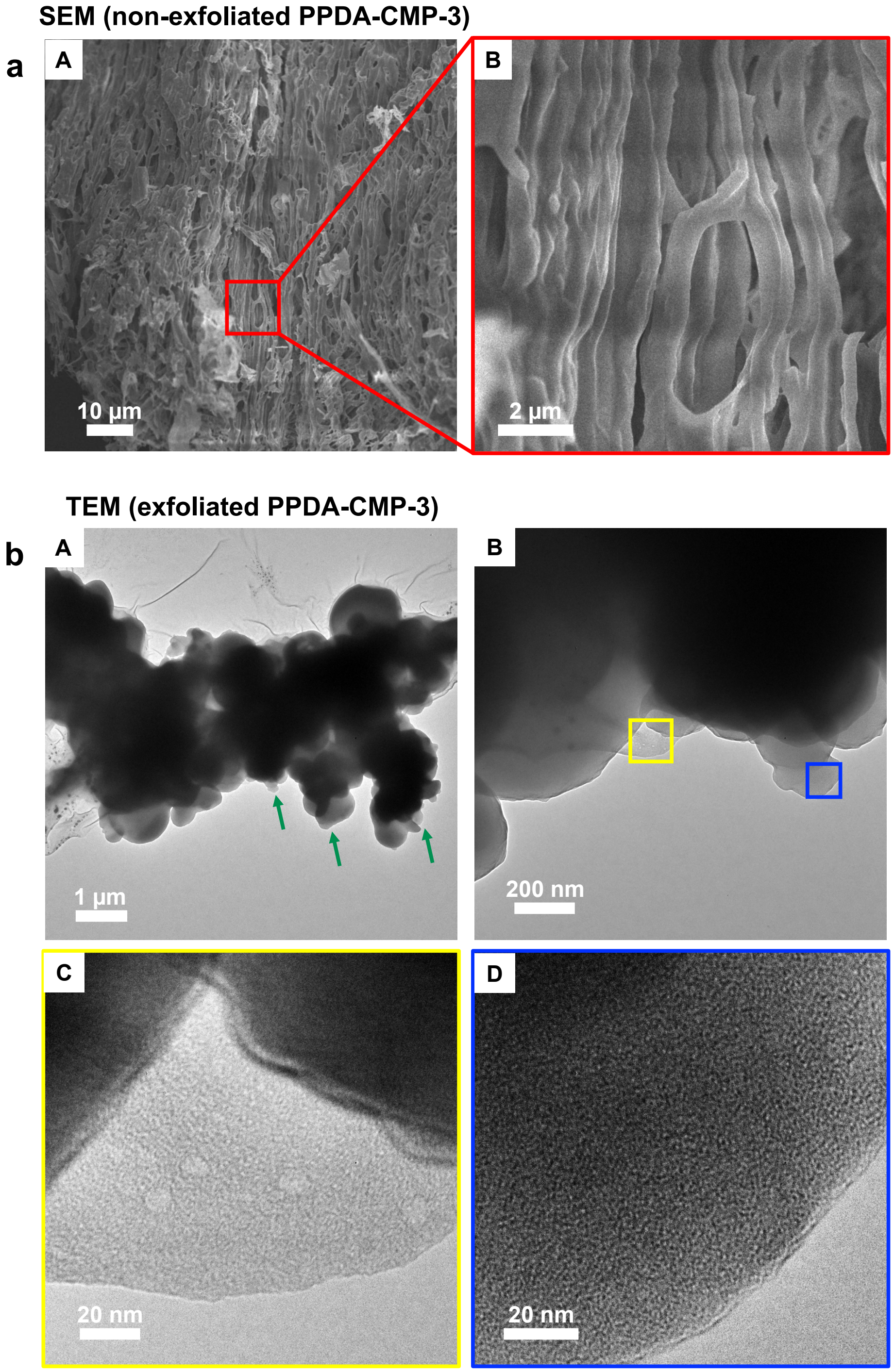

Supplementary Fig. 28: SEM images of non-exfoliated PPDA-CMP-3 and TEM images of exfoliated PPDA-CMP-3 (exfoliated at 2×10^{-4} wt% of CMP in GBL). (**a**) The SEM images show inter-grain micropores (after washing with ethanol). (**b**) The TEM images show surfaces (images **A** and **B**) and single-chain nanopores (zoom-in images **C** and **D**) at the outermost layer of CMP. The green arrows in image **A** show edges where thin layered structures were particularly clearly observed.

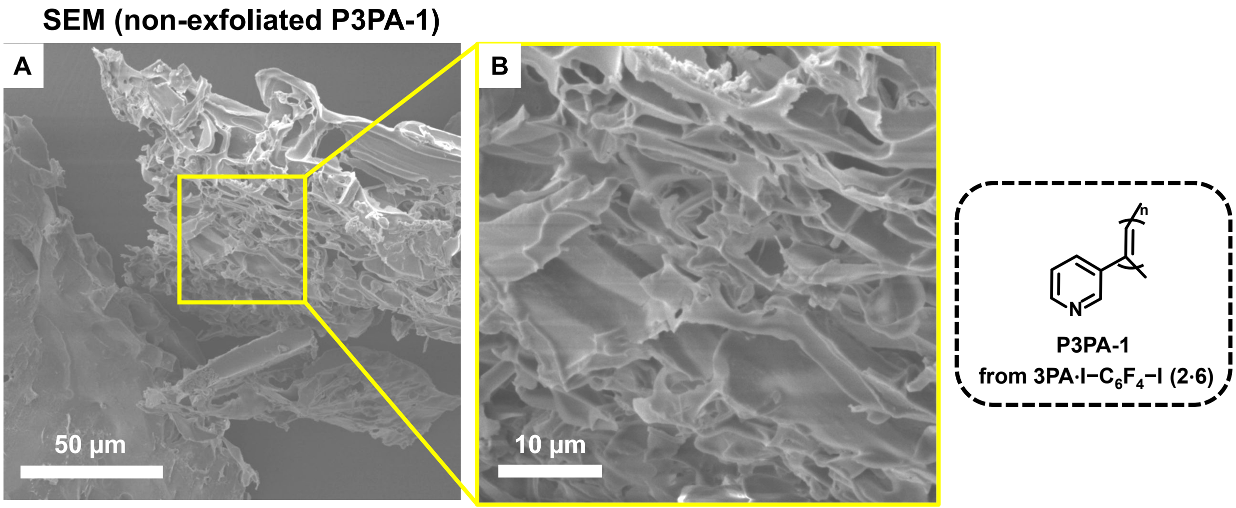

Supplementary Fig. 29: SEM images of non-exfoliated P3PA-1 polymer synthesized from 3PA·I–C_6F_4–I (2·6). The SEM images (image A and zoom-in image B) show inter-grain micropores (after washing with ethanol).

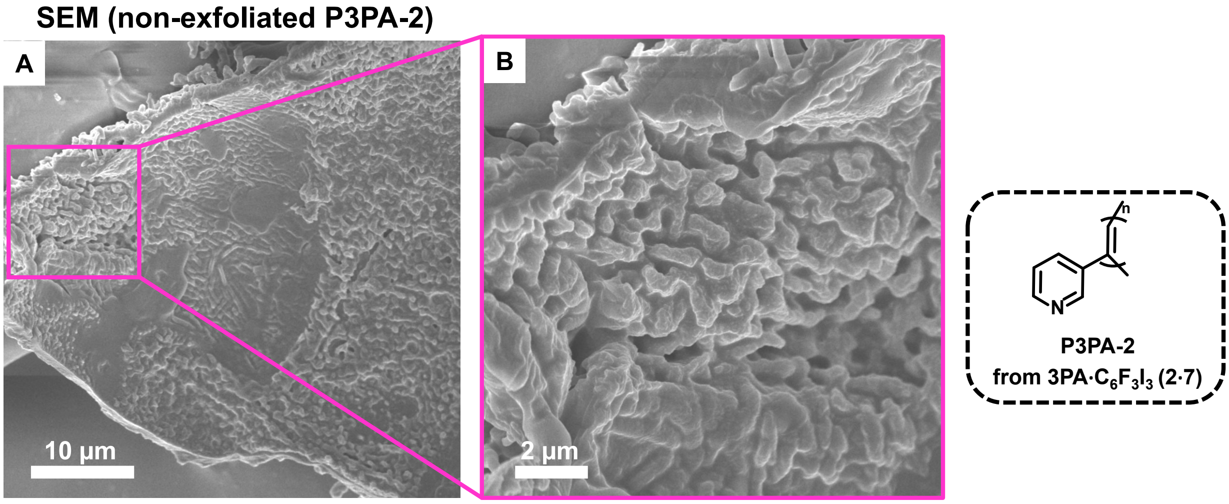

Supplementary Fig. 30: SEM images of non-exfoliated P3PA-2 polymer synthesized from 3PA·$C_6F_3I_3$ (2·7). The SEM images (image A and zoom-in image B) show inter-grain micropores (after washing with ethanol).

2.15. TGA Curves.

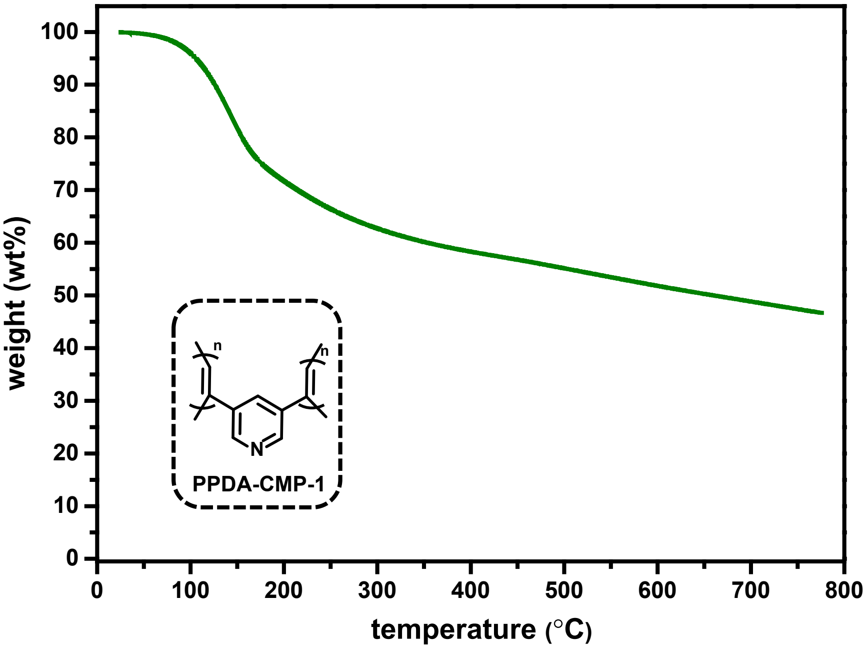

Supplementary Fig. 31: TGA curve of PPDA-CMP-1 (after washing with ethanol) at a heating rate of 10 °C/min under flowing air atmosphere. $T_{d\,(5\%)}$ = 106 °C, $T_{d\,(50\%)}$ = 667 °C.

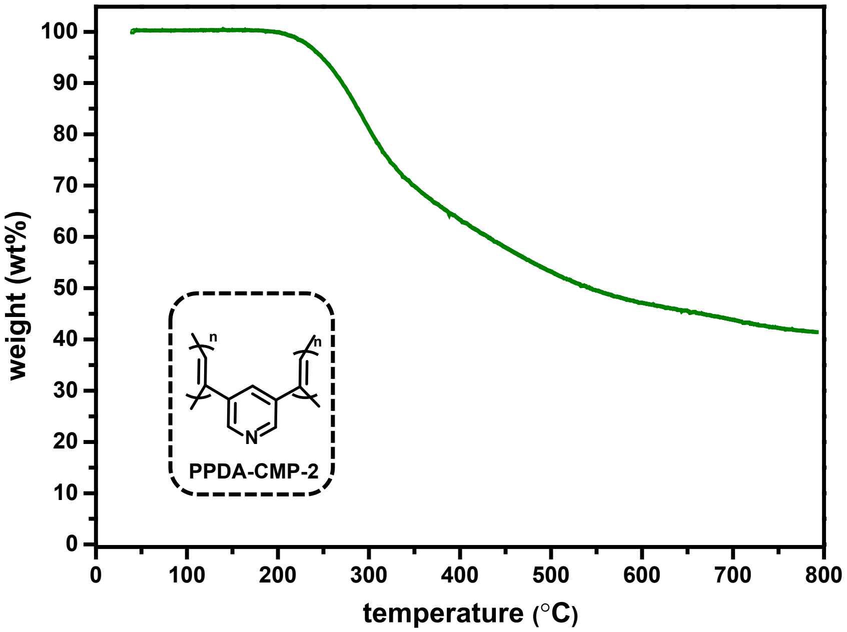

Supplementary Fig. 32: TGA curve of PPDA-CMP-2 (after washing with ethanol) at a heating rate of 20 °C/min under flowing air atmosphere. $T_{d\,(5\%)}$ = 249 °C; $T_{d\,(50\%)}$ = 543 °C.

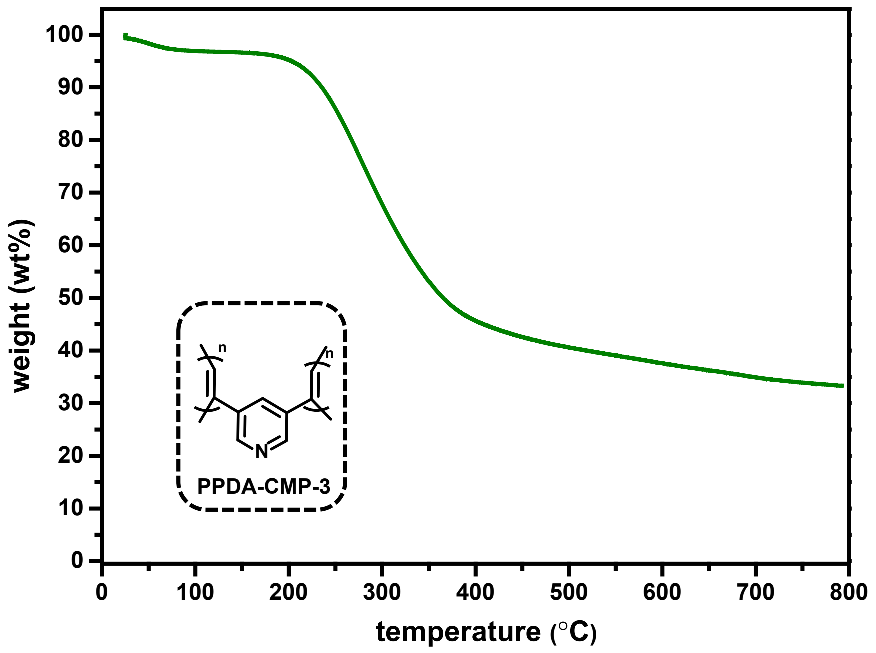

Supplementary Fig. 33: TGA curve of PPDA-CMP-3 (after washing with ethanol) at a heating rate of 20 °C/min under flowing air atmosphere. $T_{d\,(5\%)}$ = 190 °C; $T_{d\,(50\%)}$ = 363 °C.

2.16. AFM Images.

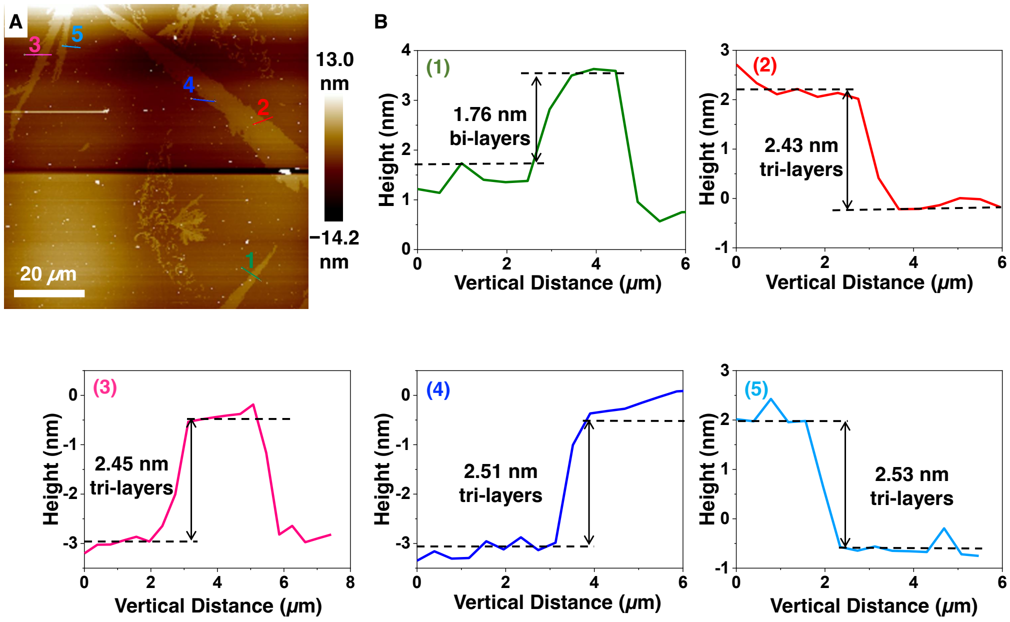

Supplementary Fig. 34: Additional AFM image and height profiles of exfoliated PPDA-CMP-1 (exfoliated at 0.1 wt% of CMP in GBL). (**A**) AFM image. (**B**) Height profiles.

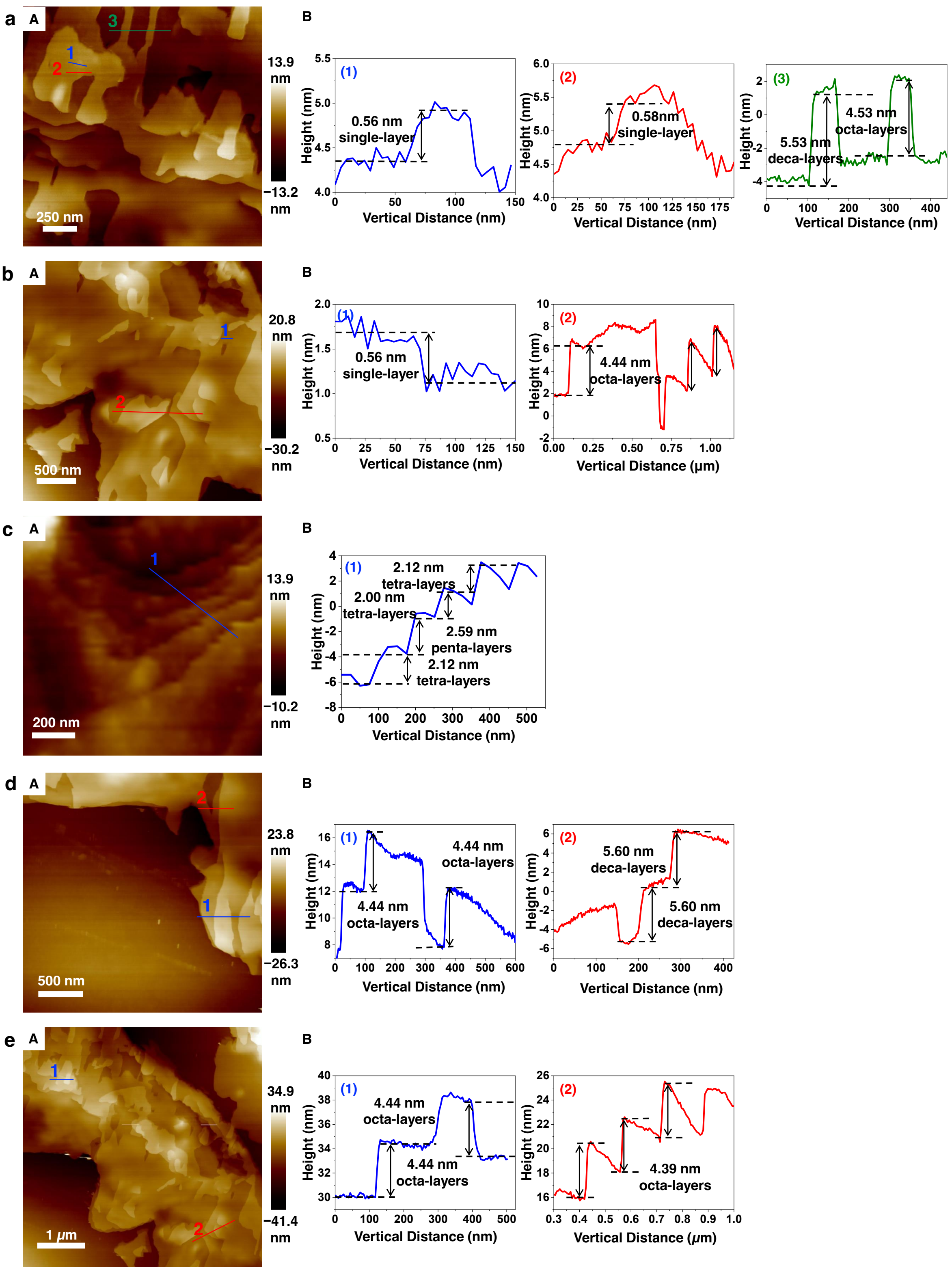

Supplementary Fig. 35: (a)–(e) AFM images and height profiles of exfoliated PPDA-CMP-2 (exfoliated at 2×10^{-4} wt% of CMP in GBL). (**A**) AFM image. (**B**) Height profiles.

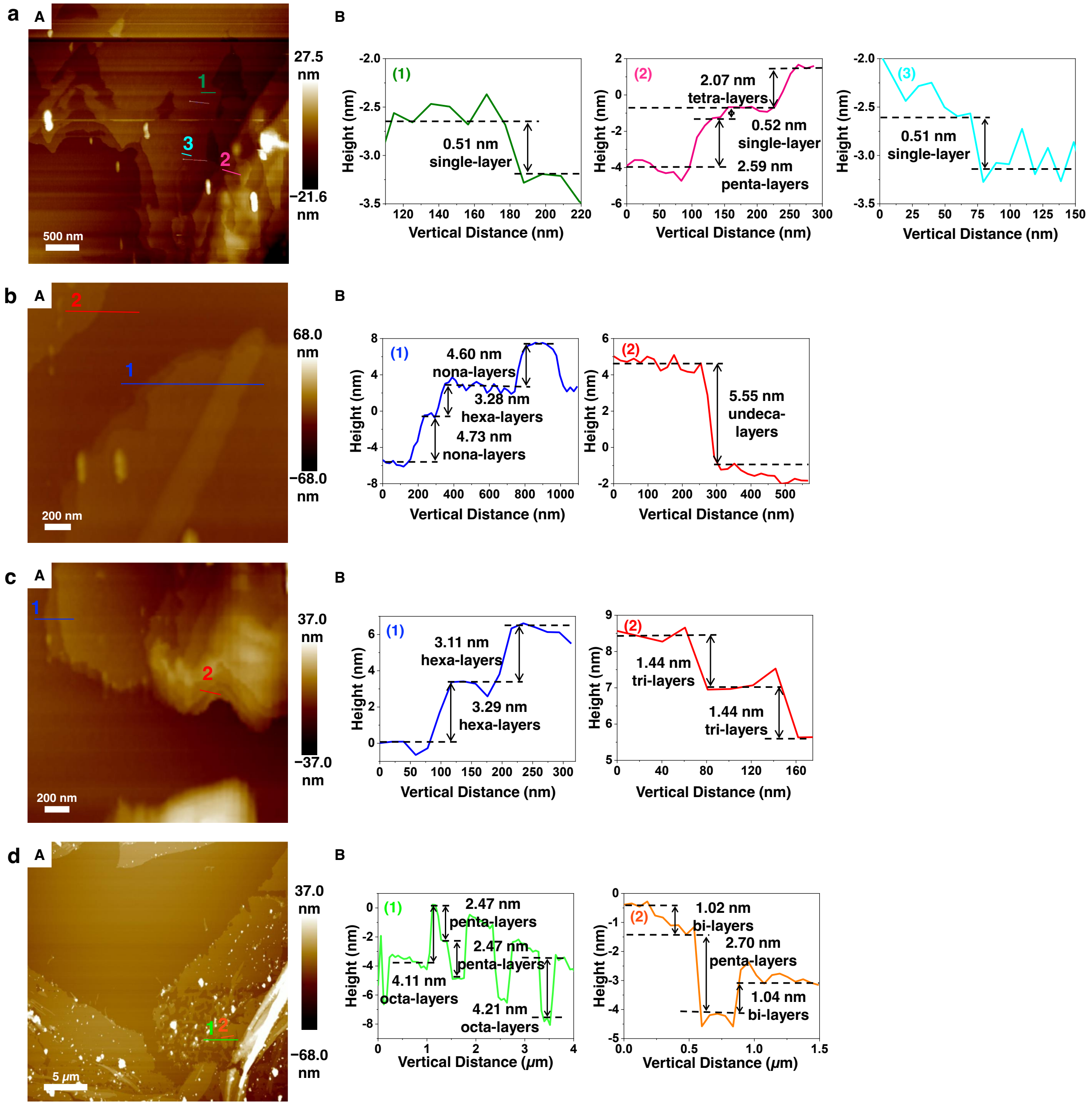

Supplementary Fig. 36: (a)–(d) AFM images and height profiles of exfoliated PPDA-CMP-3 (exfoliated at 2×10^{-4} wt% of CMP in GBL). (**A**) AFM image. (**B**) Height profiles.

2.17. BET Isotherms and Linear Plots.

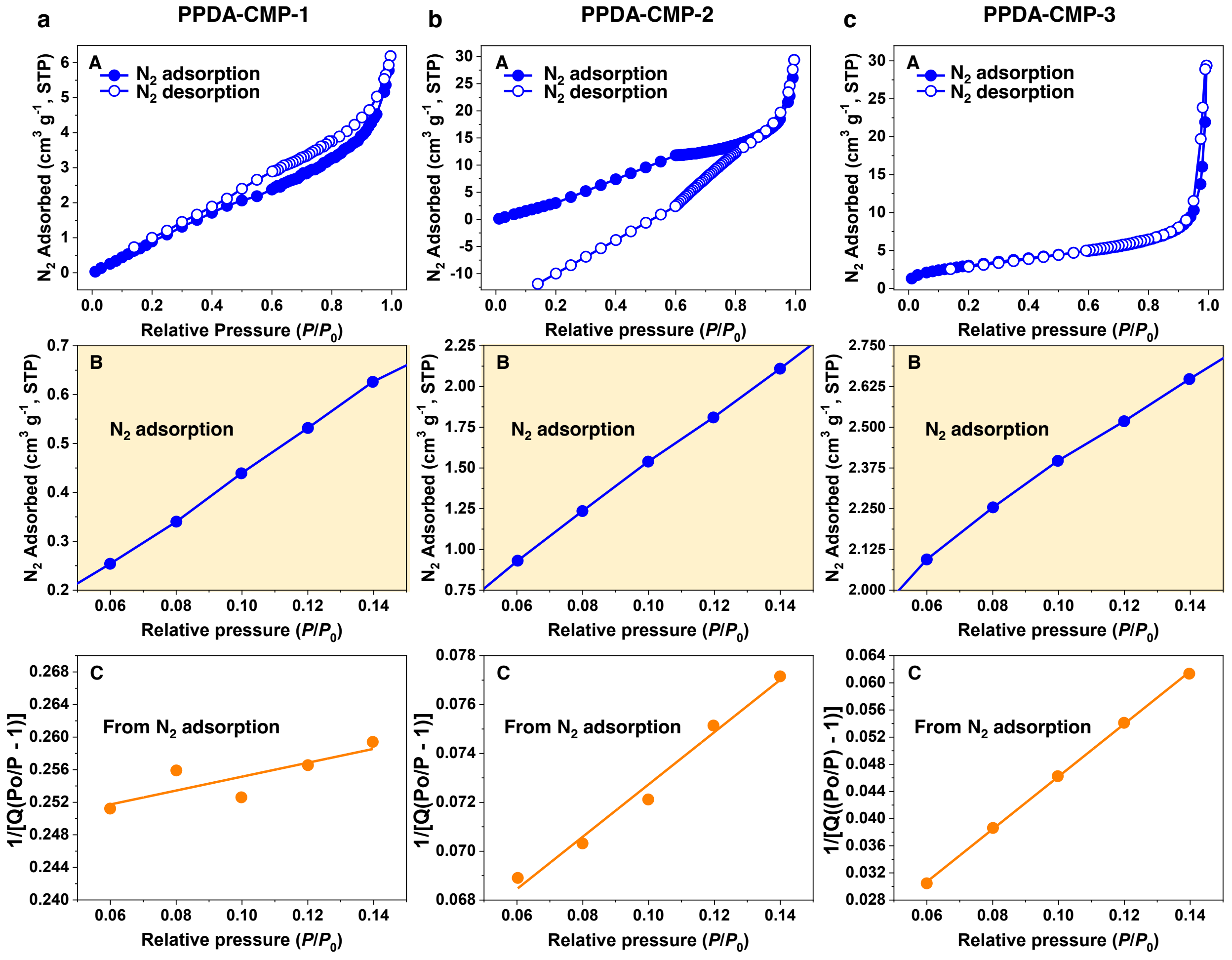

Supplementary Fig. 37: (A) BET nitrogen (N_2) adsorption-desorption isotherms, (B) N_2 adsorption isotherms at P/P_0 = 0.06−0.14, and (C) BET linear plots at P/P_0 = 0.06−0.14 (Supplementary equation (7)) of non-exfoliated PPDA-CMPs. (**a**) PPDA-CMP-1. (**b**) PPDA-CMP-2. (**c**) PPDA-CMP-3.

Supplementary Table 1: Single-crystal X-ray crystallography data of monomer cocrystals (Supplementary Figs. 2−7) and four possible monomer addition (propagation) patterns that can occur inside the cocrystals.

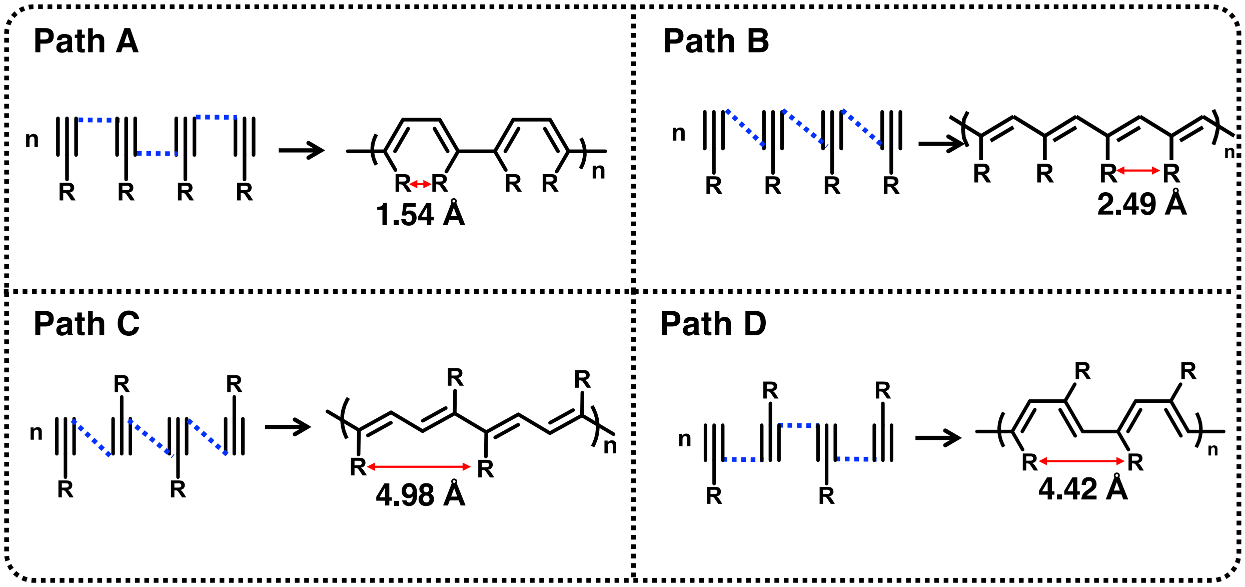

Entry	Co-crystal	$d_{\pi-\pi}$ (Å)[a]	$d_{C\equiv C\ adjacent}$ (Å)[b] Path A	Path B	Path C	Path D	R%[c]
1	**1·6**	3.885–4.321	3.535–4.813 (tt)<br>3.648–4.903 (hh)	3.393–4.232 (ht)	3.663–3.724 (tt)<br>4.066–4.097 (hh)	3.584–7.002 (ht)	4.58
2	**2·6**	4.187	4.349 (tt, hh)	4.068 (ht)	5.780 (tt)<br>4.616 (hh)	5.121 (ht shorter)<br>5.428 (ht longer)	4.72
3	**3·6**	5.086	6.107 (tt, hh)	5.379 (ht)	3.782 (tt)<br>5.059 (hh)	3.859 (ht)	5.49
4	**4·6**	3.733	5.133 (tt, hh)	4.775 (ht)	3.763 (tt)<br>4.792 (hh)	3.994 (ht shorter)<br>4.827 (ht longer)	3.64
5	**5·6**	7.391	7.782 (tt, hh)	6.337 (ht)	5.370 (tt)<br>5.588 (hh)	4.556 (ht shorter)<br>6.451 (ht longer)	4.13
6	**2·7**	3.571	3.719 (tt)<br>3.704 (hh)	3.479 (ht)	5.353 (tt)<br>4.958 (hh)	4.895 (ht shorter)<br>5.337 (ht longer)	3.37

[a]π–π distance between two linkers (average π–π distance between two linkers for entry 6). [b]hh = head-to-head, tt = tail-to-tail, and ht = head-to-tail. [c]R% is the R-factor in refinement using Bruker SHELXTL software, showing the discrepancy index between the experimental X-ray diffraction data and crystallographic model. Normally, R% less than 10% indicates a good fit.

Supplementary Table 2: Polymerizations of monomer cocrystal 1·6 under UV light (λ = 365 nm) at room temperature for varied polymerization time and varied amounts of photo-initiator (DMPA).

Entry	Varied Condition	$[1]_0/[6]_0/[DMPA]_0$	*t* (h)	Monomer conversion[a] (%)
			0.25	30
			3	33
1	Polymerization time	2/1/0.67	7	36
			24	59
			40[b]	100[b]
2	Amount of DMPA[c]	2/1/0.01	40	0
3		2/1/0.10	40	0

[a]Monomer conversions were determined with ^{1}H NMR for soluble polymers in ethanol. [b]Table 1, entry 1. [c]Amounts of DMPA at 0.67 equiv to linker and 0.335 equiv to monomer (entry 1), 0.01 to linker and 0.005 equiv to monomer (entry 2), and 0.10 equiv to linker and 0.05 equiv (entry 3) to monomer.

Supplementary Table 3: Powder X-ray diffraction (PXRD) data of crystallized monomers, non-crystallized monomers, and the respectively obtained polymers (Figs. 1e and 1f and Supplementary Figs. 16 and 17).

Entry	Compound	2θ (°)	FWHM (°)	d_{hkl} (nm)	Microstrain (ε)	Crystallite size (D) (nm)	Dislocation density (δ) (nm^{-2})	R_p (%)[a]	Match ratio (%)[b]
1	Polymer P(PDA·I–C_6F_4–I) (**1·6**)	10.91201	4.24175	0.810818	0.1937737	0.972958	1.056359	6.8	60%
		16.26794	25.64861	0.544877	0.78301467				
		16.26794	0.17165	0.544877	0.00524022				
		17.75867	0.11767	0.49946	0.00328646				
		21.5442	0.17578	0.41248	0.00403134				
		23.59873	0.01049	0.377014	0.00021911				
		24.94124	1.66006	0.357016	0.03275224				
		24.94124	2.24804	0.357016	0.04435282				
		29.57729	0.14194	0.302027	0.00234595				
		29.57748	0.35524	0.302025	0.00587129				
		36.11691	0.21648	0.2487	0.00289703				
		33.29202	39.52008	0.269128	0.57674268				
		39.6024	0.3224	0.227578	0.0039071				
		43.26075	0.41116	0.209143	0.00452418				
		47.64555	0.43468	0.190869	0.00429565				
		48.62404	0.40596	0.187254	0.00392088				
		57.45616	0.66506	0.160394	0.00529422				
		61.17923	0.92425	0.151495	0.00682192				
		99.45419	130.9202	0.101044	0.48398837				
2	Monomer cocrystal PDA·I–C_6F_4–I (**1·6**)	10.92663	5.6541	0.809736	0.25794566	0.968894	1.065239	4.6	NA
		16.26686	0.15388	0.544913	0.00469805				
		17.07599	4.02962	0.519271	0.11711616				
		22.24118	5.5981	0.399709	0.1242656				
		28.80383	39.43103	0.309959	0.66999881				
		28.80383	6.79728	0.309959	0.1154971				
		29.57728	0.13805	0.302027	0.00228166				
		29.57753	0.329	0.302025	0.00543759				
		36.11669	0.24223	0.248701	0.00324165				
		39.60242	0.33104	0.227578	0.00401181				
		43.26035	0.4051	0.209145	0.00445754				
		47.64556	0.42768	0.190869	0.00422648				
		48.62428	0.39774	0.187254	0.00384147				
		57.4583	0.65136	0.160388	0.00518493				
		61.18001	0.92742	0.151493	0.00684521				
		81.79344	104.587	0.117755	0.52688214				
3	Polymer PDA (**1**) synthesized in solution	21.08288	9.47516	0.4214	0.22217096	0.962003	1.080556	5.6	6%
		21.08288	145.9197	0.4214	3.42148599				
		29.2952	0.34129	0.304871	0.00569756				
		29.2952	46.06331	0.304871	0.76898947				
		30.9818	1.62464	0.288648	0.02557727				
		35.94859	0.24737	0.249825	0.00332699				
		35.94859	65.87125	0.249825	0.88593247				
		43.14715	0.01204	0.209667	0.00013287				
		47.51755	0.36	0.191353	0.00356842				
		48.51106	0.31266	0.187664	0.00302771				
		54.45379	17.63831	0.168505	0.14957842				
		60.99088	0.85523	0.151918	0.00633623				
		63.15792	0.00408	0.147217	2.8961E-05				
		65.28493	1.58414	0.142926	0.01079054				
		65.28493	11.60734	0.142926	0.07906468				
		71.28828	8.66091	0.132293	0.05269918				
		77.23758	8.94165	0.123521	0.04884077				
		77.23758	0.56649	0.123521	0.00309426				
		85.91169	33.61085	0.113134	0.15751142				
		90.24659	24.57447	0.108793	0.10676586				
4	Pure PDA (**1**) monomer solid	11.4013	0.44281	0.776129	0.01935516	0.975422	1.051029	5.8	NA
		13.78283	0.25034	0.642511	0.00903775				
		15.4069	0.48649	0.575129	0.01569281				

		16.17866	0.21829	0.547864	0.00670135				
		20.91469	21.25802	0.424751	0.5025522				
		21.57584	0.24047	0.411882	0.00550666				
		22.60064	0.3552	0.393432	0.00775603				
		23.26686	0.24586	0.382315	0.00521067				
		24.34932	0.26375	0.365559	0.0053342				
		25.34071	0.00235	0.351478	4.561E-05				
		26.51232	0.2438	0.336206	0.00451553				
		26.83257	0.25859	0.332266	0.00473019				
		27.8353	0.18558	0.32052	0.00326771				
		29.07741	0.24451	0.307105	0.00411384				
		29.76062	0.31994	0.300208	0.00525383				
		29.76062	10.20128	0.300208	0.1675181				
		31.52482	0.25956	0.283799	0.00401237				
		32.89816	0.58426	0.272259	0.00863452				
		35.38089	0.27899	0.253703	0.00381658				
		42.26099	26.39714	0.213856	0.29802163				
		47.51015	0.42404	0.191381	0.00420394				
5	Polymer P(PDA·$C_6F_3I_3$) (**1·7**)	11.83938	3.93701	0.747507	0.16567554	0.970866	1.060918	4.8	43%
		15.51053	2.41019	0.57131	0.07722022				
		15.35849	69.5766	0.576931	2.2515067				
		16.03413	0.00719	0.55277	0.00022274				
		20.85771	7.27088	0.425898	0.17236806				
		22.66974	0.36579	0.392249	0.00796228				
		25.97687	0.21695	0.343013	0.00410405				
		24.59166	0.00187	0.362011	3.7436E-05				
		28.65894	12.26978	0.311493	0.20958363				
		31.22384	0.5145	0.286466	0.00803398				
		32.94383	0.00444	0.271892	6.5521E-05				
		34.55045	43.46734	0.259608	0.60986342				
		35.3989	0.15366	0.253578	0.00210092				
		37.83568	0.68341	0.237788	0.00870069				
		39.56927	1.38809	0.227761	0.01683726				
		43.79889	11.95823	0.206697	0.12979954				
6	Monomer cocrystal PDA·$C_6F_3I_3$ (**1·7**)	12.11337	11.5033	0.730661	0.4730484	0.961007	1.082797	4.9	NA
		12.6268	7.99281	0.701064	0.31521981				
		15.27517	5.62404	0.580059	0.18299924				
		15.27529	9.93507	0.580055	0.3232722				
		15.81986	1.59673	0.560208	0.05014498				
		18.81943	7.96993	0.471541	0.20984022				
		20.64408	5.24597	0.430257	0.12567994				
		22.49244	1.48149	0.3953	0.03250904				
		22.73856	0.21648	0.391077	0.00469755				
		25.43525	4.60841	0.350193	0.08909835				
		25.43525	0.16822	0.350193	0.00325234				
		26.01669	2.02E-01	0.342497	0.00381916				
		26.9093	0.36541	0.331335	0.0066644				
		28.94232	1.62497	0.308507	0.02747308				
		31.05245	7.68087	0.288008	0.12063356				
		33.9569	0.28485	0.264009	0.00407079				
		37.8586	0.84137	0.237649	0.01070474				
		41.30232	13.79153	0.218596	0.15966503				
		46.15986	46.93403	0.19666	0.48058595				
7	Polymer P(PDA·I−$(CF_2)_2$−I) (**1·8**)	8.29641	14.43338	1.065763	0.86833658	0.961333	1.082063	4.9	79%
		11.26248	0.19632	0.785664	0.00868758				
		11.66005	0.18212	0.758963	0.0077826				
		15.351	0.17353	0.577211	0.00561822				
		16.21849	0.18278	0.546527	0.00559725				
		20.27508	11.88466	0.438003	0.29002061				
		21.63704	0.21287	0.410731	0.00486051				
		22.59076	0.2333	0.393602	0.00509655				
		23.38476	0.24892	0.380415	0.00524818				
		24.42399	0.18533	0.364459	0.00373639				
		25.29223	0.00448	0.352141	8.7122E-05				
		25.99845	0.18044	0.342733	0.00341046				
		26.54908	0.21671	0.335749	0.00400802				
		26.76203	4.49104	0.333125	0.08237587				
		27.53656	3.71451	0.323929	0.06614361				
		29.80077	0.20143	0.299813	0.00330308				

		30.95841	0.22947	0.288861	0.00361549				
		31.52517	0.25043	0.283796	0.00387119				
		32.82294	0.50063	0.272866	0.00741651				
		32.90607	51.60528	0.272195	0.76245738				
		34.1914	0.18389	0.262251	0.00260885				
		35.45269	0.27406	0.253205	0.00374104				
		36.3434	19.90475	0.247202	0.26459878				
		38.56833	0.00635	0.233438	7.9189E-05				
		39.14077	6.77E-07	0.230155	8.3052E-09				
		40.15697	0.06946	0.224562	0.00082916				
		45.13077	9.96E-45	0.200902	1.0462E-46				
		46.10813	0.00591	0.196869	6.0592E-05				
8	Monomer cocrystal PDA·I−(CF$_2$)$_2$−I **(1·8)**	11.29547	0.24688	0.783377	0.01089286	0.965507	1.072727	6.1	NA
		11.64764	0.1894	0.759769	0.00810238				
		11.80539	9.60867	0.749652	0.40552037				
		15.35119	0.17434	0.577204	0.00564438				
		16.21654	0.17914	0.546593	0.00548645				
		20.57346	9.41506	0.431718	0.22635185				
		22.03535	6.11E+06	0.403396	137022.357				
		22.59287	0.19834	0.393566	0.00433241				
		23.33004	0.28714	0.381294	0.00606861				
		24.41469	0.17979	0.364595	0.00362612				
		25.34714	0.13158	0.35139	0.00255309				
		26.01276	0.19249	0.342548	0.00363614				
		26.54553	0.19787	0.335793	0.00366009				
		26.91999	0.28864	0.331206	0.00526208				
		29.78138	4.52315	0.300004	0.07422174				
		29.79992	0.2117	0.299821	0.00347159				
		30.97161	0.20373	0.288741	0.0032085				
		31.54808	0.26045	0.283595	0.00402301				
		32.75548	0.24659	0.273412	0.00366102				
		33.11663	0.22919	0.270513	0.00336347				
		34.18257	0.29917	0.262317	0.00424549				
		35.4456	0.29952	0.253254	0.00408945				
		38.44271	13.75546	0.234172	0.17214596				
		38.44271	0.28249	0.234172	0.00353529				
		40.73983	33.64243	0.221483	0.3953492				
		40.73983	7.31807	0.221483	0.08599834				
		45.19062	0.23342	0.20065	0.00244732				
		46.09298	0.25839	0.19693	0.0026501				
9	Polymer P(3PA·I−C$_6$F$_4$−I) **(2·6)**	16.24212	0.14583	0.545738	0.00445915	0.975806	1.050203	6.0	43%
		17.62266	5.85482	0.503284	0.1648046				
		22.18362	7.5105	0.400733	0.16716032				
		22.52673	0.88077	0.394706	0.01929696				
		25.55401	0.1602	0.348592	0.0030824				
		26.34195	0.15069	0.338342	0.00280971				
		27.12922	1.15746	0.328699	0.0209323				
		27.68822	0.13355	0.322189	0.00236456				
		28.72878	0.14742	0.310752	0.00251174				
		30.94201	0.16617	0.28901	0.00261961				
		31.21858	5.98068	0.286513	0.0934056				
		34.23381	0.43935	0.261936	0.00622485				
		36.72281	0.2734	0.244734	0.00359417				
		38.41004	5.26E-43	0.234363	6.5851E-45				
		39.75865	0.27469	0.226719	0.00331473				
		41.63306	0.21352	0.216935	0.00245047				
		42.64958	0.19601	0.211997	0.00219081				
		44.18153	0.86685	0.204996	0.00931911				
		46.63457	0.0016	0.194768	1.6197E-05				
		47.0451	47.32559	0.193164	0.47439955				
		51.79203	0.26876	0.176521	0.00241548				
		53.96646	1.69E+09	0.169911	14451519.8				
		55.30156	0.15286	0.166121	0.00127306				
		57.81968	0.0021	0.159471	1.6592E-05				
		61.61491	29.89019	0.150528	0.21871781				
		66.13039	52.07	0.141303	0.3489846				
		68.74071	50.69263	0.13656	0.32339622				
10	Monomer cocrystal	15.75844	4.19526	0.562378	0.13227136	0.973793	1.054548	6.2	NA
		16.0274	0.2007	0.553	0.00622027				

	3PA ·I–C_6F_4–I **(2·6)**	19.74146	5.63193	0.44972	0.14122821				
		21.3924	0.14558	0.415372	0.00336299				
		22.32117	2.90515	0.398295	0.06425097				
		26.08509	2.39553	0.341615	0.04512174				
		26.08509	0.79331	0.341615	0.01494263				
		26.15124	0.18435	0.340765	0.00346329				
		27.06296	0.14368	0.329489	0.00260501				
		27.51189	0.14416	0.324214	0.00256942				
		28.54313	0.18501	0.31273	0.00317358				
		30.76394	0.181	0.290642	0.00287074				
		33.98756	0.19781	0.263778	0.0028242				
		30.76394	9.87816	0.290642	0.15667194				
		36.12583	0.70599	0.24864	0.00944539				
		36.12583	6.34113	0.24864	0.08483748				
		39.31867	0.00566	0.229154	6.9129E-05				
		41.42971	0.19635	0.217953	0.00226552				
		42.45689	0.00445	0.212915	4.9986E-05				
		43.95989	0.01022	0.205978	0.00011048				
		49.61915	46300.01	0.18373	437.023609				
		49.61915	0.28682	0.18373	0.00270728				
		51.75665	0.00405	0.176633	3.6428E-05				
		55.10968	2840.426	0.166654	23.7525323				
		55.10968	23.52804	0.166654	0.19674886				
		57.56299	0.00386	0.160121	3.066E-05				
		57.56299	28.71936	0.160121	0.22811589				
		75.07011	39.8215	0.126539	0.22615409				
		75.07022	19.10313	0.126539	0.1084902				
		87.85187	12.21789	0.11113	0.05534775				
11	Polymer P(3PA·$C_6F_3I_3$) **(2·7)**	17.47337	5.72311	0.50755	0.1624954	0.97109	1.060427	5.3	58%
		19.12206	1.22417	0.464146	0.0317117				
		20.55643	1.25114	0.432071	0.0301047				
		21.01807	1.89647	0.422685	0.04460817				
		22.56784	0.76071	0.393997	0.01663539				
		23.75491	0.94919	0.37457	0.01969181				
		25.99713	1.96952	0.34275	0.03722745				
		26.83797	2.08049	0.3322	0.0380489				
		27.4315	0.82882	0.325146	0.01481741				
		28.84938	0.31568	0.30948	0.00535509				
		30.9762	0.22758	0.288699	0.00358355				
		31.80055	0.23215	0.281401	0.00355591				
		30.26726	2.18682	0.295298	0.03528114				
		32.87221	0.64975	0.272468	0.00961038				
		34.88362	0.33949	0.257204	0.00471479				
		35.95527	0.40142	0.249781	0.00539781				
		38.08209	0.56747	0.236306	0.00717429				
		38.92293	0.32061	0.231393	0.0039589				
		40.68704	0.29211	0.221758	0.00343758				
		41.52787	0.19446	0.21746	0.00223791				
		42.83034	0.31222	0.211144	0.00347352				
		45.91341	0.5836	0.197658	0.00601165				
		46.96858	40.53601	0.193461	0.40708229				
		49.45811	2.62E-04	0.18429	2.4784E-06				
		50.64517	0.51455	0.180246	0.00474481				
		51.60142	4.44901	0.177128	0.04015535				
		52.19495	63.9053	0.175253	0.56924568				
		53.13471	1.60433	0.172373	0.01399901				
		54.09095	4.1557	0.169549	0.03551764				
		55.39342	1.88425	0.165867	0.01566201				
		56.33318	49.14059	0.163321	0.40044517				
		56.8113	0.06566	0.16206	0.00052974				
		57.88296	1.32445	0.159312	0.01045076				
		60.94954	6.60909	0.152011	0.04900586				
		62.61472	0.05439	0.148363	0.00039021				
		64.39532	3.64744	0.144684	0.02527482				
		64.98885	0.63344	0.143506	0.00433939				
		65.69779	65.21297	0.142128	0.44070142				
		67.7092	45.23796	0.138387	0.29424747				
12	Monomer cocrystal	18.71944	26.20227	0.474037	0.69363127	0.976925	1.047797	4.8	NA
		18.71944	7.32543	0.474037	0.19392012				

3PA·$C_6F_3I_3$ (**2·7**)	20.58541	3.17E-04	0.43147	7.6193E-06
	21.99898	4.15808	0.404054	0.09334232
	22.65052	0.23155	0.392577	0.00504462
	23.83496	0.19357	0.37333	0.0040019
	25.92278	0.21399	0.343717	0.0040568
	26.38692	0.37158	0.337775	0.0069161
	26.48849	2.13524	0.336503	0.03958461
	28.93053	0.1904	0.30863	0.00322043
	31.00882	0.00368	0.288403	5.7882E-05
	30.94368	0.2247	0.288995	0.00354211
	31.74496	0.1631	0.281881	0.00250286
	31.74496	4.92506	0.281881	0.07557768
	34.77576	0.27237	0.257977	0.00379512
	36.00339	0.43469	0.249458	0.00583683
	38.05901	0.3283	0.236444	0.00415327
	38.73933	0.65628	0.232447	0.00814529
	40.92387	1.14535	0.220529	0.01339363
	41.35799	0.00246	0.218314	2.8438E-05
	42.83389	0.32759	0.211128	0.00364418
	46.0517	0.32942	0.197097	0.00338198
	46.95452	0.00202	0.193516	2.0293E-05
	49.35626	0.00237	0.184647	2.2506E-05
	50.73718	0.33851	0.179941	0.00311502
	50.73718	31.31188	0.179941	0.28813672
	52.31198	0.19483	0.174888	0.001731
	53.25142	0.18447	0.172023	0.00160555
	53.25142	9.07831	0.172023	0.07901396
	53.25142	14.50078	0.172023	0.12620896
	56.36855	0.00389	0.163227	3.1676E-05
	56.84769	0.1944	0.161965	0.00156721
	59.20702	12.65377	0.156062	0.09717769
	59.20778	3.64318	0.15606	0.02797825
	62.17799	24.81167	0.1493	0.17954483
	62.26614	24.85636	0.149109	0.17955572
	65.1447	0.09419	0.1432	0.00064332
	65.98668	0.38099	0.141575	0.0025605
	79.63941	35.17373	0.120389	0.18407687

[a]R_p is the R profile factor in Rietveld refinement, showing the discrepancy index between the experimental and calculated spectra. Normally, R_p less than 10% indicates a good fit. [b]Match ratio (%) = (the number of peaks of the polymer identically matched with those of the monomer (red))/ Σ{(the number of peaks of the polymer identically matched with those of the monomer) + (the number of shifted peaks in the polymer (blue)) + (the number of new peaks appeared in the polymer (green, if applicable)) + (the number of peaks present in the monomer but disappeared in the polymer (green, if applicable))} × 100%. The matching ratio was calculated in pairs (entries 1 vs 2, 3 vs 4, 5 vs 6, 7 vs 8, 9 vs 10, and 11 vs 12).

Supplementary Table 4: Conductivities of PPDA-CMP-1 and PPDA synthesized in solution-phase polymerization (Table 1, entries 1 and C1).

Entry	Synthetic mode	Polymer	Conductivity (σ) (S cm^{-1})	
			Before I_2 doping	After I_2 doping
1	SPP	PPDA-CMP-1	2.4×10^{-9}	2.7×10^{-4}
2	Solution	PPDA	NA ($< 10^{-9}$)[a]	8.3×10^{-5}

[a]Below the detection limit of the utilized instrument.

Supplementary Table 5: BET analysis data of PPDA-CMPs (Supplementary Fig. 37).

entry	CMP	c_{BET}[a]	Q_m (cm^3 g^{-1} STP)[b]	S_{BET} (m^2 g^{-1})[c]	V_{BET} [$\times 10^{-3}$] (cm^3 g^{-1})[d]	d_{BET} (nm)[e]
1	PPDA-CMP-1	1.3	3.0	13	9.6	2.9
2	PPDA-CMP-2	2.7	5.9	26	46	7.1
3	PPDA-CMP-3	53	2.5	11	46	17

[a]BET constant. [b]Monolayer adsorbed gas volume. [c]BET specific surface area. [d]Volume of adsorbed gas at $P/P_0 = 0.99$. [e](Average) pore diameter.

Supplementary Table 6: Metal-ion adsorption of PPDA-CMPs and PPDA synthesized in solution-phase polymerization.

Entry	Synthetic mode	Polymer	Metal solution	Metal ion concentration in solution (mg L^{-1})			Amount of metal ion adsorbed per CMP (mg/g CMP)
				Before adsorption[a]	After adsorption[a]	Difference[b]	
1	SPP	PPDA-CMP-1	$LiOH$[c]	1026.5 (± 5.0)	948.5 (± 7.0)	78.0 (± 7.0)	312 (± 28)
2	SPP	PPDA-CMP-2		1042 (± 8)	1021 (± 10)	21 (± 10)	84 (± 40)
3	SPP	PPDA-CMP-3		1042 (± 8)	985.0 (± 13.5)	57.0 (± 13.0)	228 (± 52)
C1	Solution	PPDA		971.5 (± 2.0)	958.0 (± 0.0)	13.5 (± 1.6)	54 (± 6)
4	SPP	PPDA-CMP-1	$LiOH$ + $RbOH$ + $CsOH$[d]	1000.0 (± 3.0) (Li)<br>1411.5 (± 5.0) (Rb)	923.5 (± 6.0) (Li)<br>1417.0 (± 10.5) (Rb)	76.5 (± 5.5) (Li)<br>–5.5 (± 9.5) (Rb)	306 (± 22) (Li)<br>0 (0–16) (Rb)
5	SPP	PPDA-CMP-2		1021.0 (± 5.0) (Li)<br>1286.5 (± 5.5) (Rb)	1003.0 (± 3.5) (Li)<br>1285.5 (± 6.5) (Rb)	18.0 (± 5.0) (Li)<br>1.0 (± 7.0) (Rb)	72 (± 20) (Li)<br>4 (0–32) (Rb)
6	SPP	PPDA-CMP-3		1021.0 (± 5.0) (Li)<br>1264.0 (± 7.5) (Rb)	979.0 (± 6.0) (Li)<br>1243.0 (± 7.0) (Rb)	42.0 (± 6.4) (Li)<br>21.0 (± 8.4) (Rb)	168 (± 26) (Li)<br>84 (± 34) (Rb)
7	SPP	PPDA-CMP-1	NH_4BF_4[e]	1057 (± 1)	1008 (± 2)	49 (± 2)	196 (± 8)
8	SPP	PPDA-CMP-2		943.1 (± 2.5)	938.9 (± 3.1)	4.2 (± 3.2)	17 (± 13)
9	SPP	PPDA-CMP-3		943.1 (± 2.5)	950.4 (± 1.8)	–7.3 (± 2.5)	0 (± 10)
C3	Solution	PPDA		890.1 (± 4.6)	888.3 (± 0.4)	1.8 (± 3.8)	7 (± 15)

[a]Uncertainty (error range) with three-time repeated analysis. [b]Difference in the concentrations before and after adsorption (C_0–C in equation (10) in Supplementary Information). The uncertainty is the root-mean-square averaged uncertainty in the concentration difference before and after adsorption. [c]0.1 wt% of Li^+ in water. [d]0.1 wt% of Li^+ + 0.1 wt% of Rb^+ + 0.1 wt% of Cs^+ in water. [e]0.1 wt% of B^{3+} in water.

Supplementary Table 7: Metal ions adsorption of PPDA-CMP-1 in three cycles.

Entry	Cycle	Metal Solution	Metal ion concentration in solution (mg L^{-1})			Amount of metal ion adsorbed per CMP (mg/g CMP)
			Before adsorption[a]	After adsorption[a]	Difference[b]	
1	first	LiOH[c]	1026.5 (± 5.0)	948.5 (± 7.0)	78.0 (± 7.0)	312 (± 28)
	second		1026.5 (± 5.0)	946.5 (± 4.0)	80.0 (± 5.2)	320 (± 21)
	third		1026.5 (± 5.0)	949.5 (± 5.5)	77.0 (± 6.1)	308 (± 24)
2	first	LiOH + RbOH + CsOH[d]	1000.0 (± 3.0) (Li)<br>1411.5 (± 5.0) (Rb)	923.5 (± 6.0) (Li)<br>1417.0 (± 10.5) (Rb)	76.5 (± 5.5) (Li)<br>–5.5 (± 9.5) (Rb)	306 (± 22) (Li)<br>0 (0–16) (Rb)
	second		1000.0 (± 3.0) (Li)<br>1411.5 (± 5.0) (Rb)	940.0 (± 9.5) (Li)<br>1423.5 (± 4.5) (Rb)	60.0(± 8.1) (Li)<br>–12.0 (± 5.5) (Rb)	240 (± 32) (Li)<br>0 (0–26) (Rb)
	third		1000.0 (± 3.0) (Li)<br>1411.5 (± 5.0) (Rb)	953.0 (± 1.5) (Li)<br>1472.0 (± 7.0) (Rb)	47.0 (± 2.7) (Li)<br>–60.5 (± 7.0) (Rb)	188 (± 11) (Li)<br>0 (~0) (Rb)
3	first	NH_4BF_4[e]	1057 (± 1)	1008 (± 2)	49 (± 2)	196 (± 8)
	second		1079 (± 2)	1036 (± 3)	43 (± 3)	172 (± 12)
	third		1079 (± 2)	1055 (± 1)	24 (± 2)	96 (± 8)

[a]Uncertainty (error range) with three-time repeated analysis. [b]Difference in the concentrations before and after adsorption (C_0–C in equation (10) in Supplementary Information). The uncertainty is the root-mean-square averaged uncertainty in the concentration difference before and after adsorption. [c]0.1 wt% of Li^+ in water. [d]0.1 wt% of Li^+ + 0.1 wt% of Rb^+ + 0.1 wt% of Cs^+ in water. [e]0.1 wt% of B^{3+} in water.

Supplementary Table 8: Single-crystal X-ray crystallography data and structure refinement for monomer cocrystals.[a]

Cocrystal monomer	PDA·I–C_6F_4–I (**1·6**)	3PA·I–C_6F_4–I (**2·6**)	4PA·I–C_6F_4–I (**3·6**)	PMA·I–C_6F_4–I (**4·6**)	PPVA·I–C_6F_4–I (**5·6**)	3PA·$C_6F_3I_3$ (**2·7**)
Empirical formula	$C_{39}H_{15}F_8I_4N_3$	$C_{20}H_{10}F_4I_2N_2$	$C_{13}H_5F_4I_2N$	$C_{11}H_7F_2IN$	$C_{23}H_{10}F_6I_3N_2$	$C_{20}H_{10}F_3I_3N_2$
Crystal habit	Colorless block	Colorless plate	Colorless needle	Colorless needle	Colorless block	Colorless plate
Formula weight	1185.14 g mol^{-1}	608.10 g mol^{-1}	504.98 g mol^{-1}	318.08 g mol^{-1}	809.03 g mol^{-1}	716.00 g mol^{-1}
Crystal system	Triclinic	Triclinic	Monoclinic	Monoclinic	Triclinic	Monoclinic
Space group	P –1	P –1	P 1 21 1	P 1 21/n 1	P –1	C 1 2/c 1
Unit cell dimensions	a = 10.8574 (± 0.0005) Å<br>b = 12.5022 (± 0.0005) Å<br>c = 15.9721 (± 0.0007) Å<br>α = 86.580 (± 0.002)°<br>β = 74.824 (± 0.002)°<br>γ = 67.044 (± 0.002)°	a = 4.3494 (± 0.0013) Å<br>b = 8.693 (± 0.003) Å<br>c = 13.848 (± 0.004) Å<br>α = 90.158 (± 0.009)°<br>β = 96.904 (± 0.008)°<br>γ = 104.072 (± 0.009)°	a = 15.7878 (± 0.0006) Å<br>b = 6.1075 (± 0.0002) Å<br>c = 21.8459 (± 0.0008) Å<br>α = 90°<br>β = 101.122 (± 0.002)°<br>γ = 90°	a = 5.1327 (± 0.0002) Å<br>b = 11.6109 (± 0.0005) Å<br>c = 18.6949 (± 0.0009) Å<br>α = 90°<br>β = 96.6396 (± 0.0016)°<br>γ = 90°	a = 7.7820 (± 0.0003) Å<br>b = 13.2748 (± 0.0005) Å<br>c = 13.3139 (± 0.0004) Å<br>α = 112.7531 (± 0.0012)°<br>β = 100.3998 (± 0.0012)°<br>γ = 96.2329 (± 0.0012)°	a = 31.2256 (± 0.0010) Å<br>b = 9.2010 (± 0.0003) Å<br>c = 7.4040 (± 0.0002) Å<br>α = 90°<br>β = 100.7467 (± 0.0012)°<br>γ = 90°
Volume	1924.55 (± 0.15) Å^3	503.9 (± 0.3) Å^3	2066.91 (± 0.13) Å^3	1106.66 (± 0.08) Å^3	1223.15 (± 0.08) Å^3	2089.91 (± 0.11) Å^3
Z	2	1	6	4	2	4
Density (calculated)	2.045 g cm^{-3}	2.004 g cm^{-3}	2.434 g cm^{-3}	1.909 g cm^{-3}	2.197 g cm^{-3}	2.276 g cm^{-3}
Absorption coefficient	3.310 mm^{-1}	3.164 mm^{-1}	4.598 mm^{-1}	2.886 mm^{-1}	3.893 mm^{-1}	4.521 mm^{-1}
F(000)	1108	286	1392	604	750	1320
Crystal size	0.060 × 0.100 × 0.120 mm^3	0.040 × 0.220 × 0.240 mm^3	0.020 × 0.040 × 0.280 mm^3	0.020 × 0.040 × 0.220 mm^3	0.080 × 0.120 × 0.126 mm^3	0.020 × 0.200 × 0.240 mm^3
Theta range for data collection	2.11 to 34.96°	1.48 to 27.00°	1.90 to 36.34°	2.19 to 41.26°	2.71 to 36.35°	2.66 to 32.70°
Completeness to θ_{full}	99.7 %	99.5 %	99.7 %	99.6 %	99.5 %	98.0 %
Reflections collected	65250	5858	101700	32036	47190	3766
R_{int}	0.0865	0.0480	0.0491	0.0544	0.0532	0.0468
Independent reflections	16861	2202	12160	7401	11831	12359
Data / restrains / parameters	16861 / 0 / 487	2202 / 0 / 127	12160 / 1 / 542	7401 / 0 / 137	11831 / 0 / 307	3766 / 0 / 129
Goodness-of-fit on F^2	0.955	1.129	1.138	1.038	1.050	1.030
Final R indices [$I>2\sigma(I)$]	R_1 = 0.0458<br>wR_2 = 0.0705	R_1 = 0.0472<br>wR_2 = 0.1141	R_1 = 0.0549<br>wR_2 = 0.0937	R_1 = 0.0364<br>wR_2 = 0.0636	R_1 = 0.0413<br>wR_2 = 0.0635	R_1 = 0.0337<br>wR_2 = 0.0547
Final R indices [all data]	R_1 = 0.1101<br>wR_2 = 0.0857	R_1 = 0.0567<br>wR_2 = 0.1210	R_1 = 0.0773<br>wR_2 = 0.1043	R_1 = 0.0602<br>wR_2 = 0.0734	R_1 = 0.0742<br>wR_2 = 0.0745	R_1 = 0.0600<br>wR_2 = 0.0614
Largest diff. peak and hole	1.153 and –1.390 eÅ^{-3}	2.439 and –1.071 eÅ^{-3}	1.646 and –1.475 eÅ^{-3}	1.222 and –1.718 eÅ^{-3}	2.287 and –2.213 eÅ^{-3}	0.795 and –0.787 eÅ^{-3}

[a]All the X-ray intensity data were measured at the temperature 100 (±2) K and wavelength λ = 0.71073 Å, with Multi-Scan absorption correction.

Supplementary references

1. Monshi, A., Foroughi, M. R. & Monshi, M. R. Modified Scherrer equation to estimate more accurately nano-crystallite size using XRD. *World J. Nano Sci. Eng.* **2**, 154−160 (2012).
2. Le, H. T., Wang, C. G. & Goto, A., Solid-Phase Radical Polymerization of Halogen-Bond-Based Crystals and Applications to Pre-Shaped Polymer Materials. *Angew. Chem. Int. Ed.* **59**, 9360−9364 (2020).
3. Boyle, C. J., *et al.,* Tuning charge transport dynamics via clustering of doping in organic semiconductor thin films. *Nat. Commun.* **10**, 1−10 (2019).
4. Kissel, P., Murray, D. J., Wulftange, W. J., Catalano, V. J. & King, B. T. A nanoporous two-dimensional polymer by single-crystal-to-single-crystal photopolymerization. *Nat. Chem.* **6**, 774−778 (2014).

Author Information

ORCID of Hong Tho Le: 0000-0002-0043-1404

ORCID of Chen-Gang Wang: 0000-0001-6986-3961

ORCID of Atsushi Goto: 0000-0001-7643-3169
